# Supplementary material for: FMRP regulates neuronal RNA granules containing stalled ribosomes, not where ribosomes stall
Source: eLife. 2026 Jul 20;14:RP106692. doi: 10.7554/eLife.106692 (PMC13384498; doi:10.7554/eLife.106692)
Supplement: Figure 1—source data 1. [file elife-106692-fig1-data1.pdf]

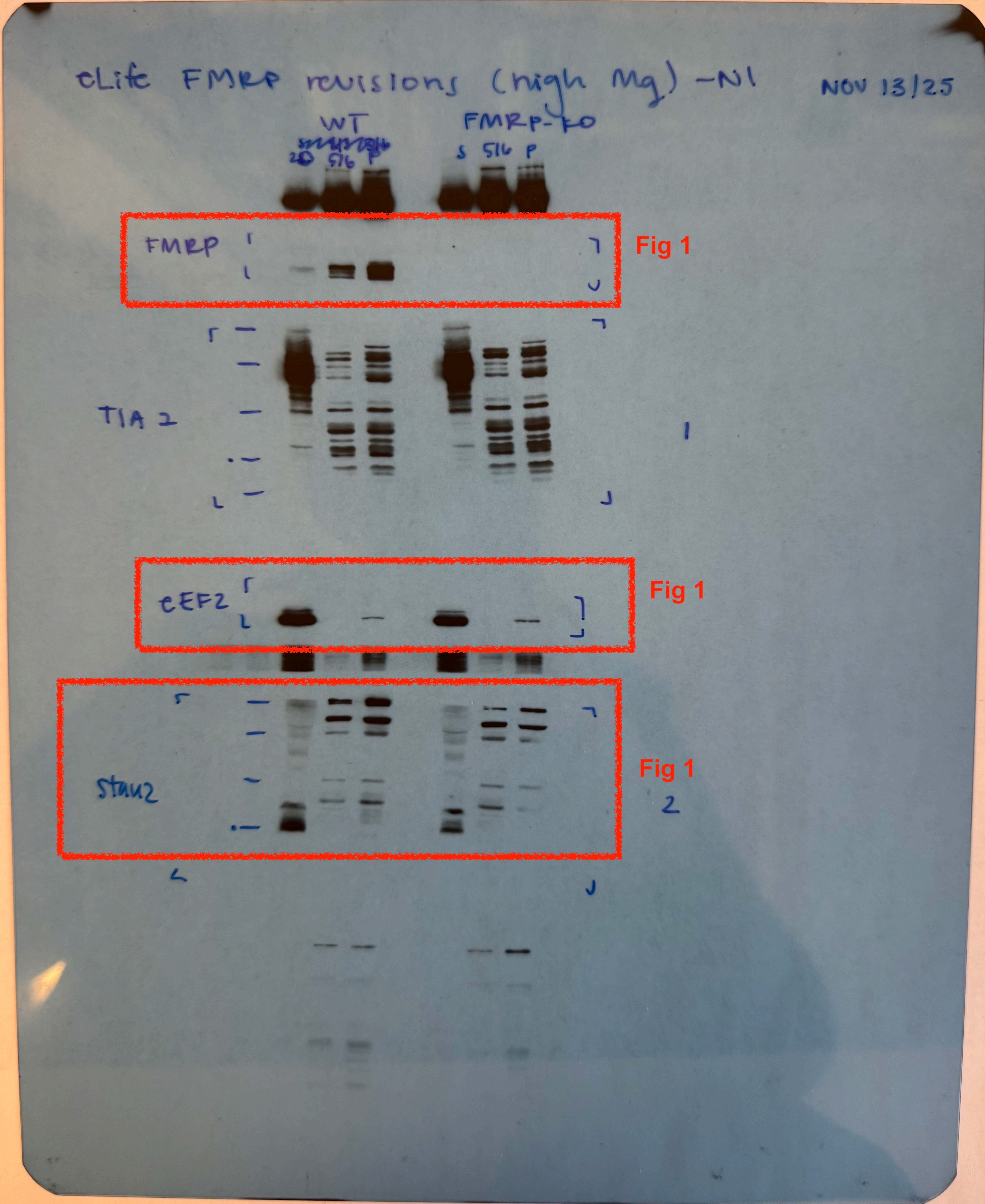
eLife FMRP revisions (high Mg) -N1
NOV 13/25
WT
FMRP-KO
S 516 P
FMRP
Fig 1
TIA 2
eEF2
Fig 1
Stau2
Fig 1
2

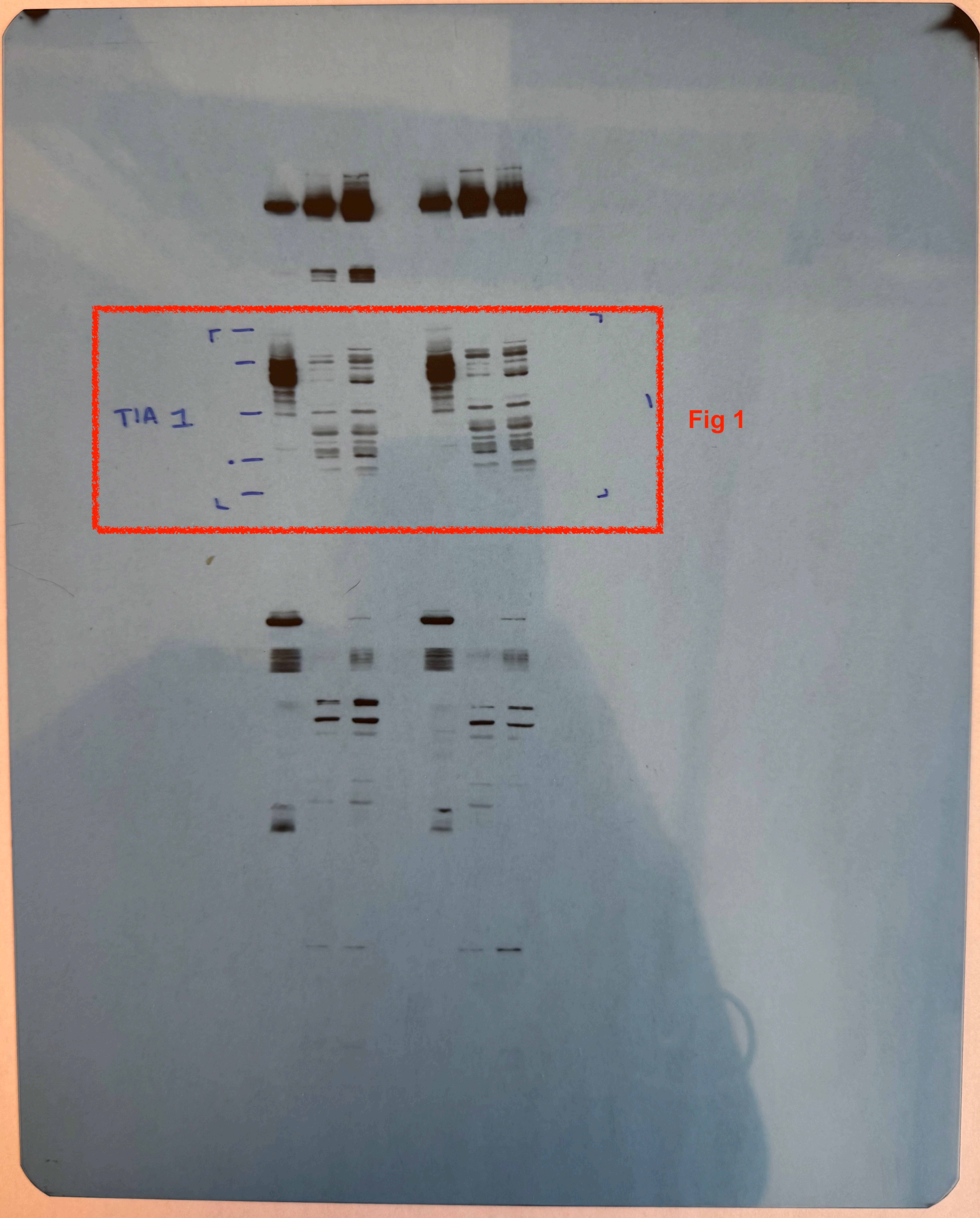
TIA 1
Fig 1

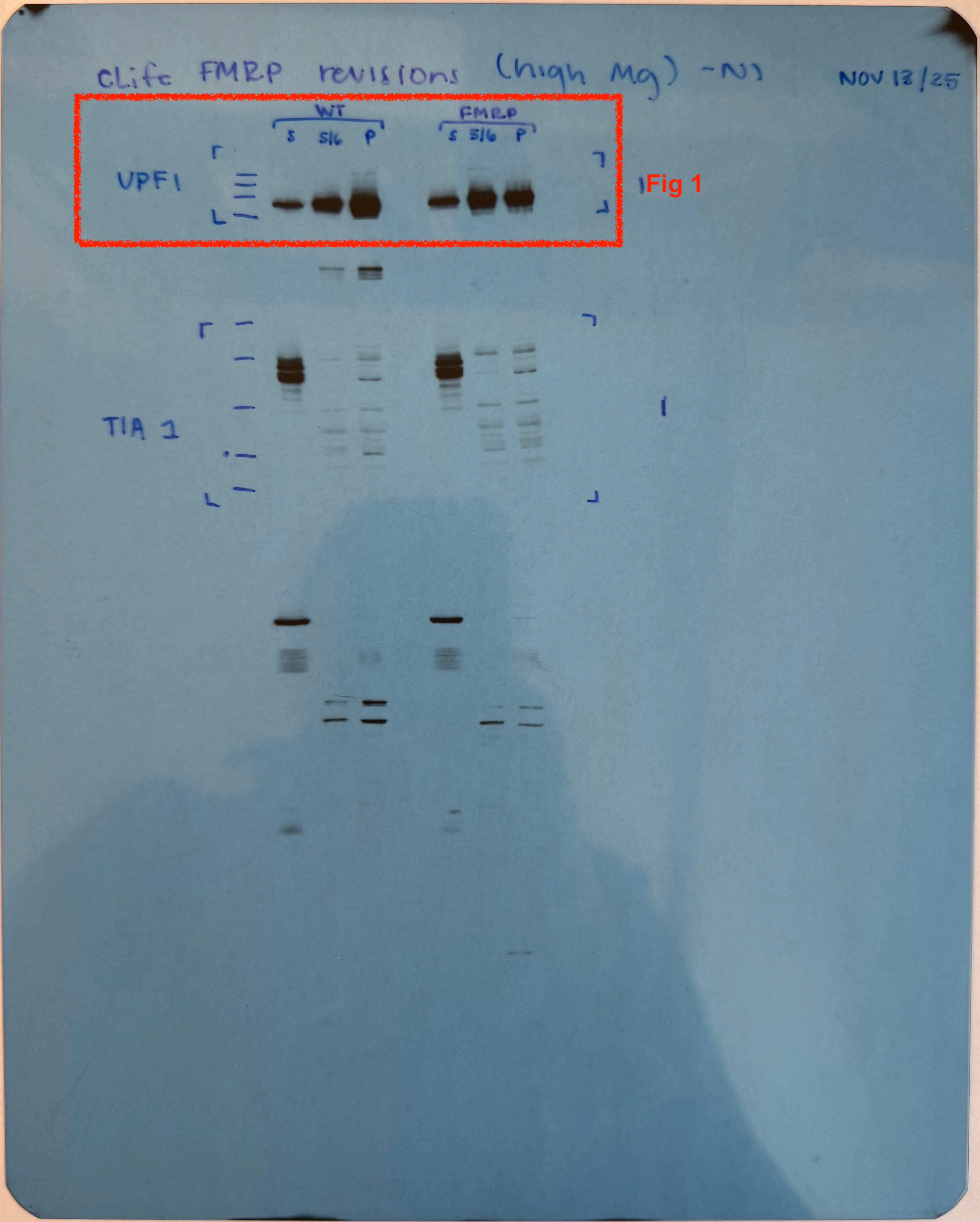

CLifc FMRP revisions (high Mg) -NJ
NOV 13/25
WT
S 5/6 P
FMRP
S 5/6 P
UPF1
Fig 1
TIA 1

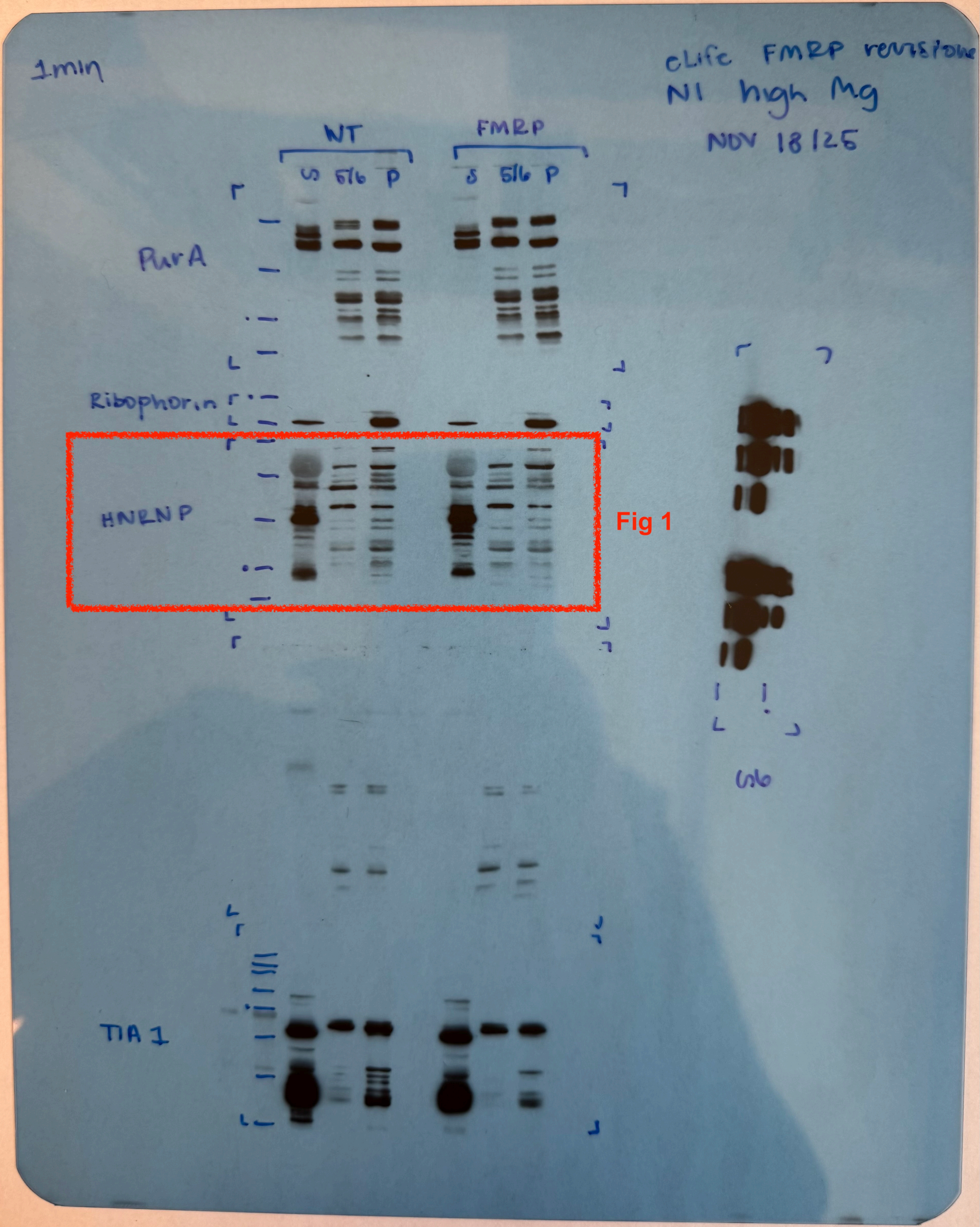
1min
cLife FMRP revisions
N1 high Mg
NOV 18/25
WT
FMRP
S 5/6 P
S 5/6 P
PurA
Ribophorin
HNRNP
Fig 1
TIA 1
U6

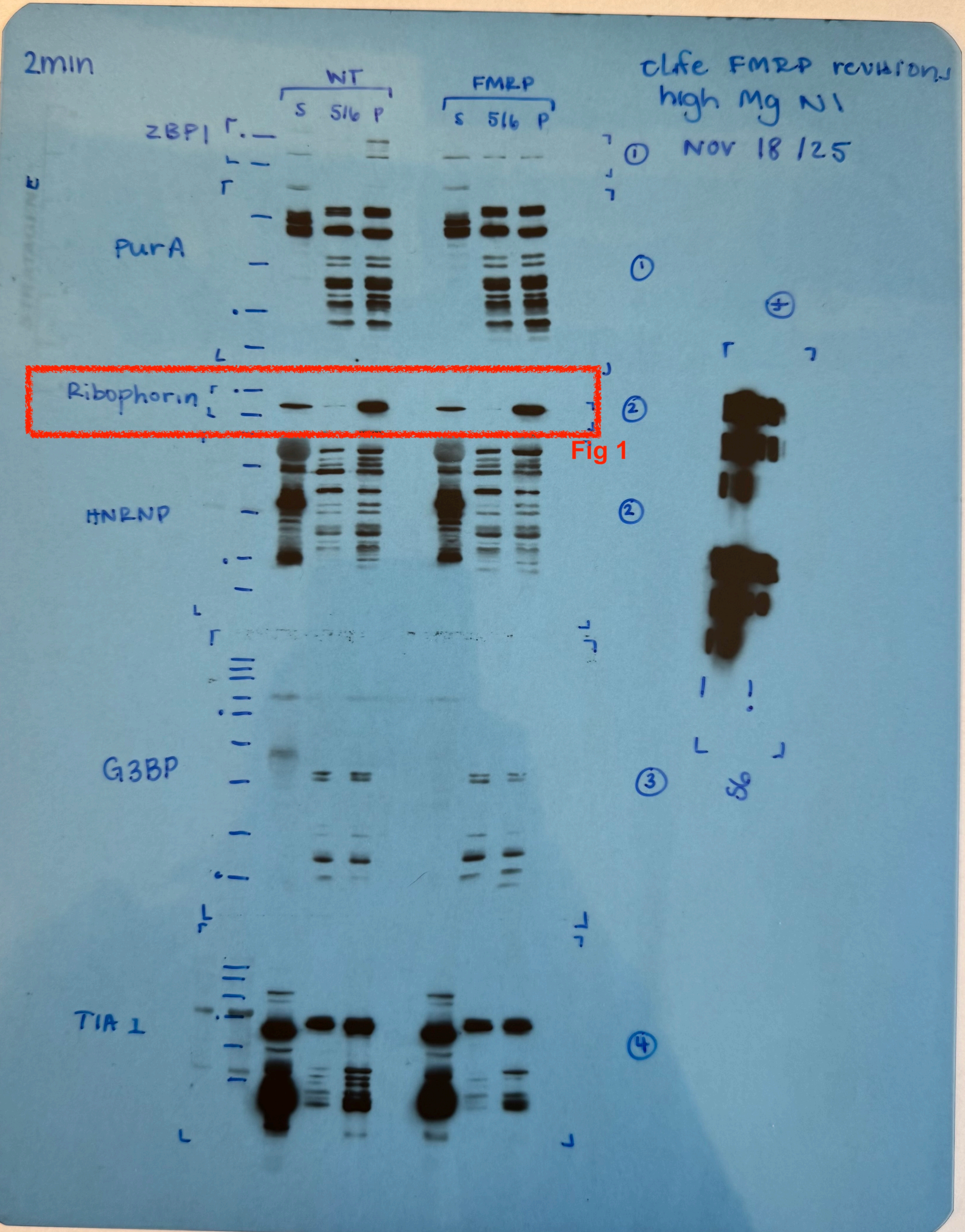
2min
WT
S 5/6 P
FMRP
S 5/6 P
clfe FMRP revisions
high Mg N1
Nov 18 /25
ZBP1
PurA
Ribophorin
Fig 1
HNRNP
G3BP
TIA 1
①
①
②
②
③
④
58

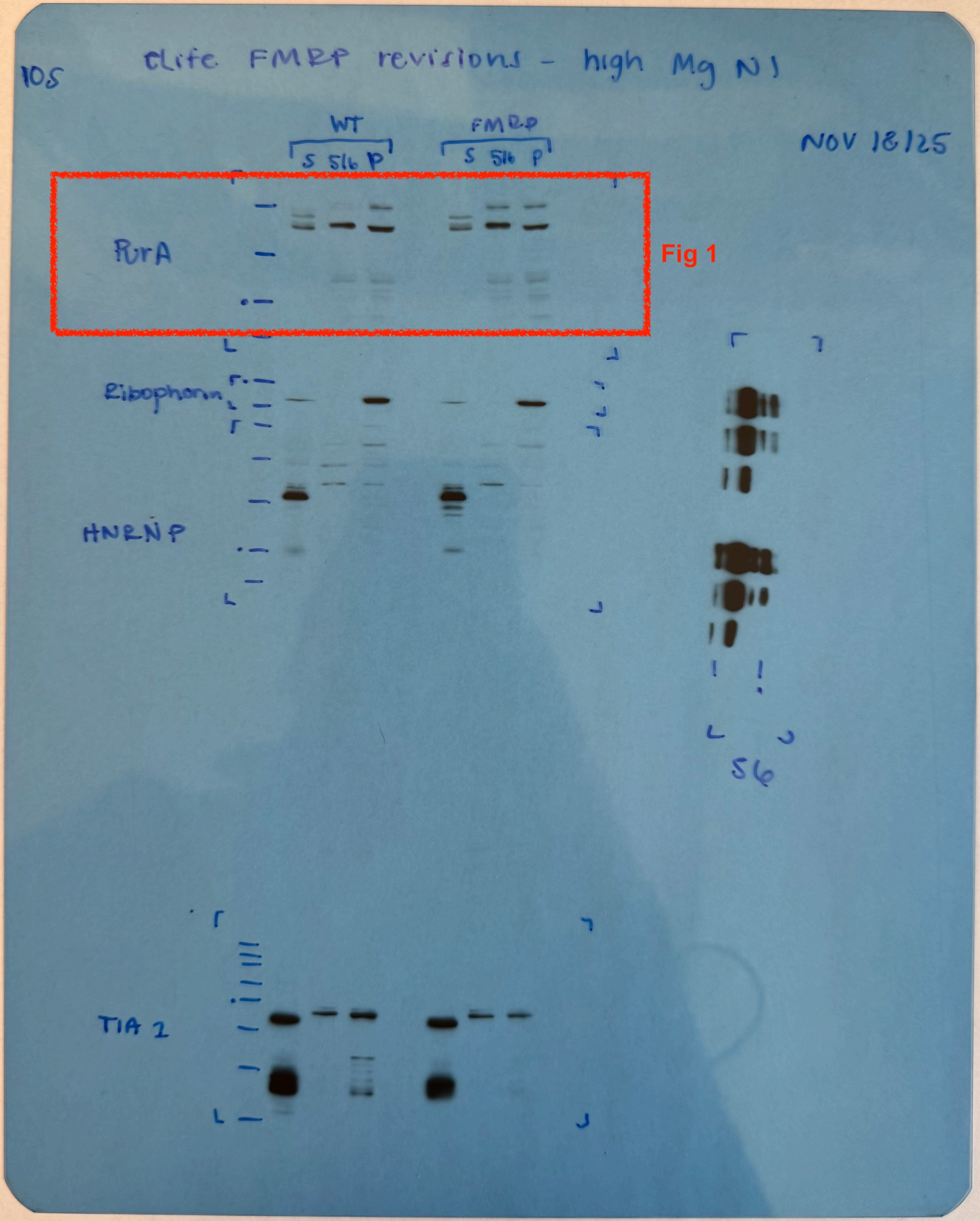
105
eLife FMRP revisions - high Mg NJ
NOV 18/25
WT
S 516 P
FMRP
S 516 P
PurA
Fig 1
Ribophorin
HNRNP
TIA 2
56

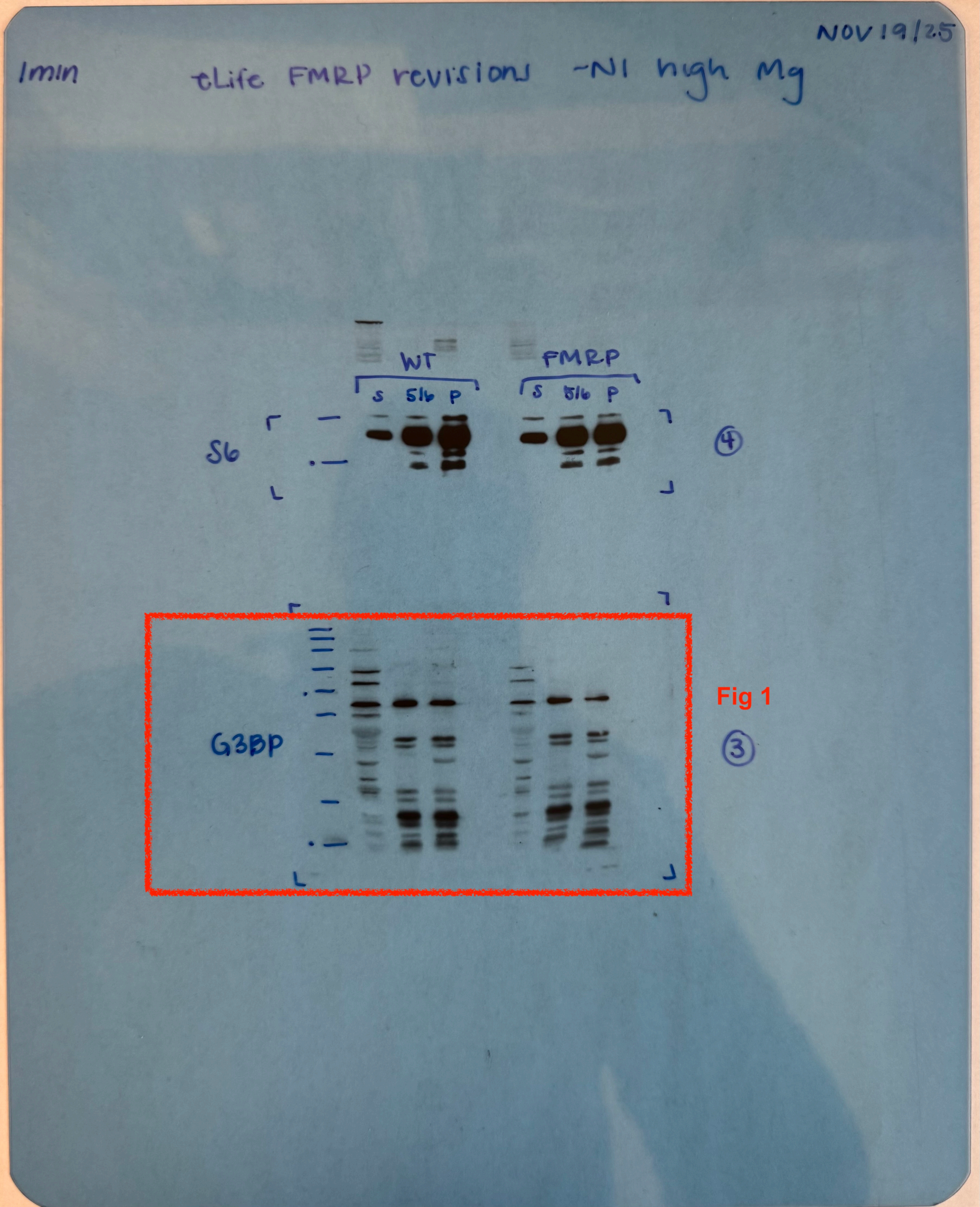
NOV 19/25
1min
eLife FMRP revisions -N1 high Mg
WT
FMRP
S
5/6
P
S
5/6
P
S6
4
G3BP
Fig 1
3

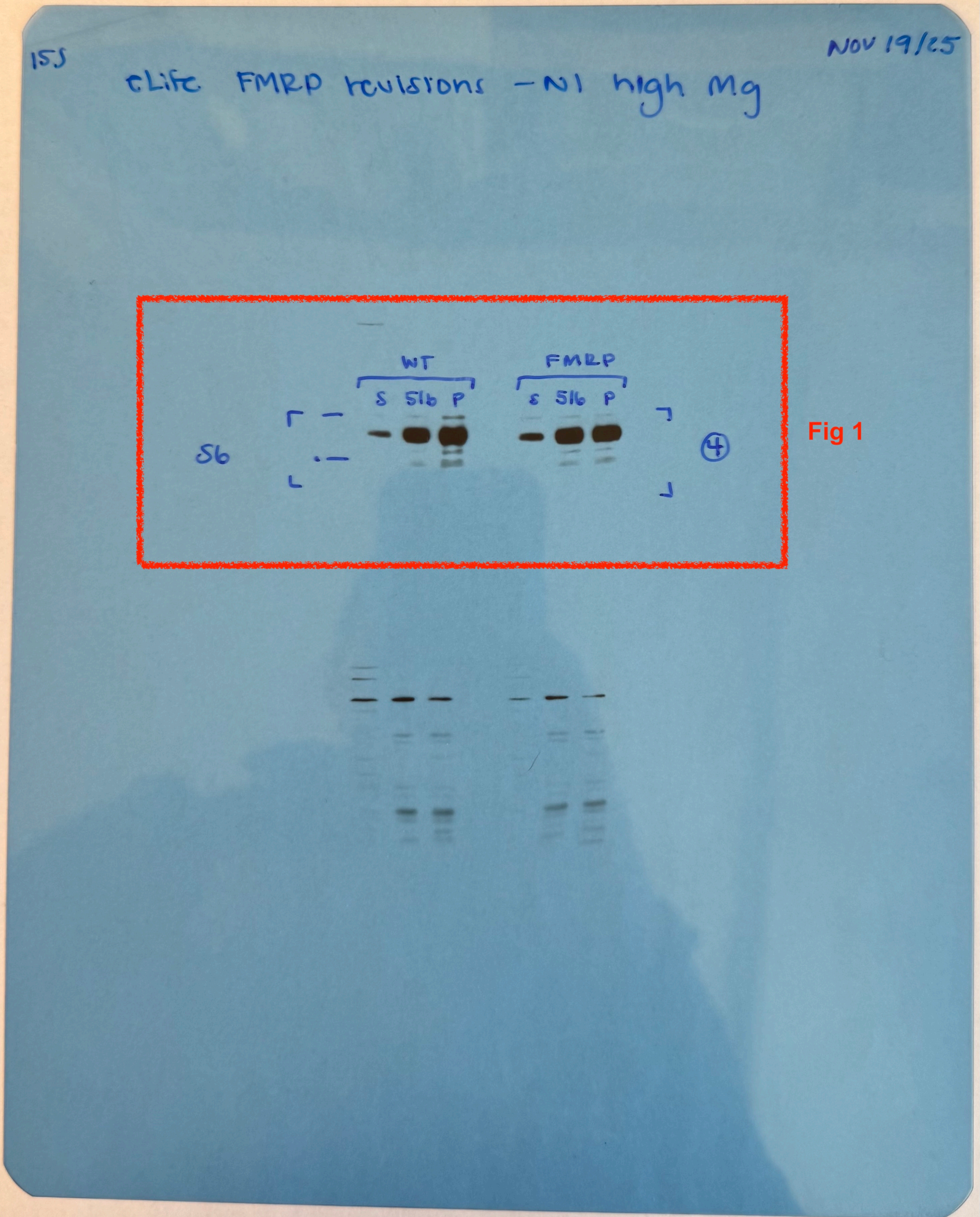
15S
Nov 19/25
eLife FMRP revisions - N1 high Mg
WT
FMRP
S 516 P
S 516 P
S6
4
Fig 1

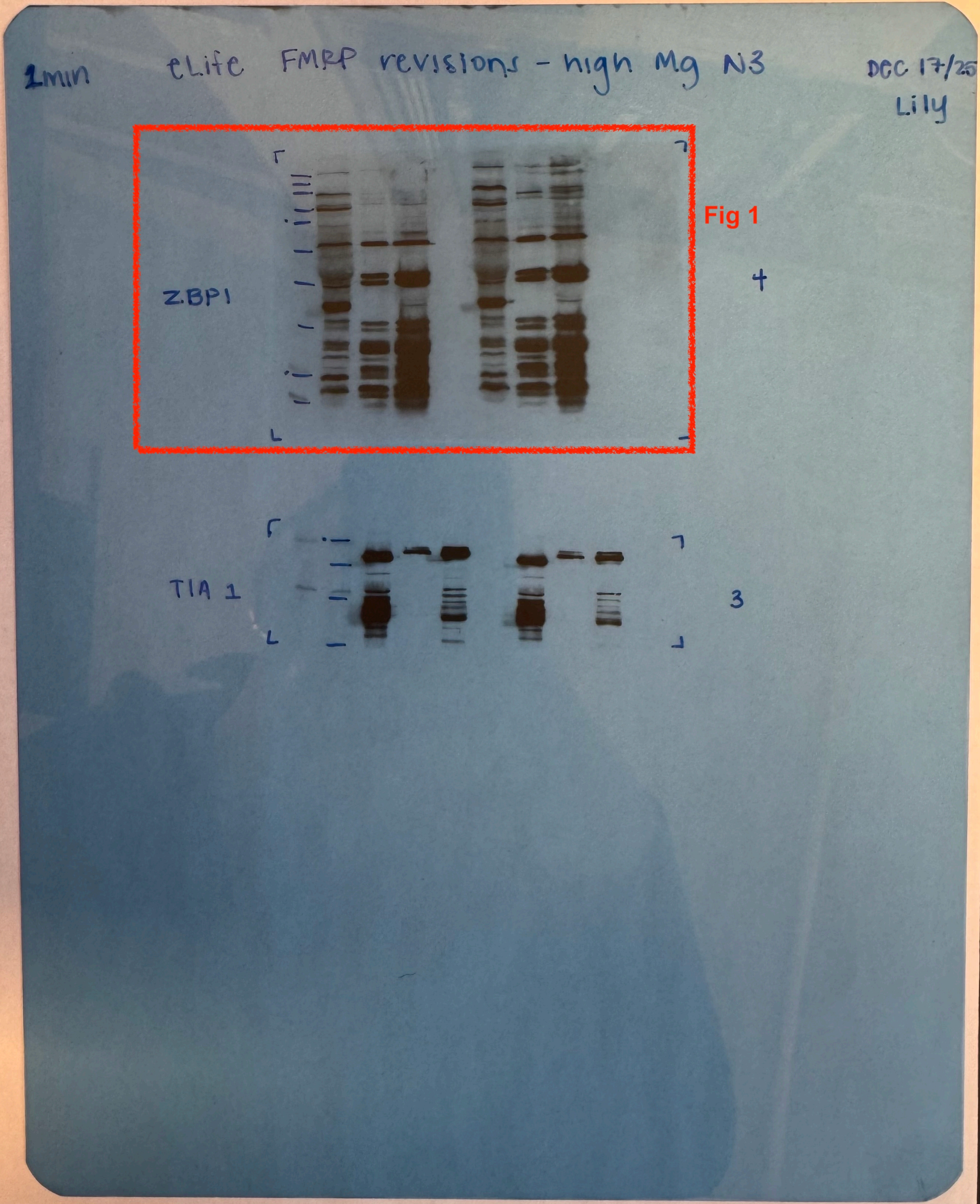
1min
eLife FMRP revisions - high Mg N3
Dec 17/25
Lily
Fig 1
ZBP1
4
TIA 1
3
