# Supplementary figures and images for: FMRP regulates neuronal RNA granules containing stalled ribosomes, not where ribosomes stall
Source: eLife. 2026 Jul 20;14:RP106692. doi: 10.7554/eLife.106692 (PMC13384498; doi:10.7554/eLife.106692)

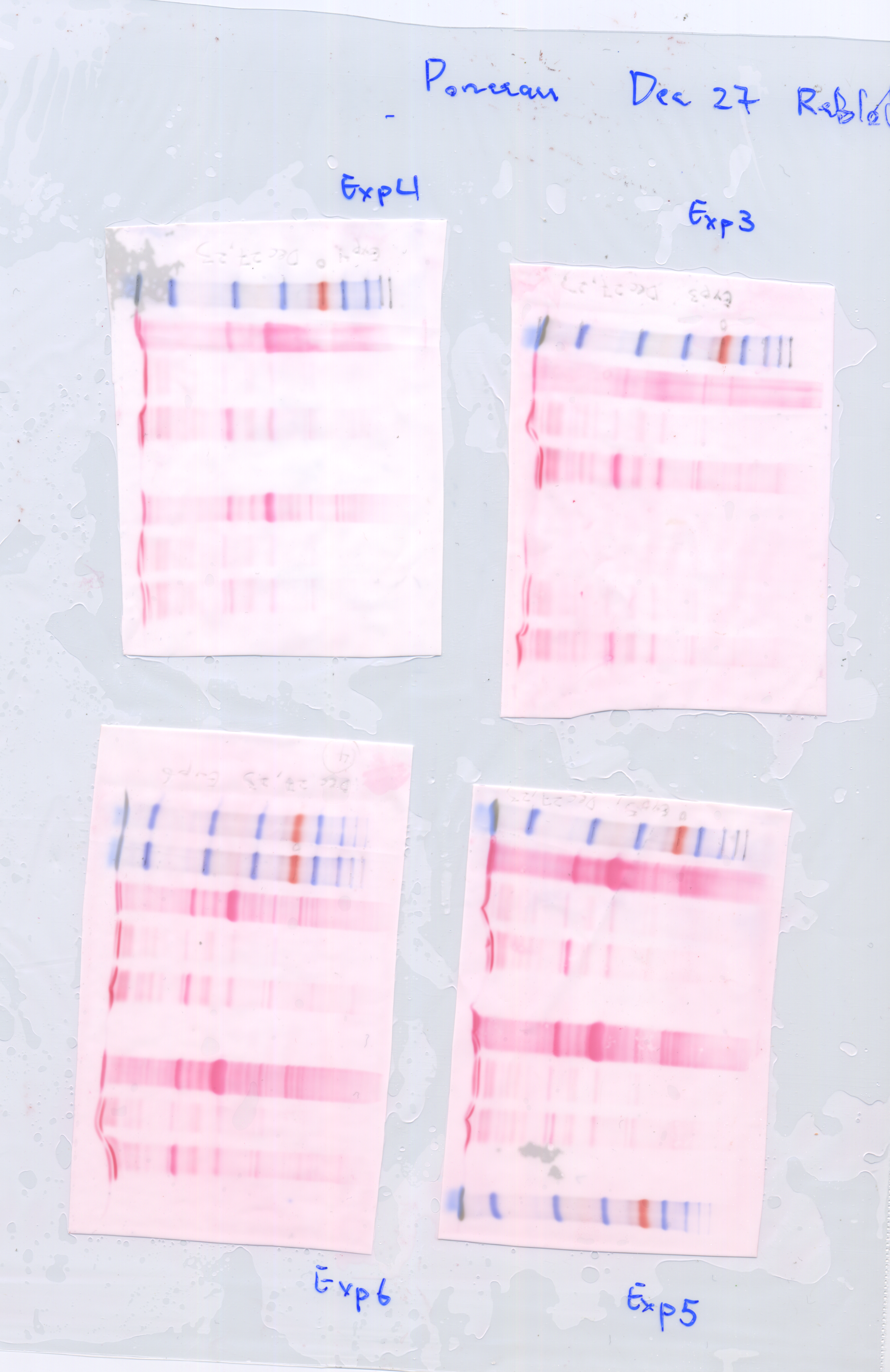

Supplement: Figure 1—source data 2. [file elife-106692-fig1-data2.zip › Dec 27 ponceau exp 3 4 5 6 Comp C57 FMR1 10% gel 1.tiff]

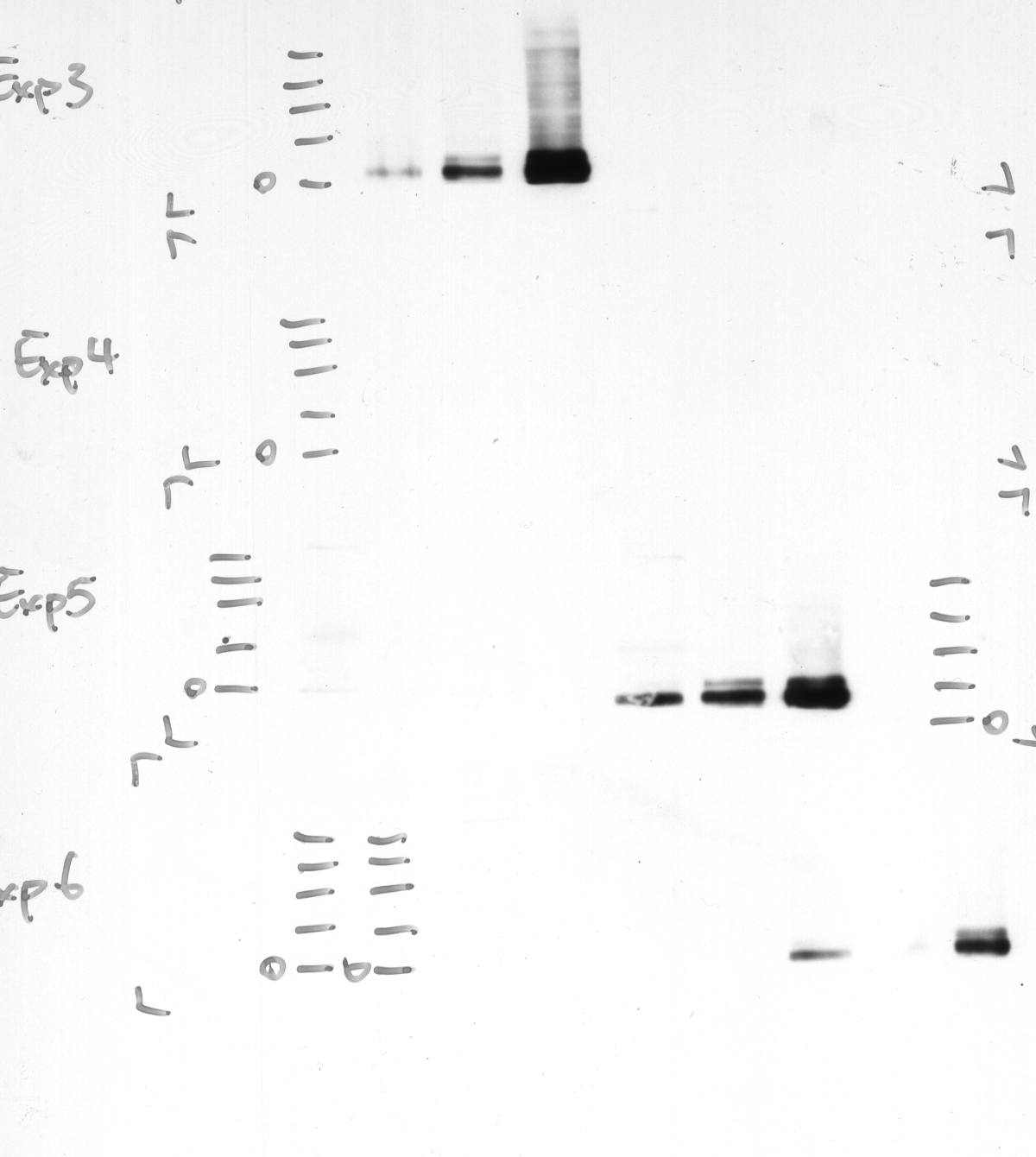

Supplement: Figure 1—source data 2. [file elife-106692-fig1-data2.zip › Dec 28 anti FMR1 FMRP vs C57 45 sec.tiff]

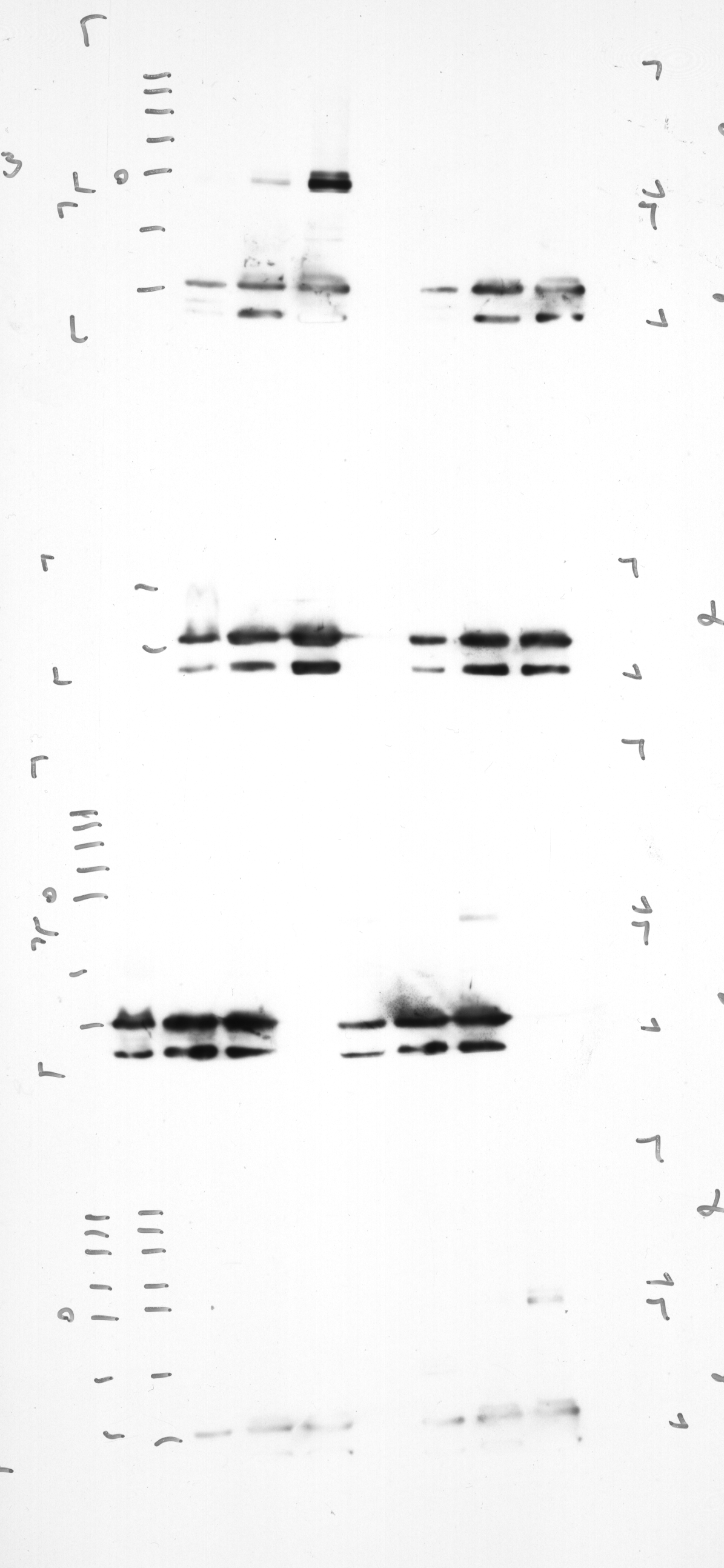

Supplement: Figure 1—source data 2. [file elife-106692-fig1-data2.zip › Dec 28 anti-puralpha FMRP vs C57 15sec.tiff]

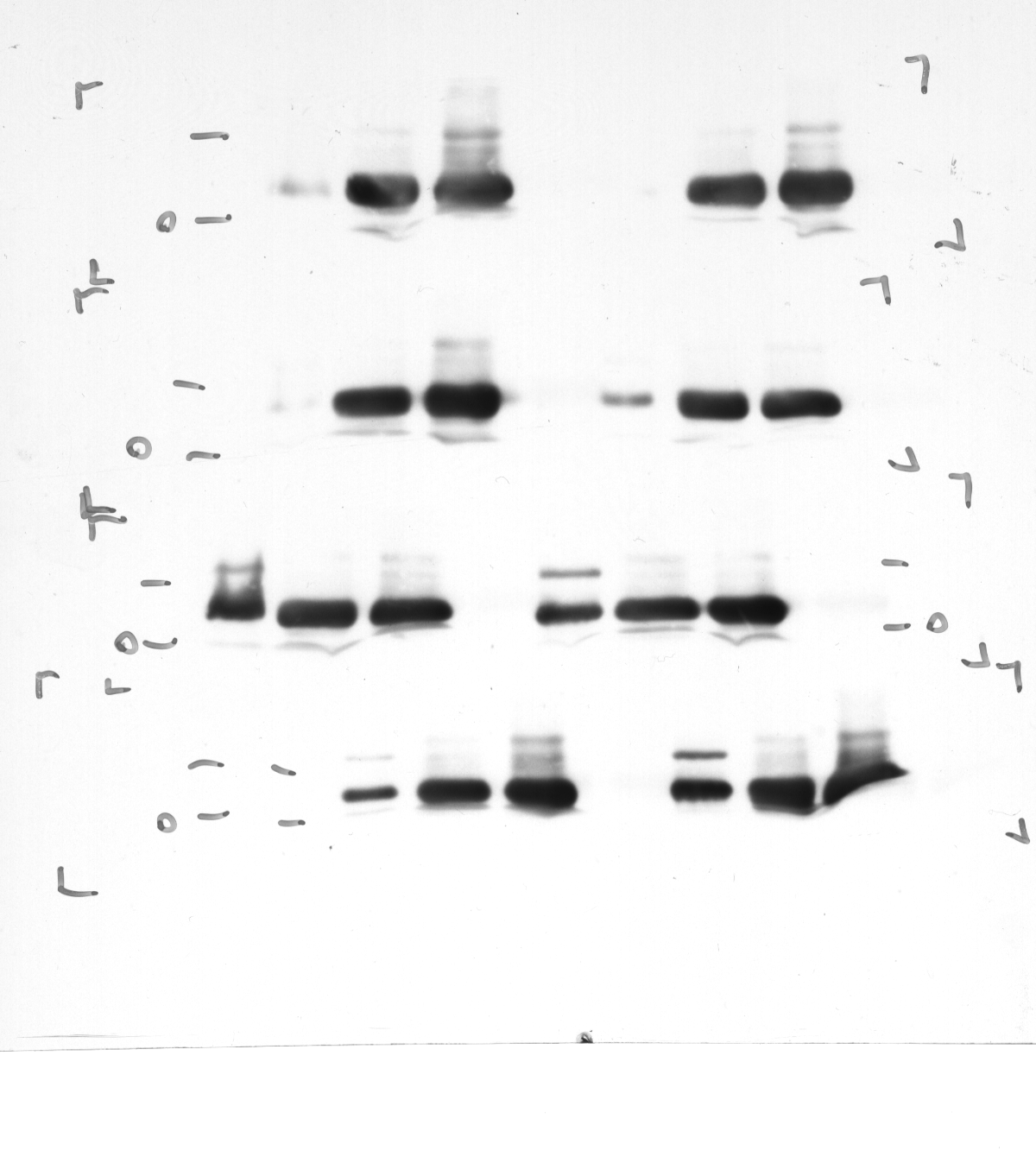

Supplement: Figure 1—source data 2. [file elife-106692-fig1-data2.zip › Dec 28 anti-s6 FMRP vs C57 15sec.tiff]

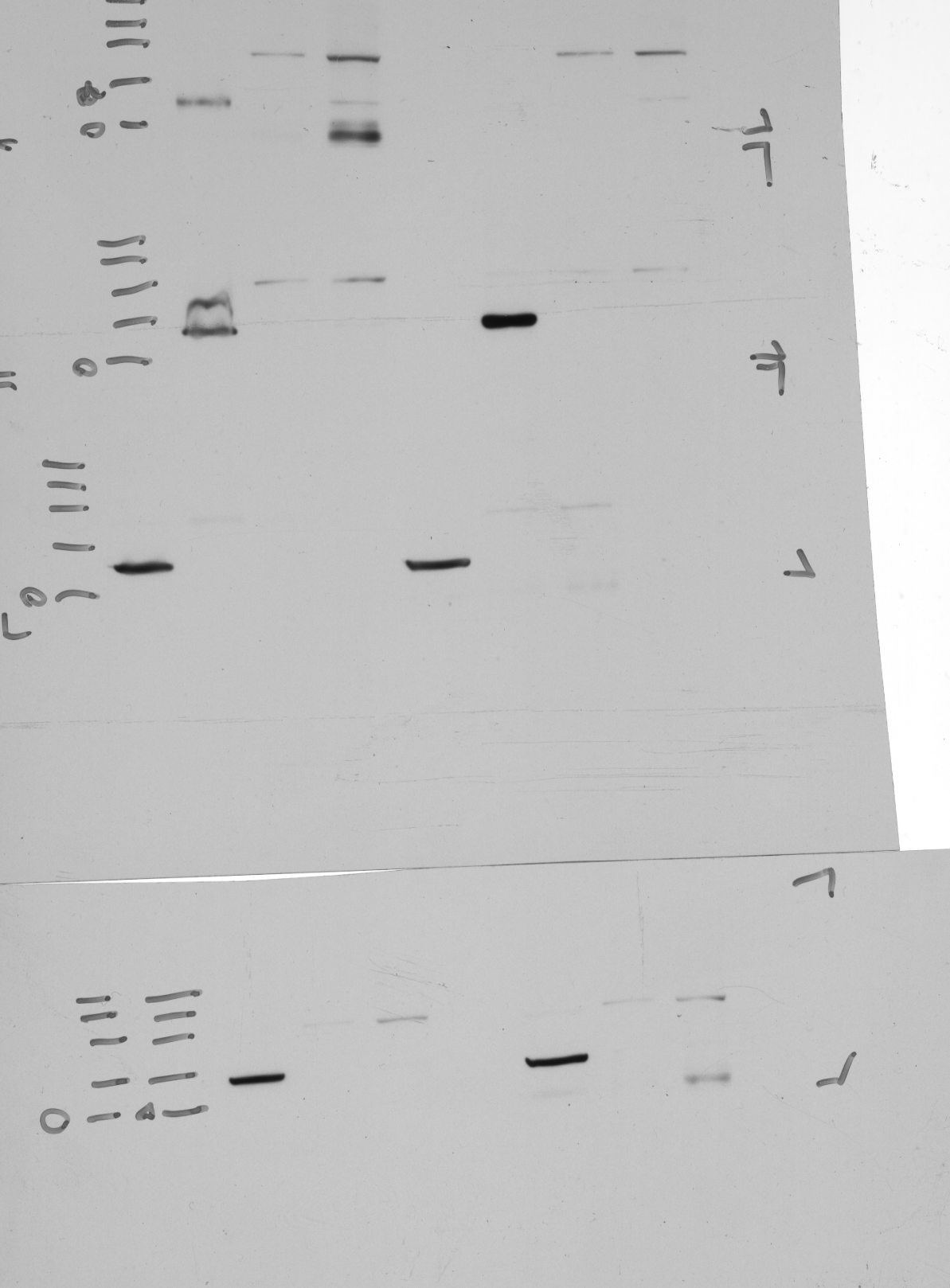

Supplement: Figure 1—source data 2. [file elife-106692-fig1-data2.zip › Dec 29 anti-eEF2 45sec.tiff]

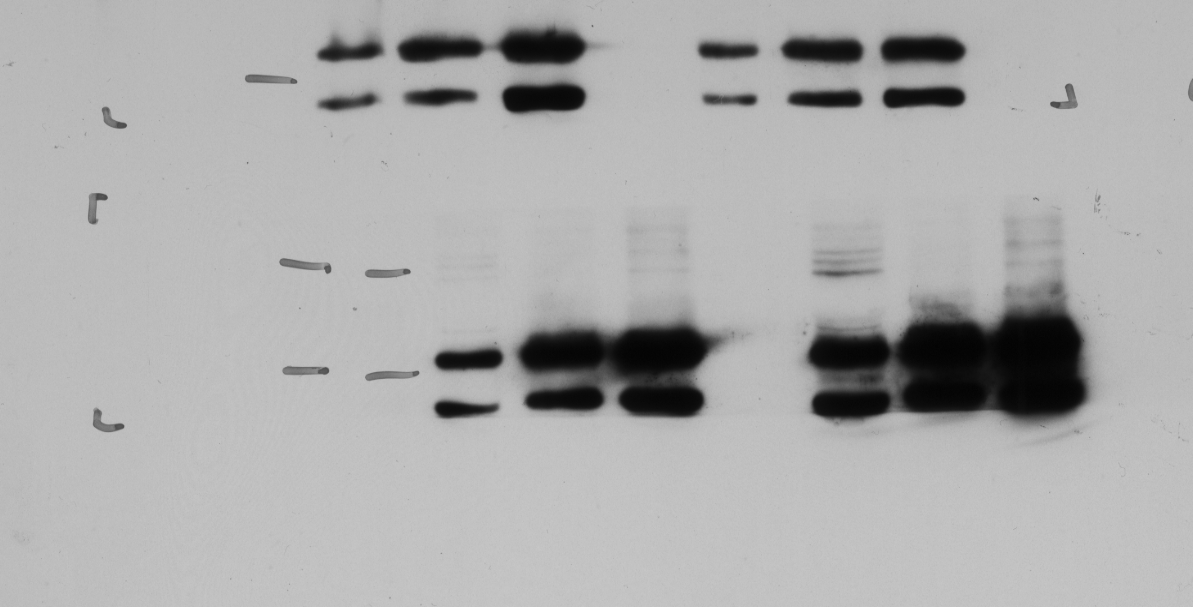

Supplement: Figure 1—source data 2. [file elife-106692-fig1-data2.zip › Dec 29 anti-puralpha exp 4 6 45sec.tiff]

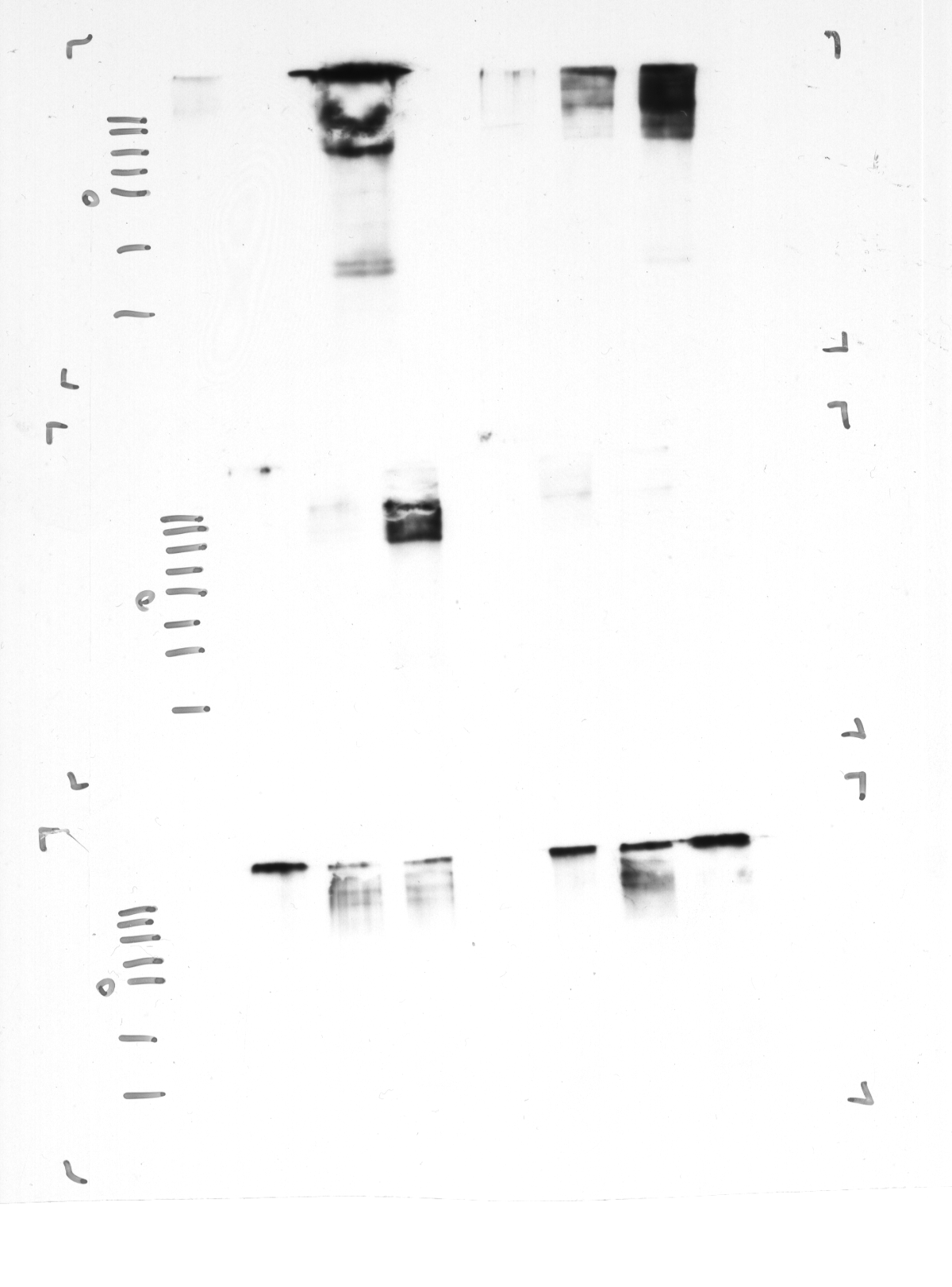

Supplement: Figure 1—source data 2. [file elife-106692-fig1-data2.zip › Dec 29 anti-upf1 fmrp stau2 Mg2+ vs N 45sec.tiff]

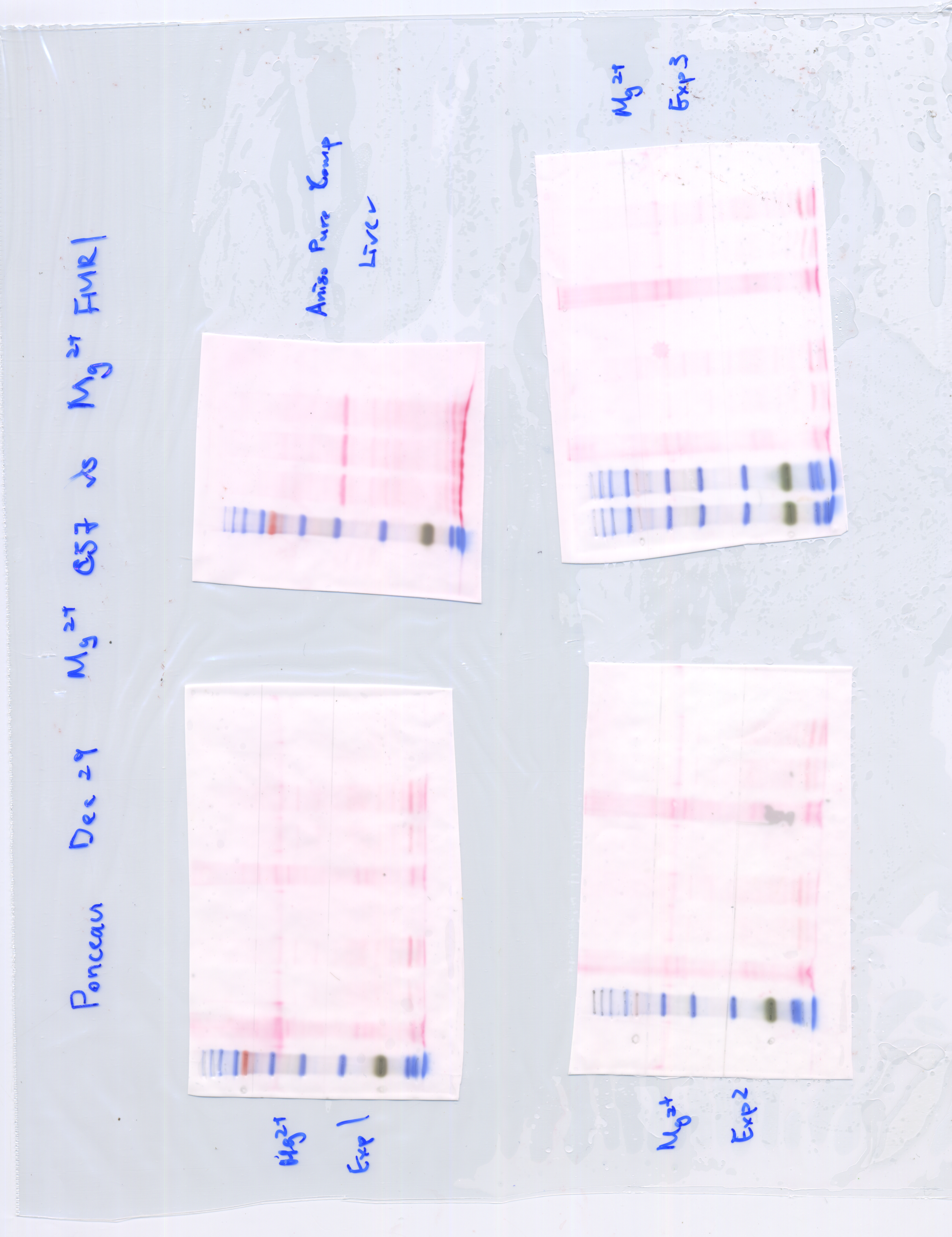

Supplement: Figure 1—source data 2. [file elife-106692-fig1-data2.zip › Dec 29 ponceau Mg2+ exp 1 2 3 C57 FMR1 .tiff]

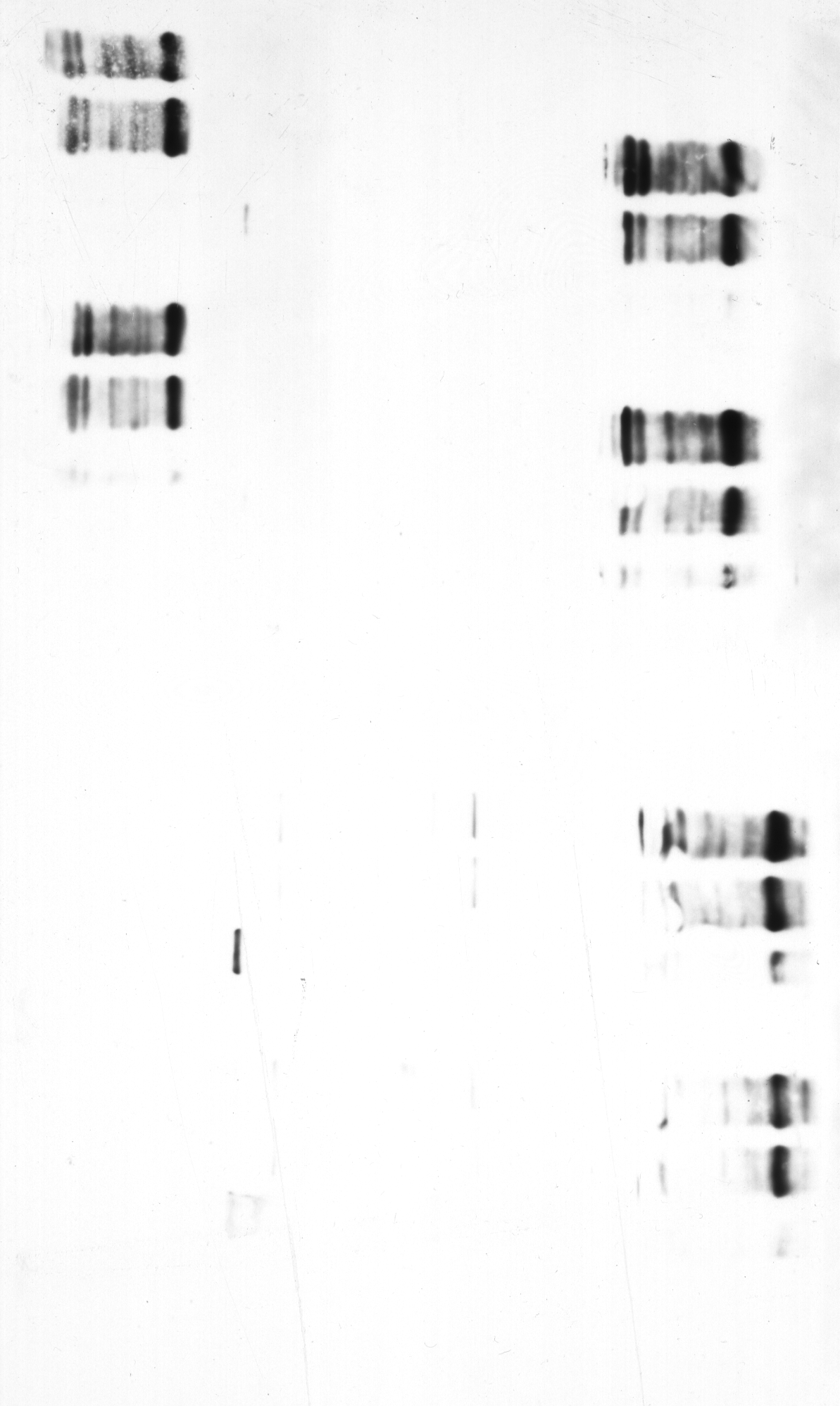

Supplement: Figure 1—source data 2. [file elife-106692-fig1-data2.zip › Dec 30 anti-s6 Mg2+FMR1 vs C57 .tiff]

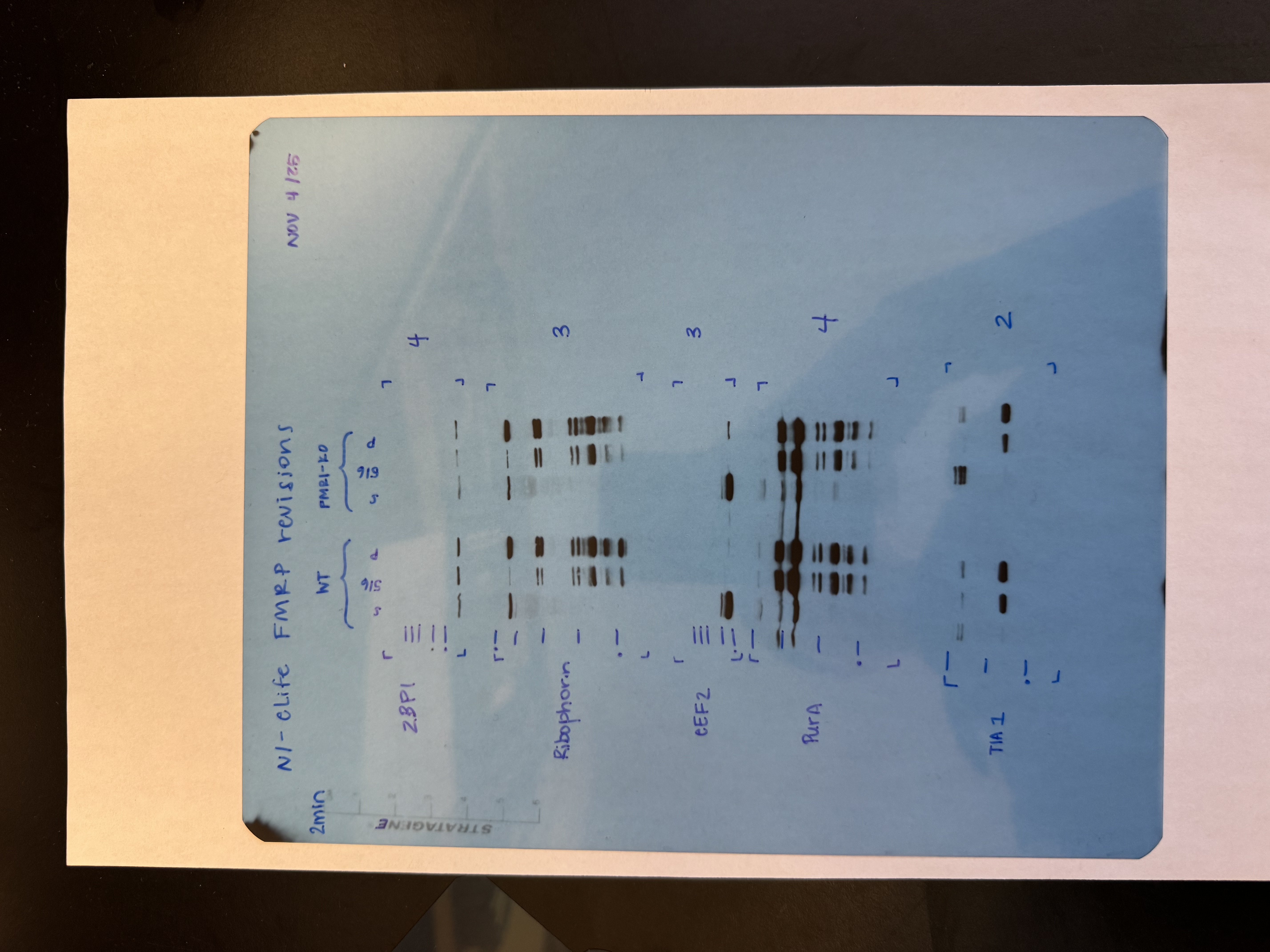

Supplement: Figure 1—source data 2. [file elife-106692-fig1-data2.zip › M1 Nov 4 2 min raw.jpeg]

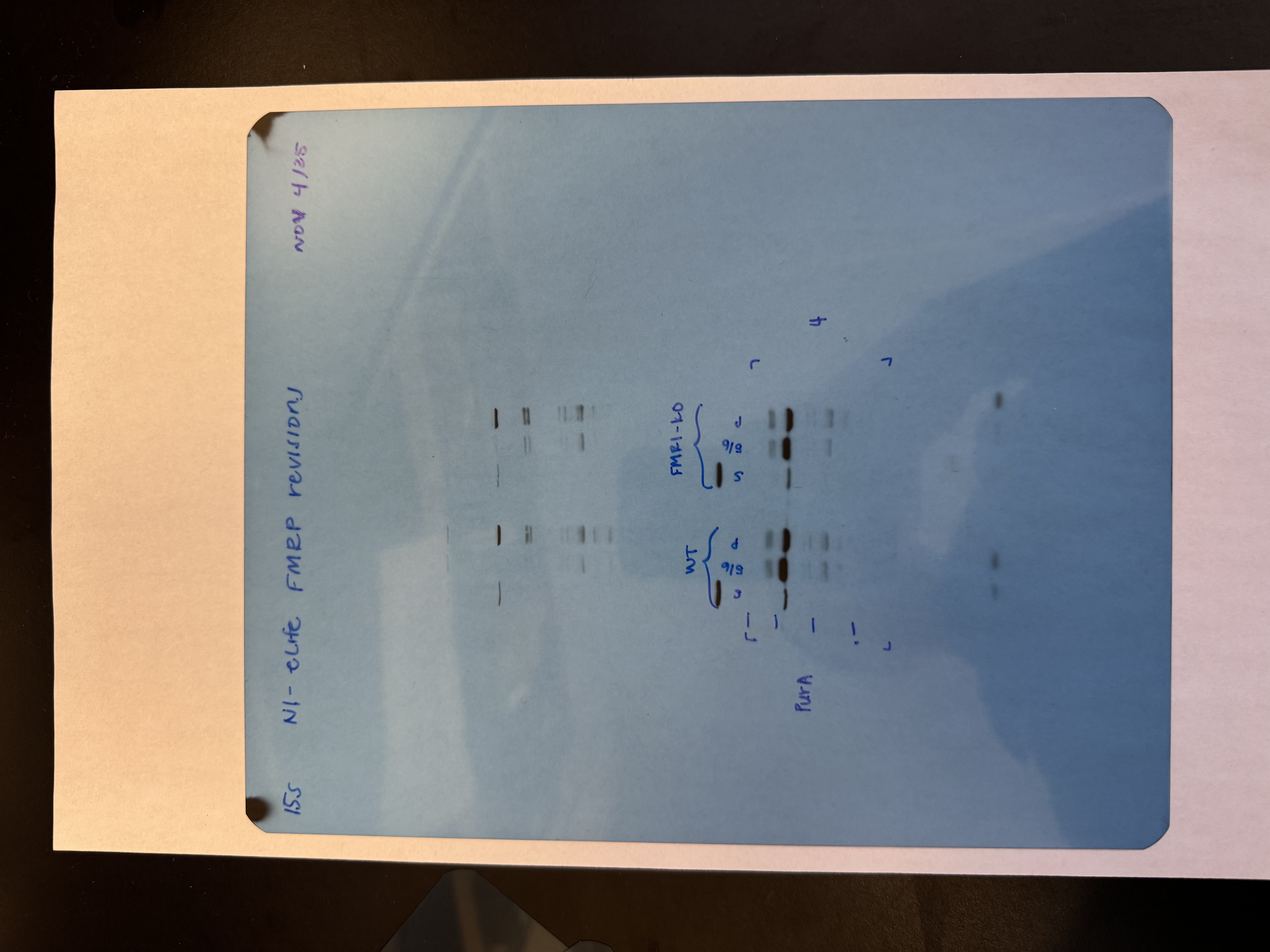

Supplement: Figure 1—source data 2. [file elife-106692-fig1-data2.zip › M1 Nov 4 15s raw.jpeg]

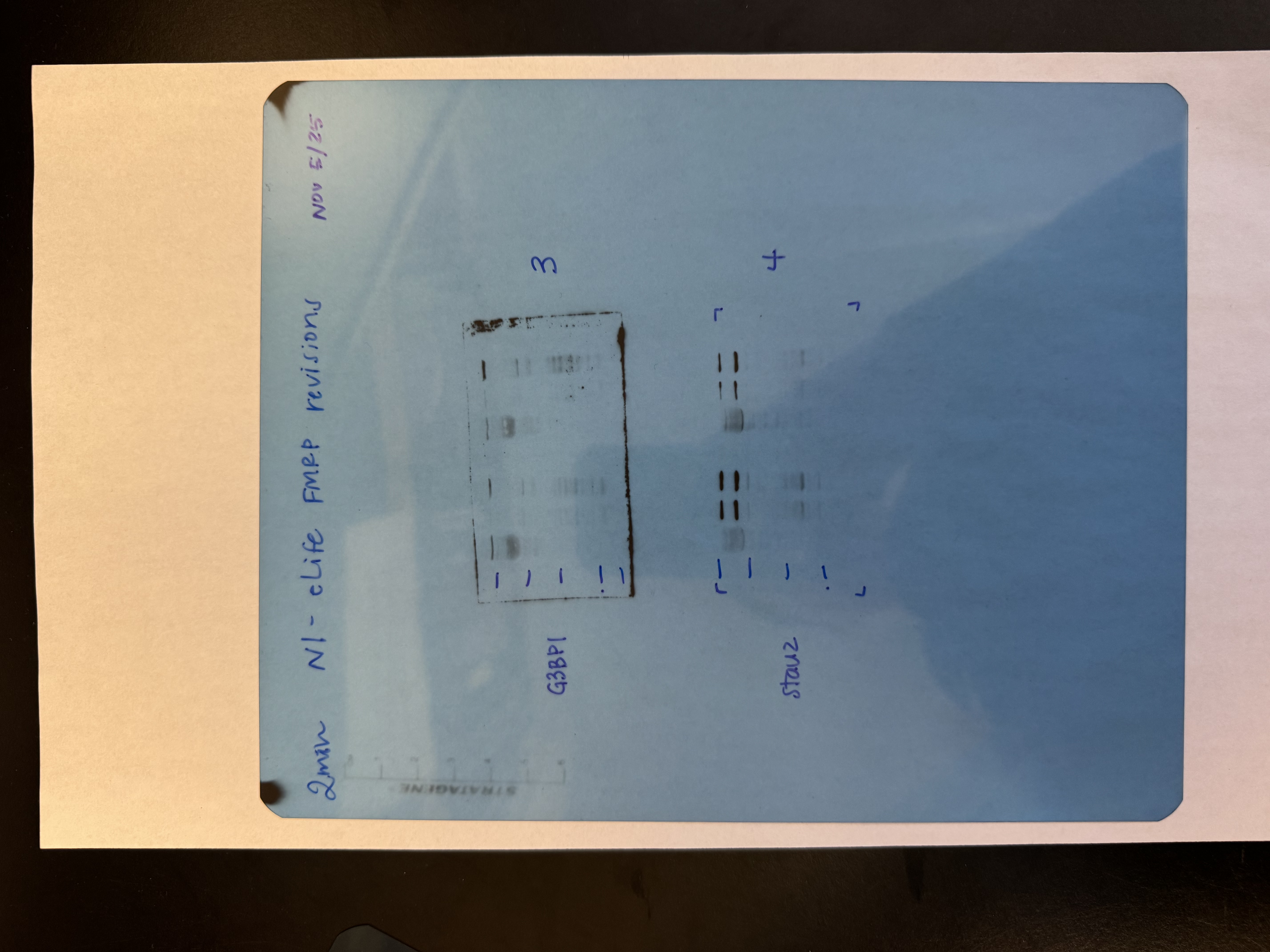

Supplement: Figure 1—source data 2. [file elife-106692-fig1-data2.zip › M1 Nov 5 2 min raw.jpeg]

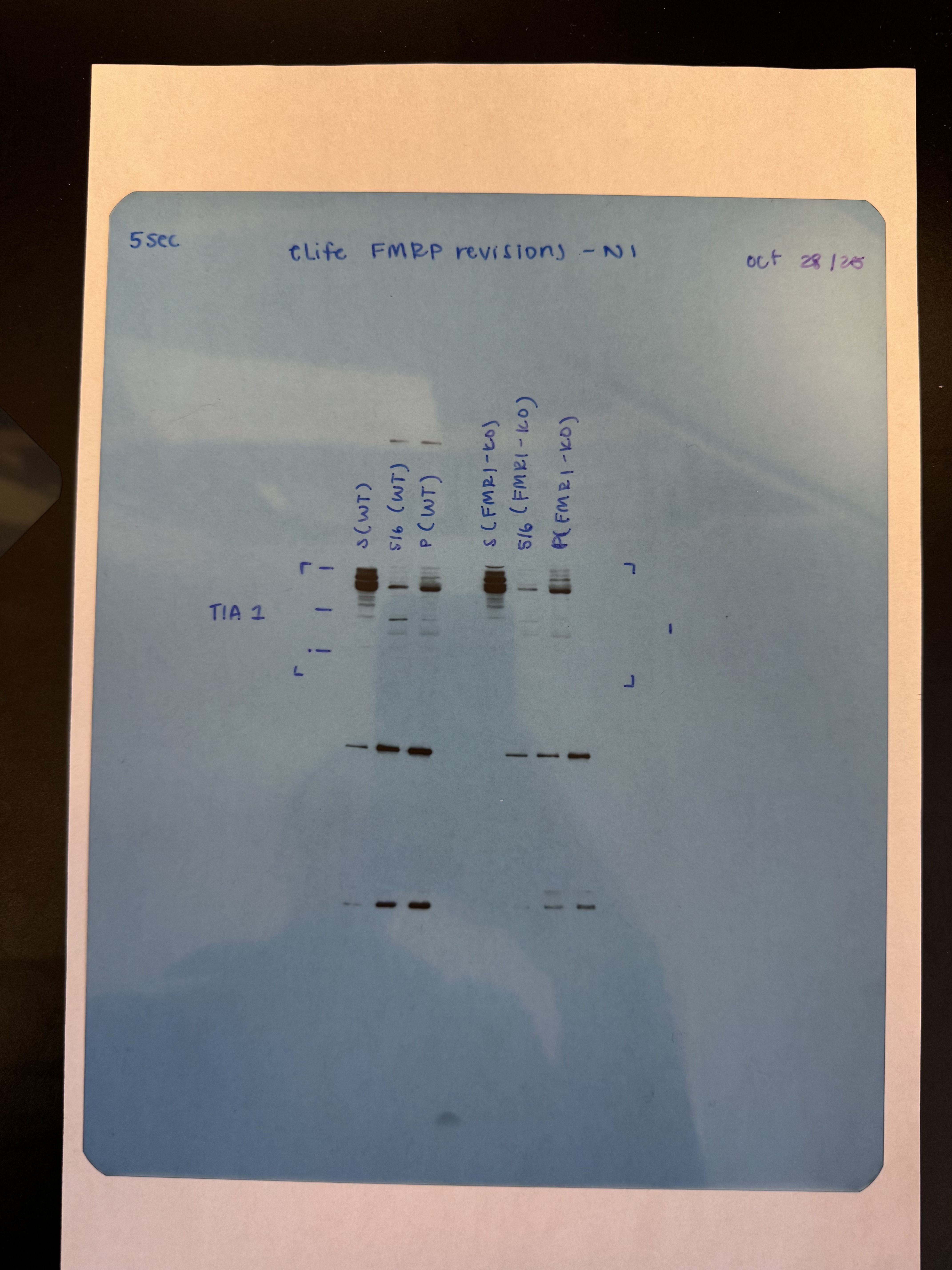

Supplement: Figure 1—source data 2. [file elife-106692-fig1-data2.zip › M2 oct 28 5s raw.jpeg]

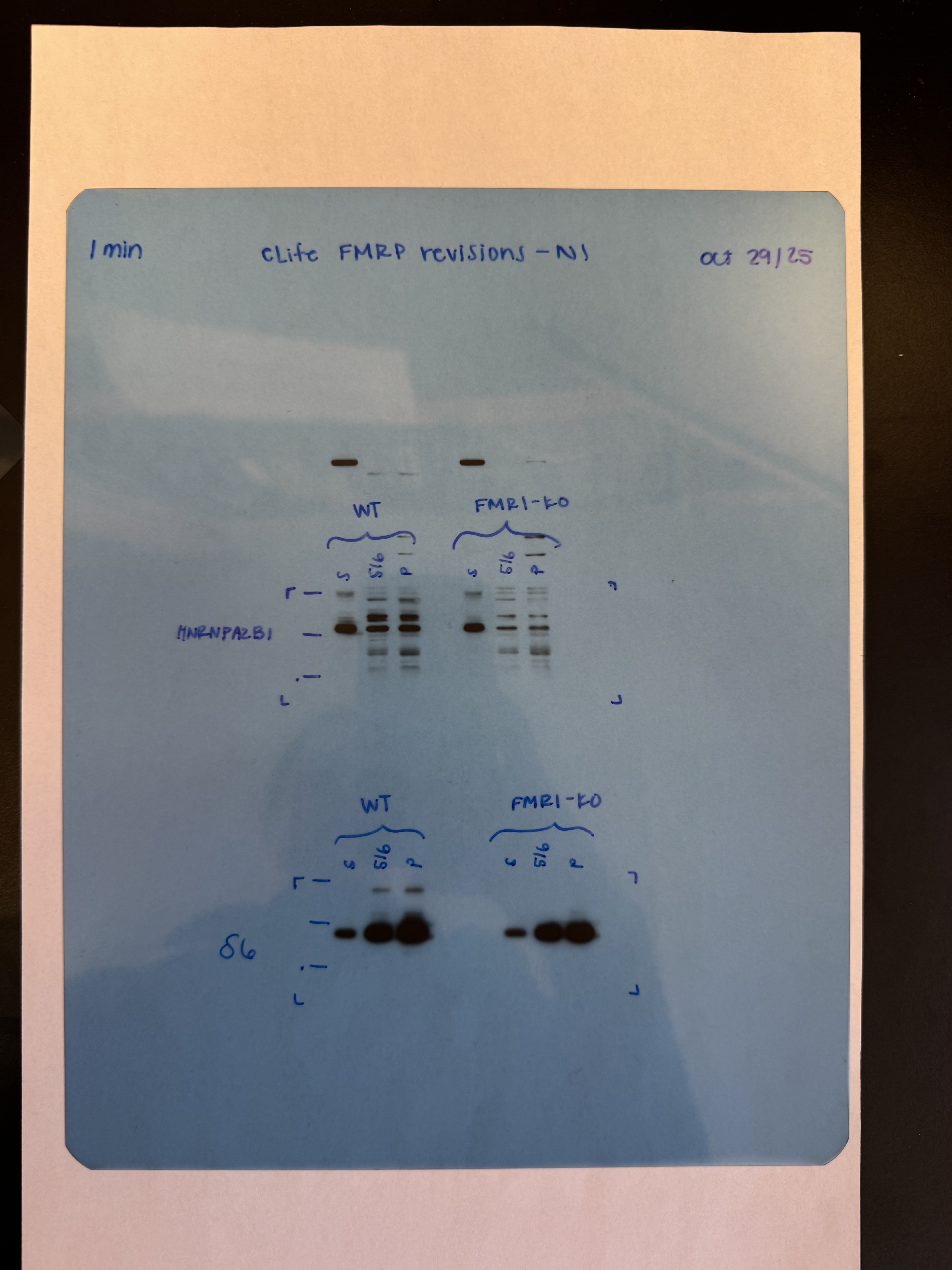

Supplement: Figure 1—source data 2. [file elife-106692-fig1-data2.zip › M2 Oct 29 1 min raw.jpeg]

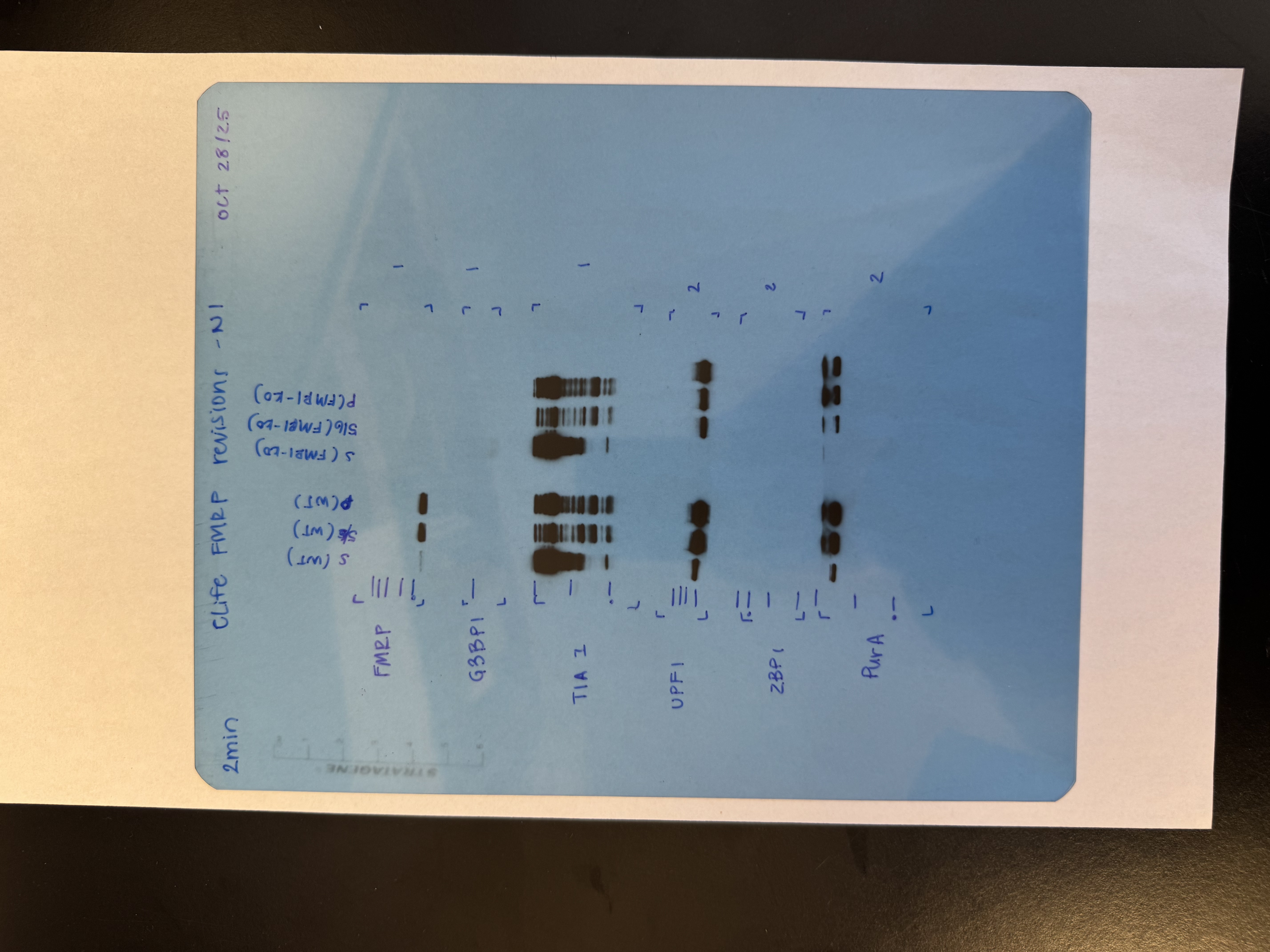

Supplement: Figure 1—source data 2. [file elife-106692-fig1-data2.zip › M2 Oct 28 2 min raw.jpeg]

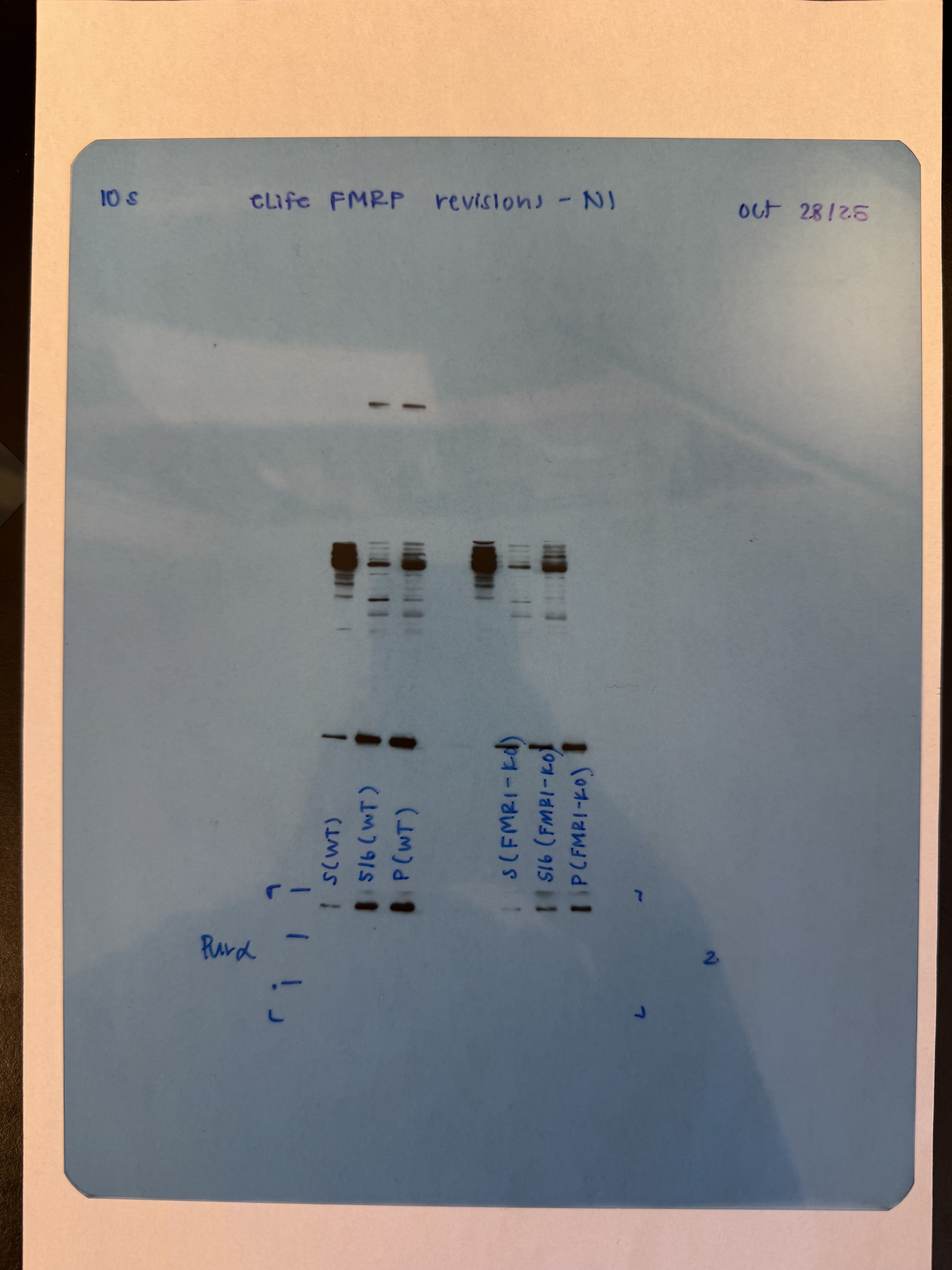

Supplement: Figure 1—source data 2. [file elife-106692-fig1-data2.zip › M2 Oct 28 10s raw.jpeg]

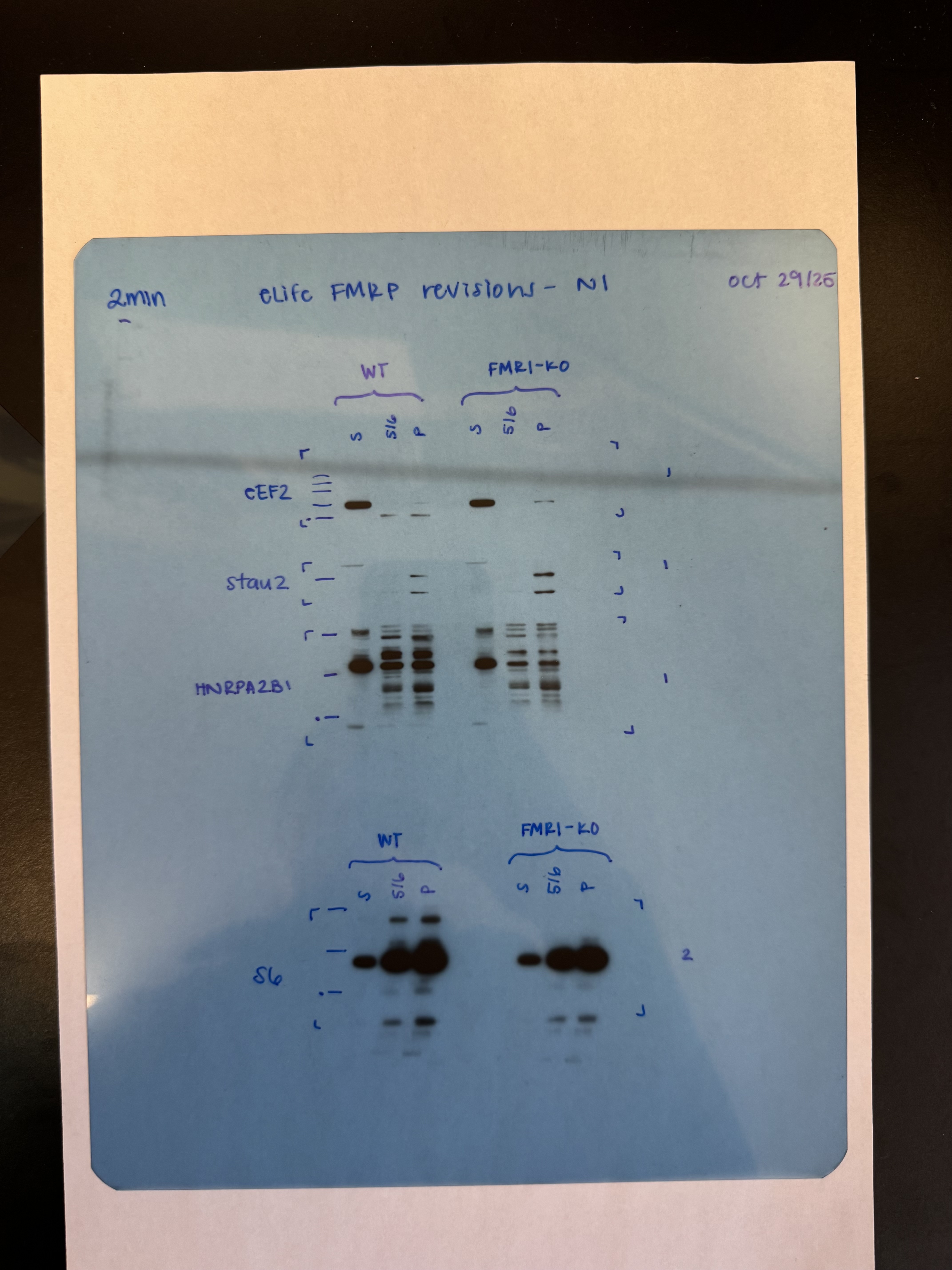

Supplement: Figure 1—source data 2. [file elife-106692-fig1-data2.zip › M2 Oct 29 2min raw.jpeg]

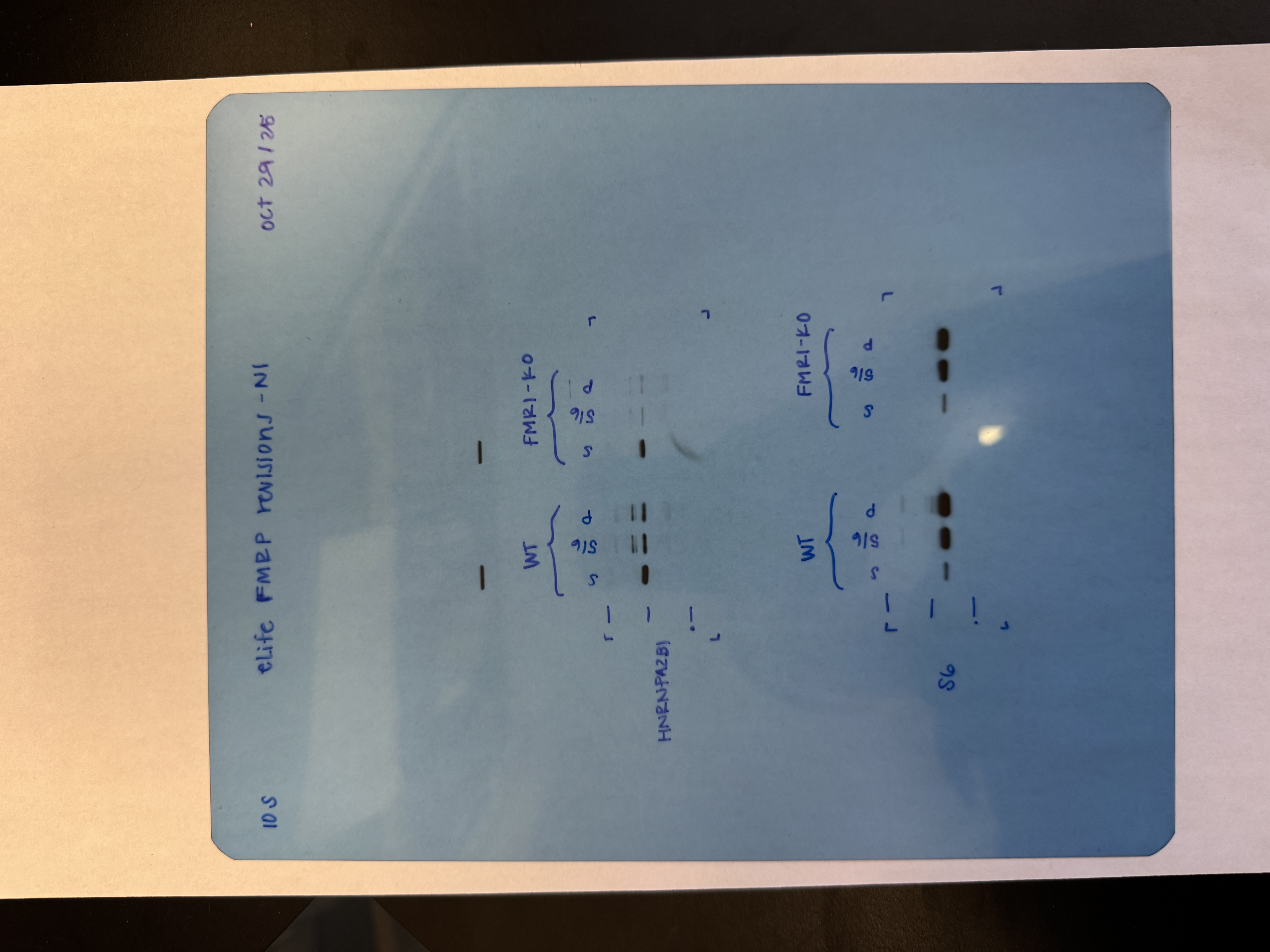

Supplement: Figure 1—source data 2. [file elife-106692-fig1-data2.zip › M2 Oct 29 10s raw.jpeg]

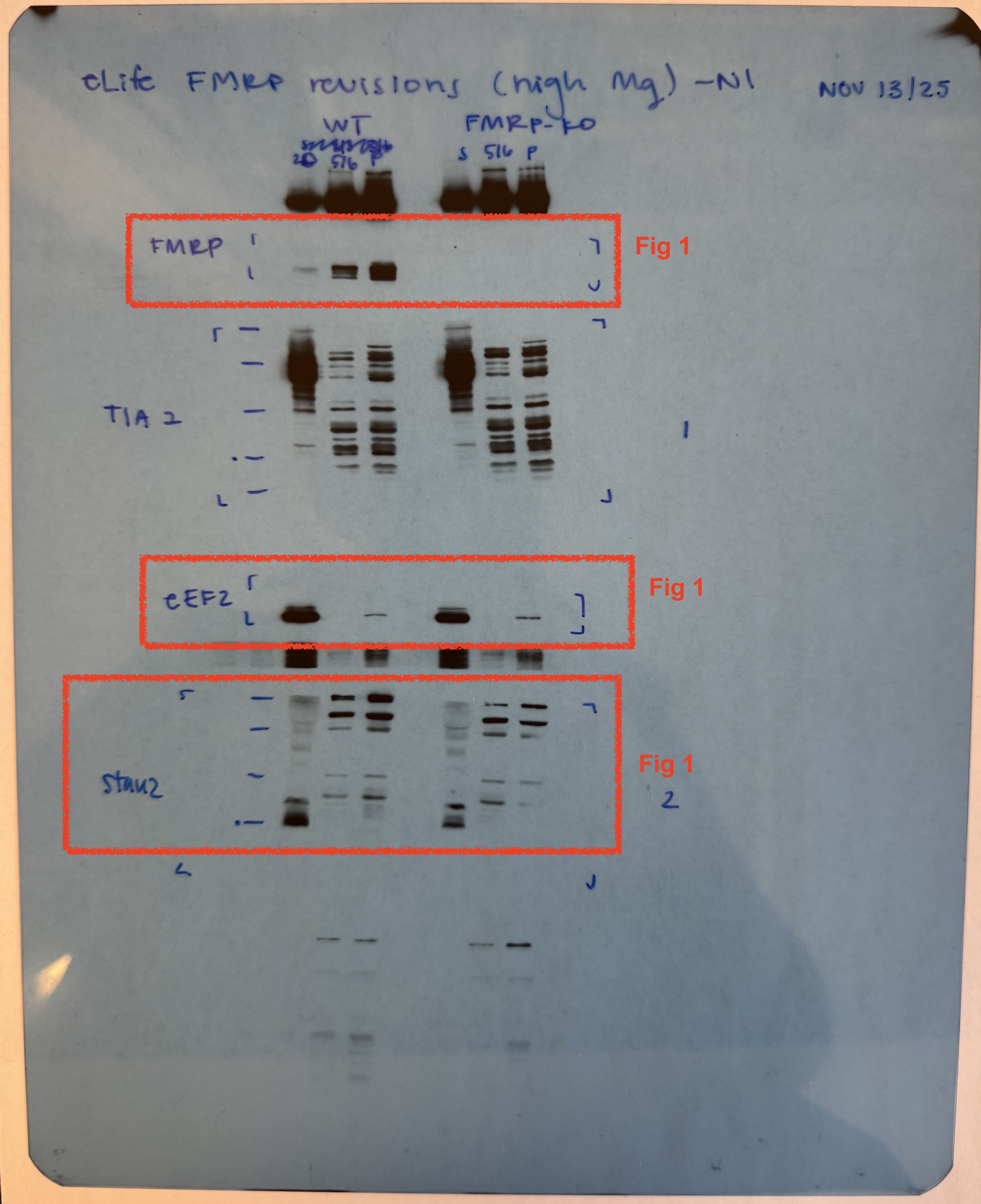

Supplement: Figure 1—source data 2. [file elife-106692-fig1-data2.zip › N1 Nov 13 1 min raw.jpeg]

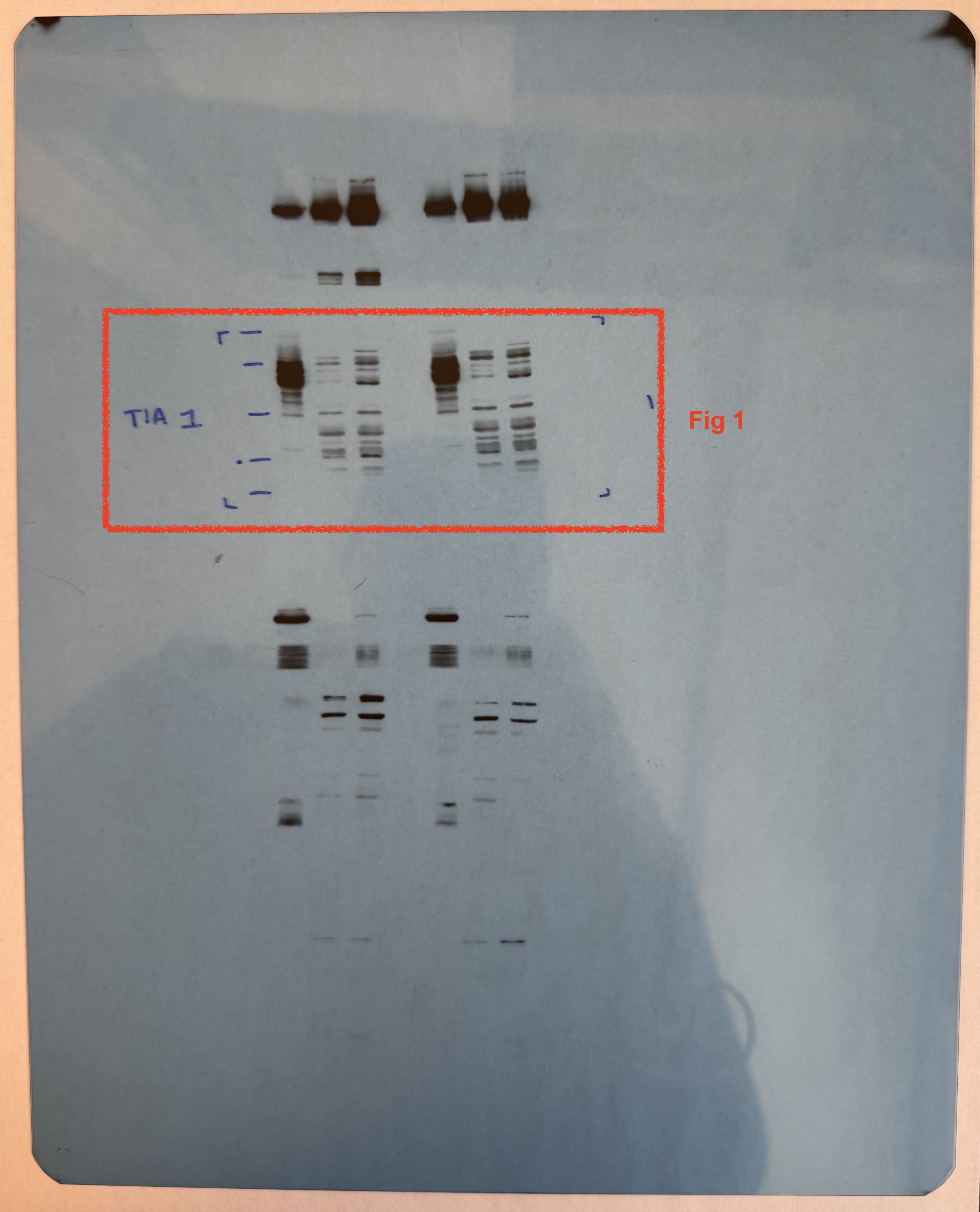

Supplement: Figure 1—source data 2. [file elife-106692-fig1-data2.zip › N1 Nov 13 raw .jpeg]

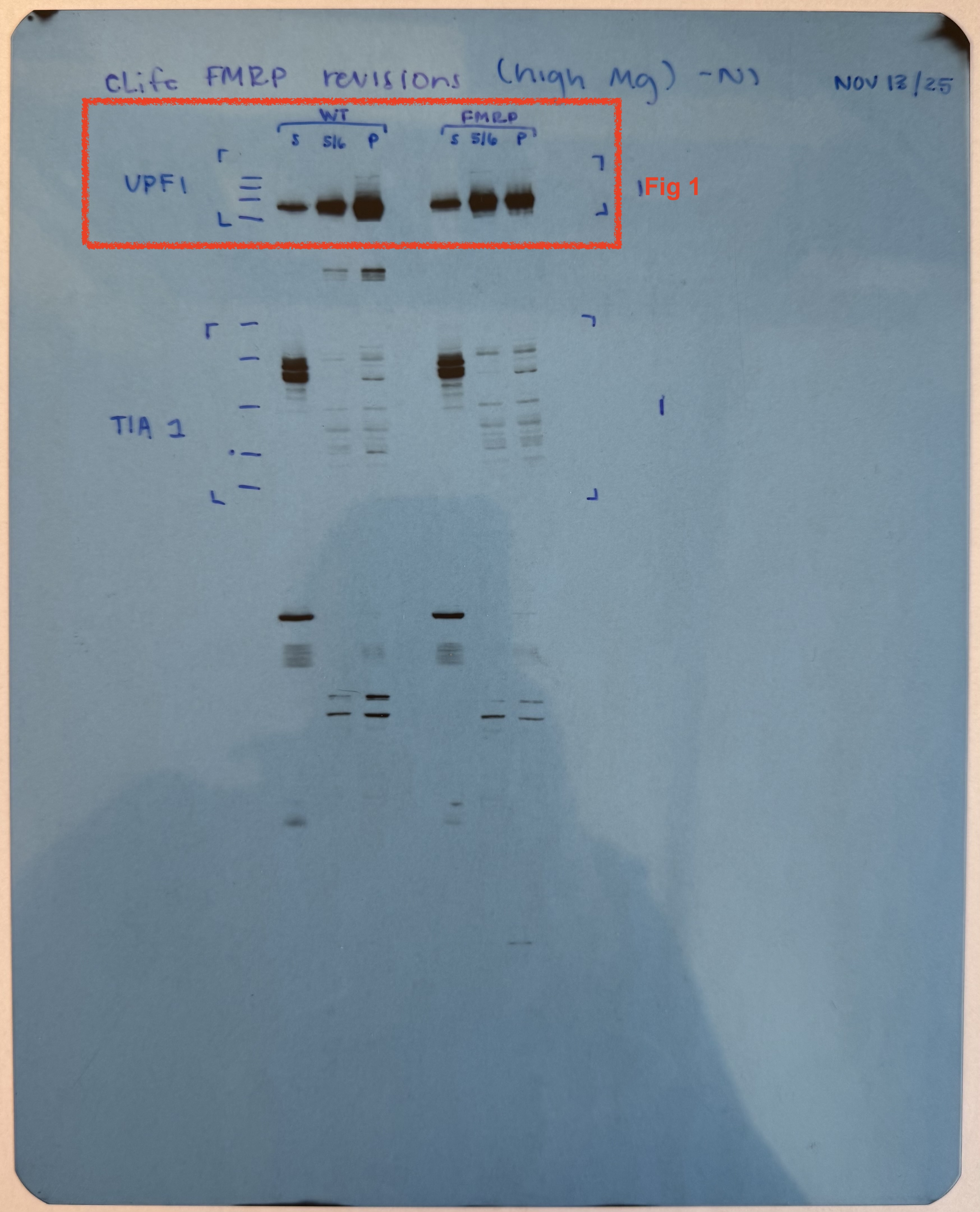

Supplement: Figure 1—source data 2. [file elife-106692-fig1-data2.zip › N1 Nov 13 raw.jpeg]

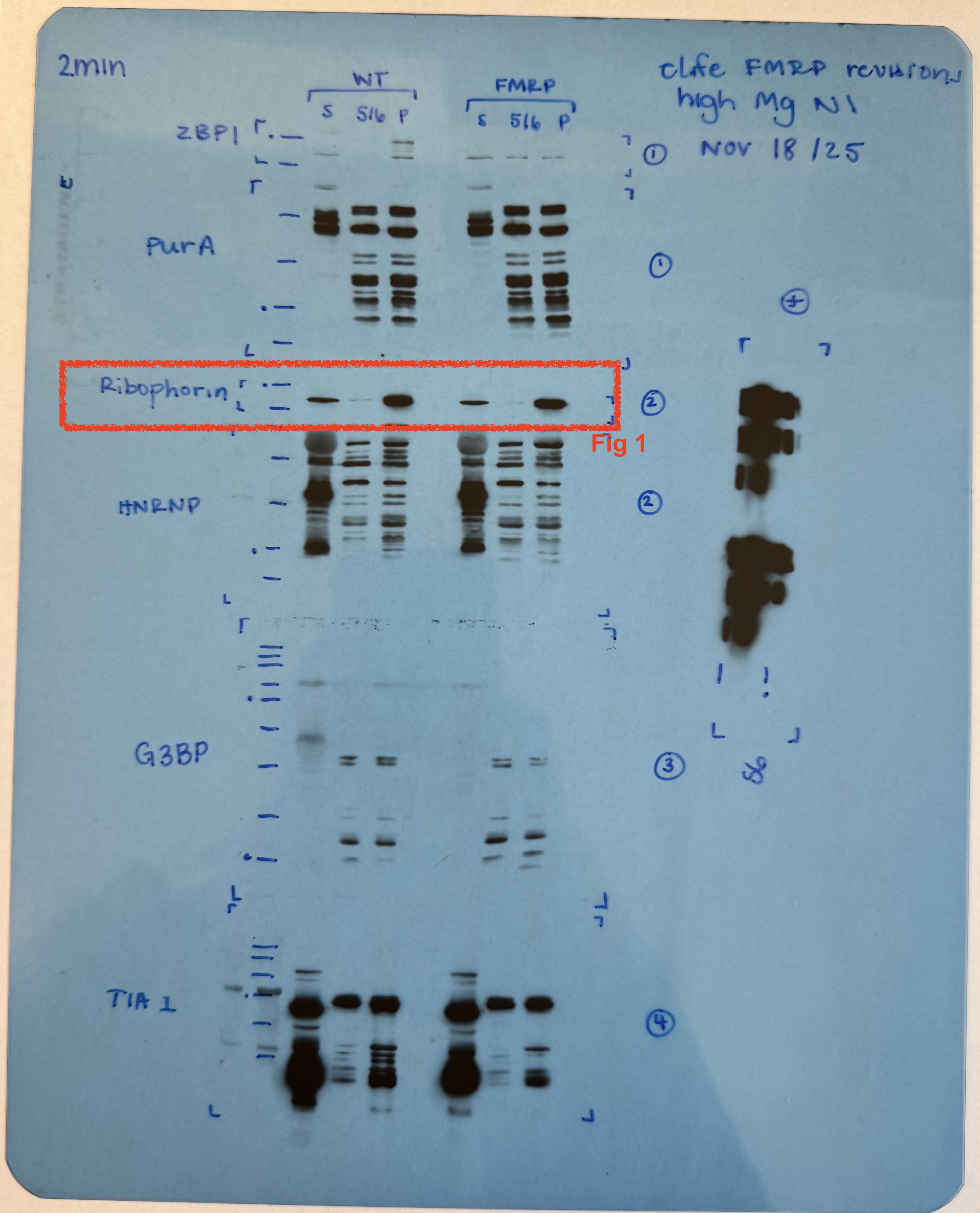

Supplement: Figure 1—source data 2. [file elife-106692-fig1-data2.zip › N1 Nov 18 2 min raw.jpeg]

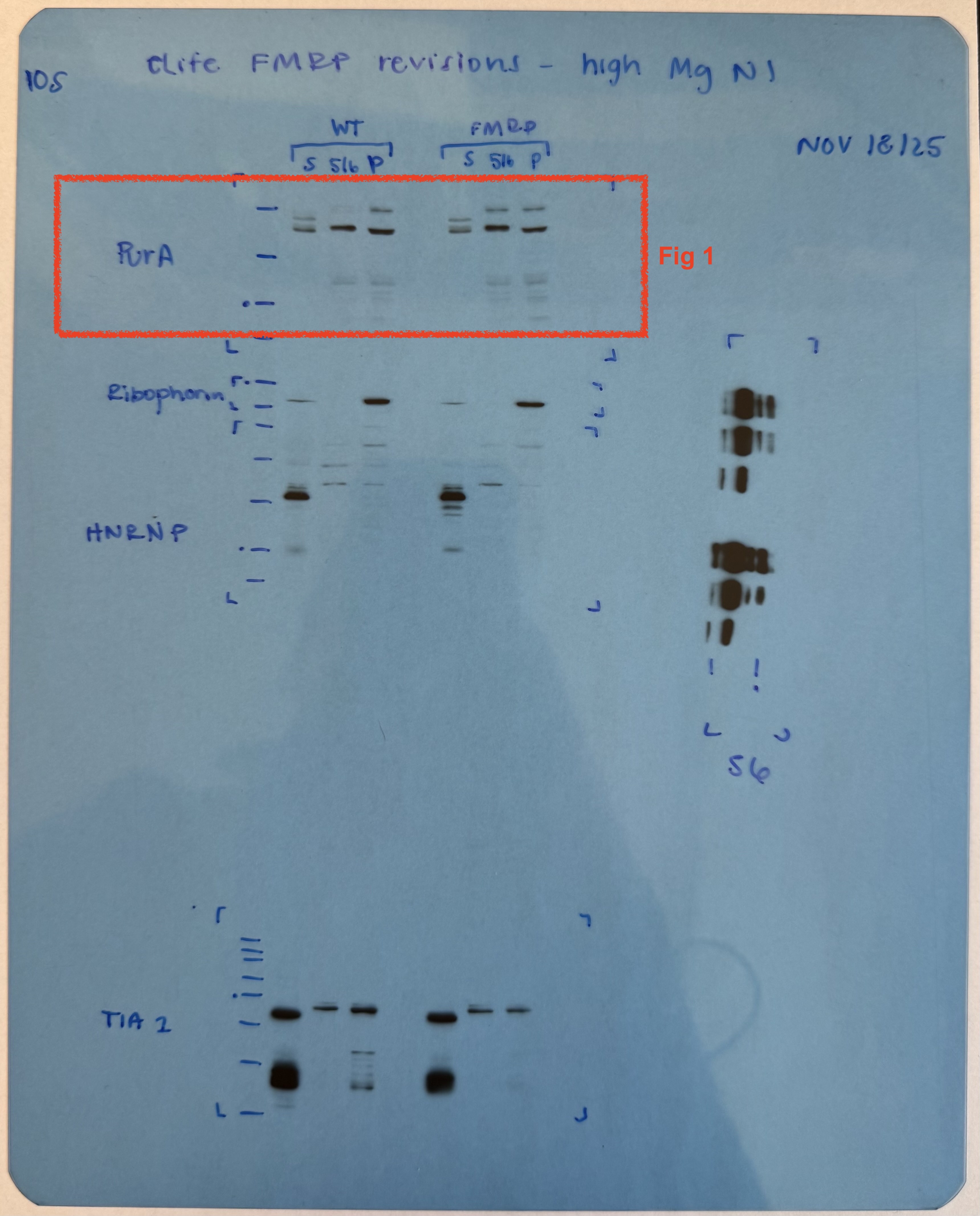

Supplement: Figure 1—source data 2. [file elife-106692-fig1-data2.zip › N1 Nov 18 10s raw.jpeg]

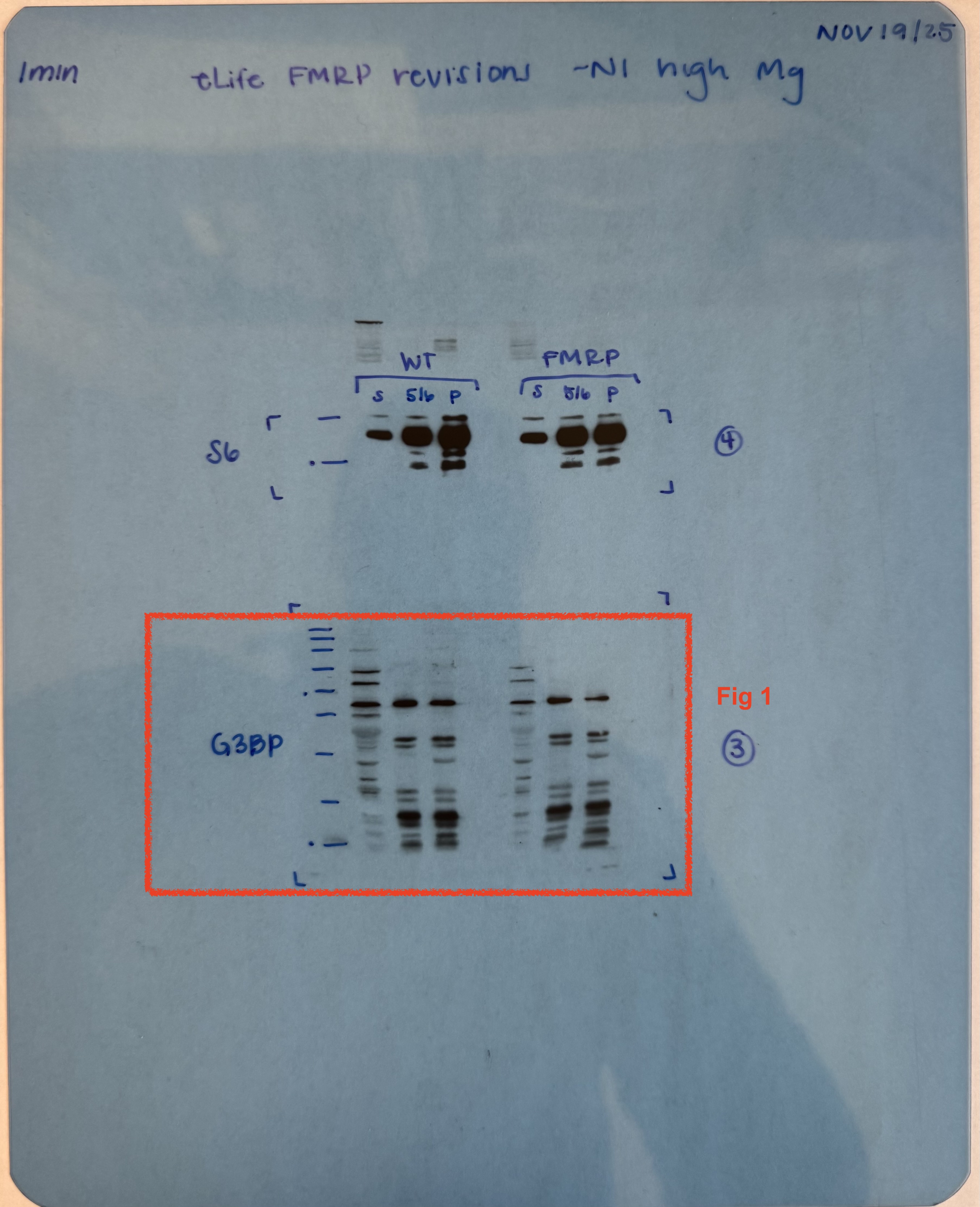

Supplement: Figure 1—source data 2. [file elife-106692-fig1-data2.zip › N1 Nov 19 1 min raw.jpeg]

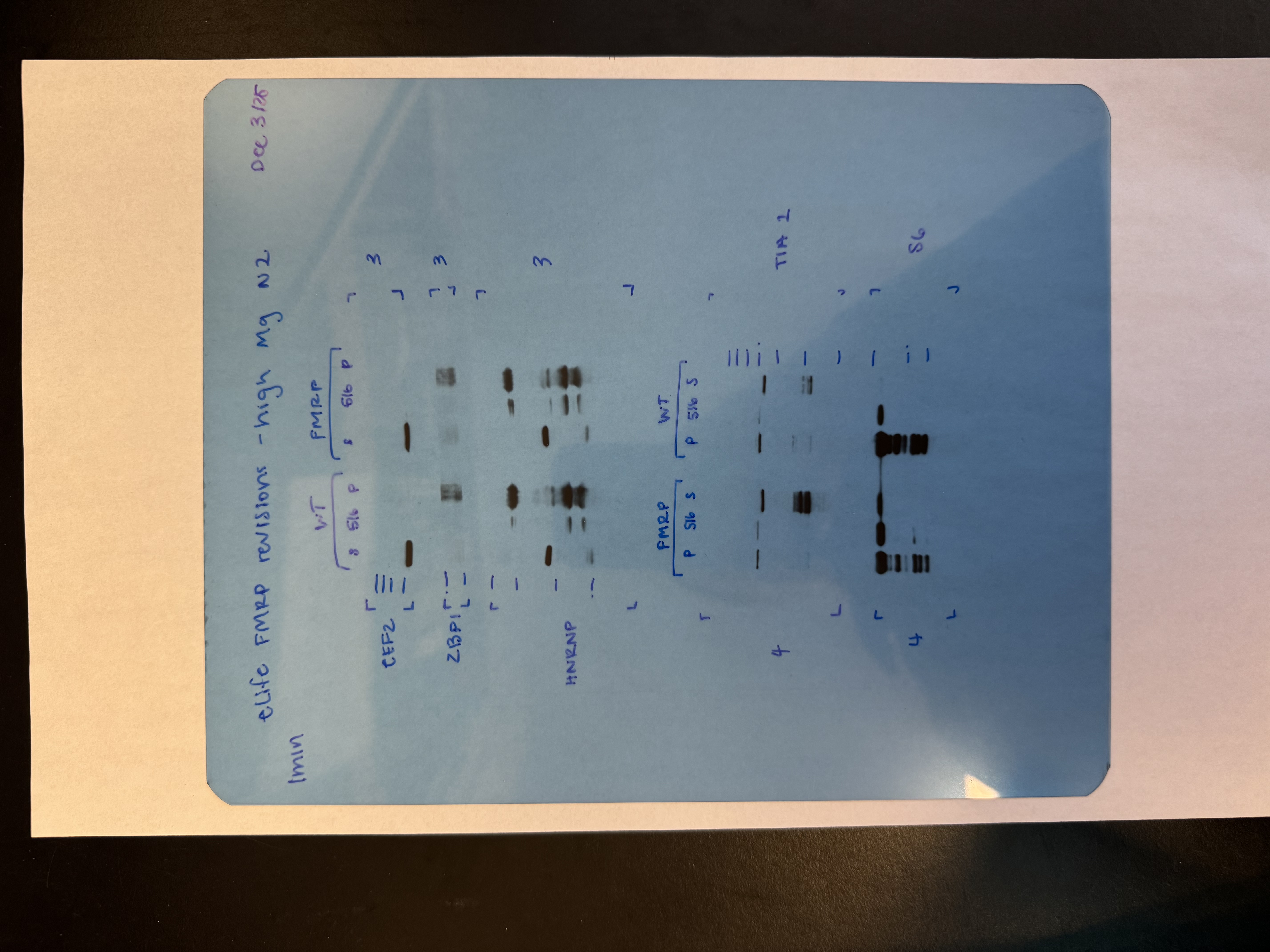

Supplement: Figure 1—source data 2. [file elife-106692-fig1-data2.zip › N2 Dec 3 1 min raw.jpeg]

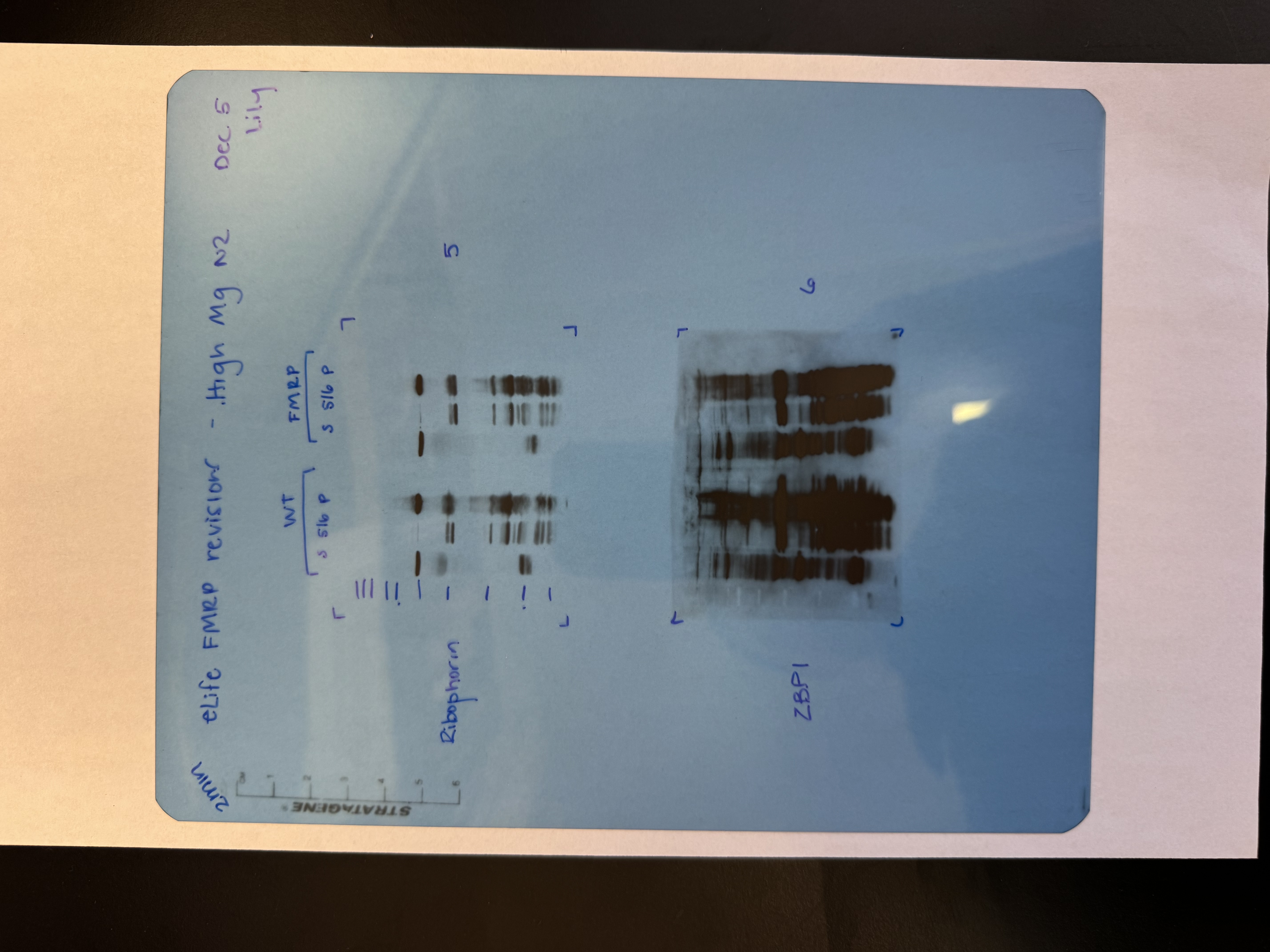

Supplement: Figure 1—source data 2. [file elife-106692-fig1-data2.zip › N2 Dec 5 2 min raw.jpeg]

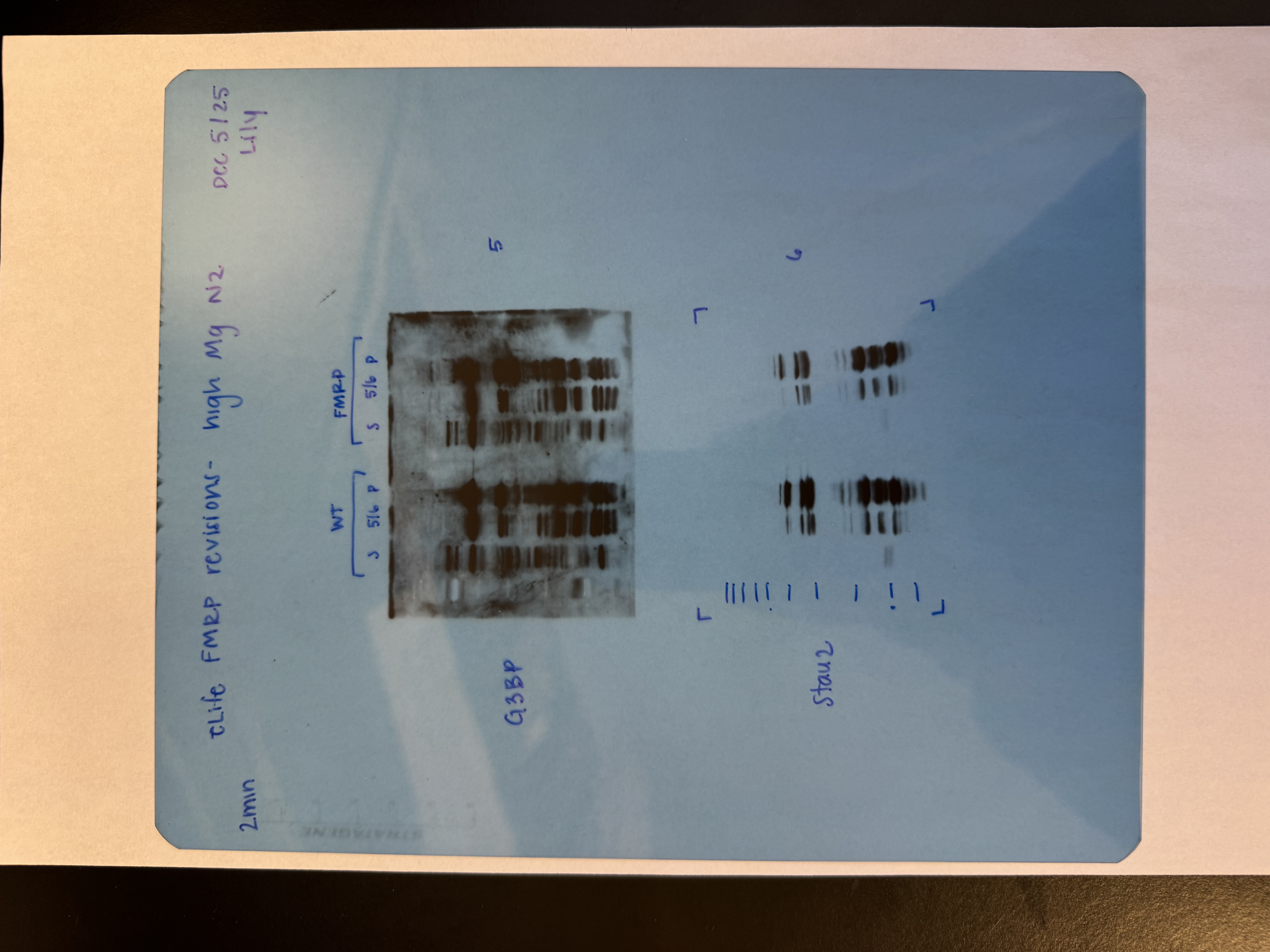

Supplement: Figure 1—source data 2. [file elife-106692-fig1-data2.zip › N2 dec 5 2min raw.jpeg]

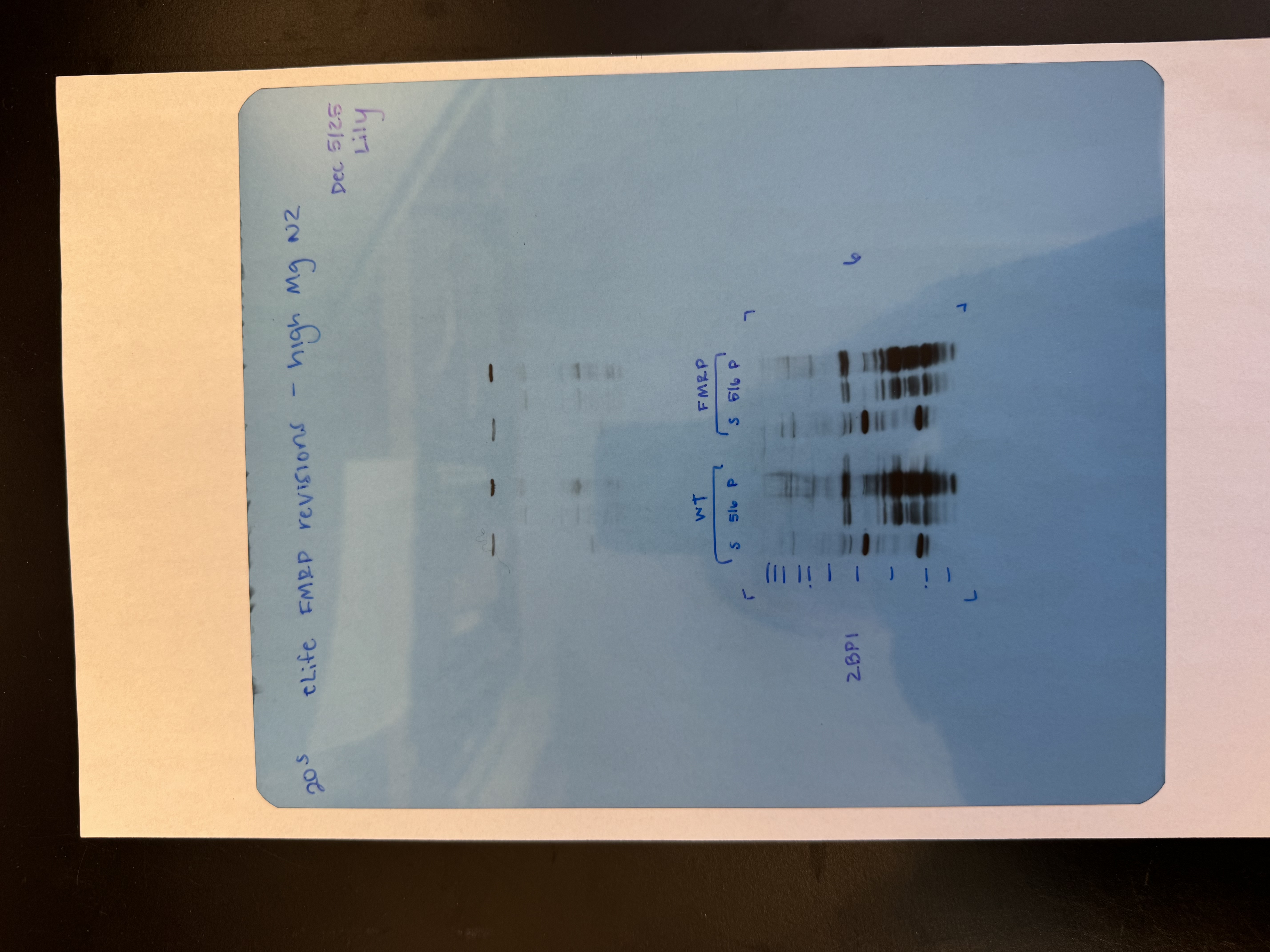

Supplement: Figure 1—source data 2. [file elife-106692-fig1-data2.zip › N2 Dec 5 20s raw.jpeg]

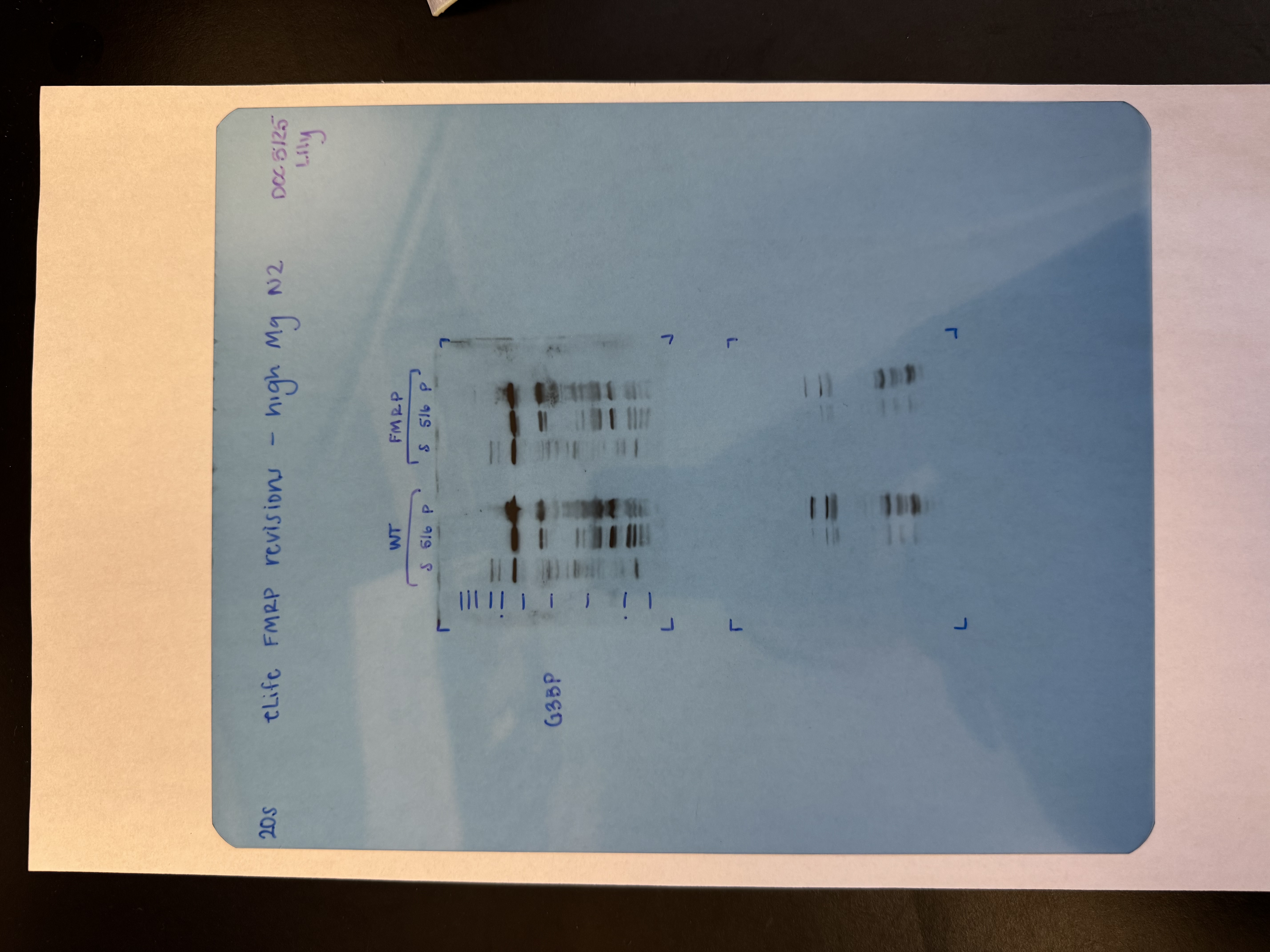

Supplement: Figure 1—source data 2. [file elife-106692-fig1-data2.zip › N2 Dec 6 20s raw.jpeg]

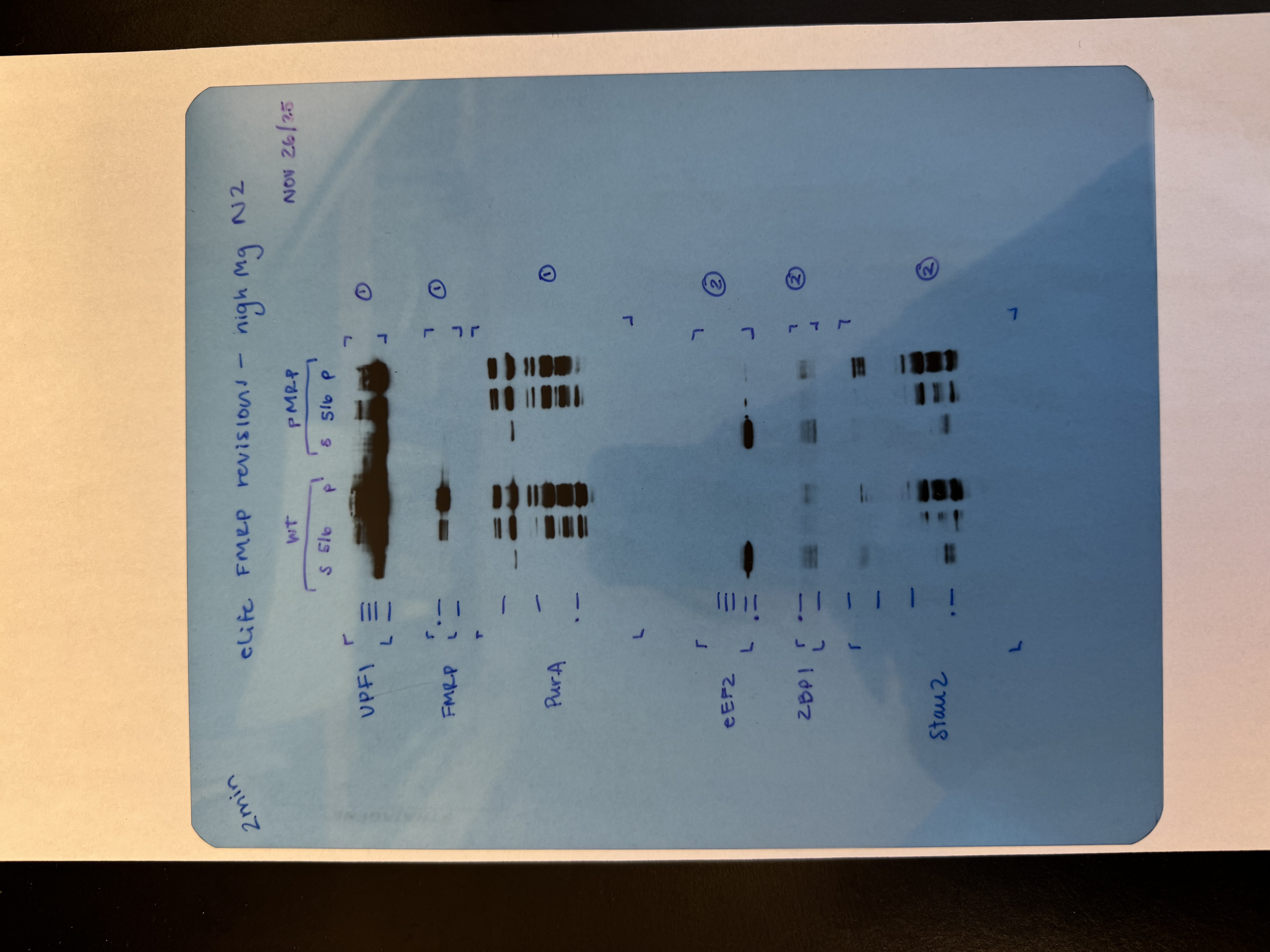

Supplement: Figure 1—source data 2. [file elife-106692-fig1-data2.zip › N2 Nov 26 2 min raw.jpeg]

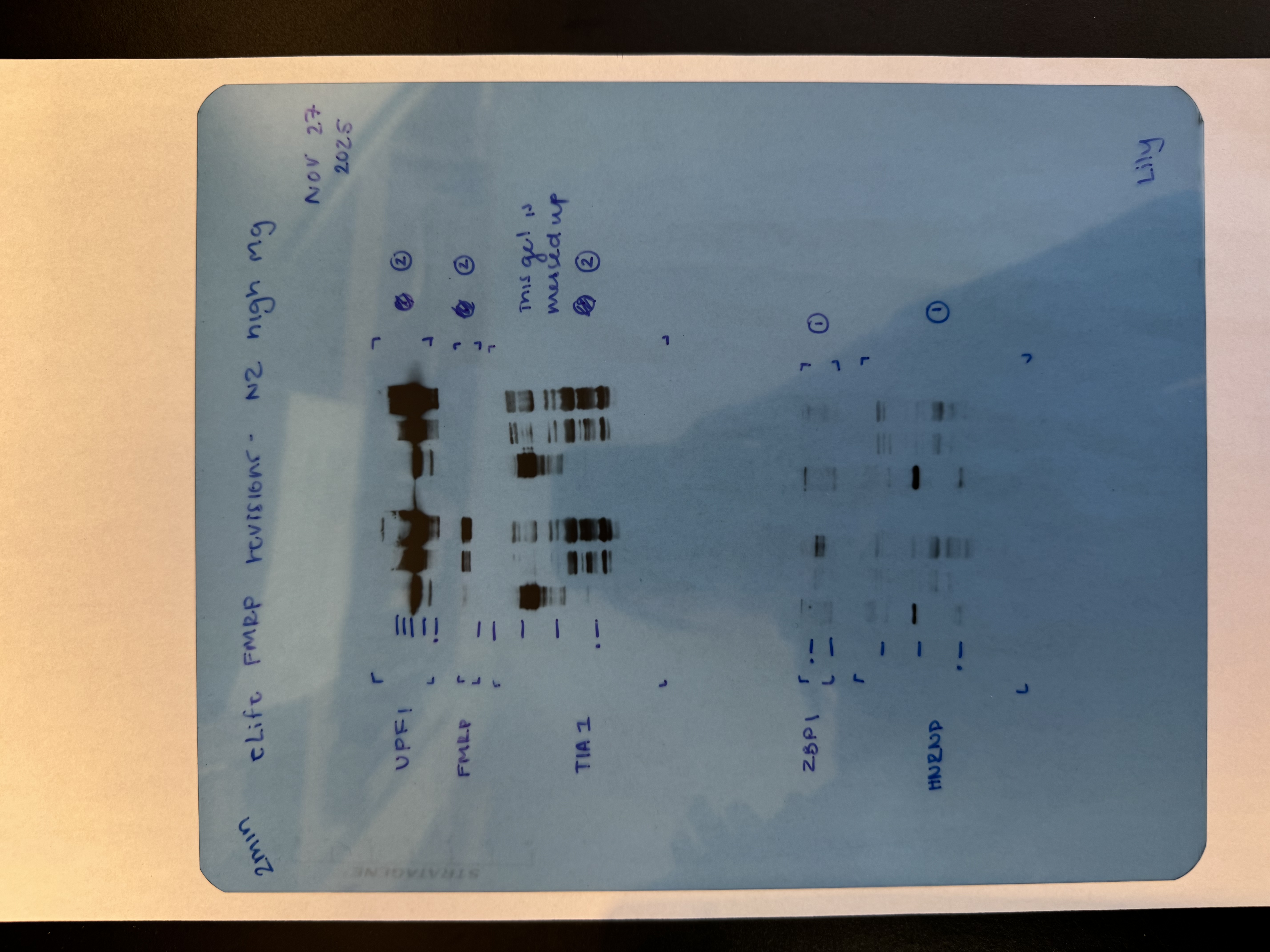

Supplement: Figure 1—source data 2. [file elife-106692-fig1-data2.zip › N2 nov 27 2 min raw.jpeg]

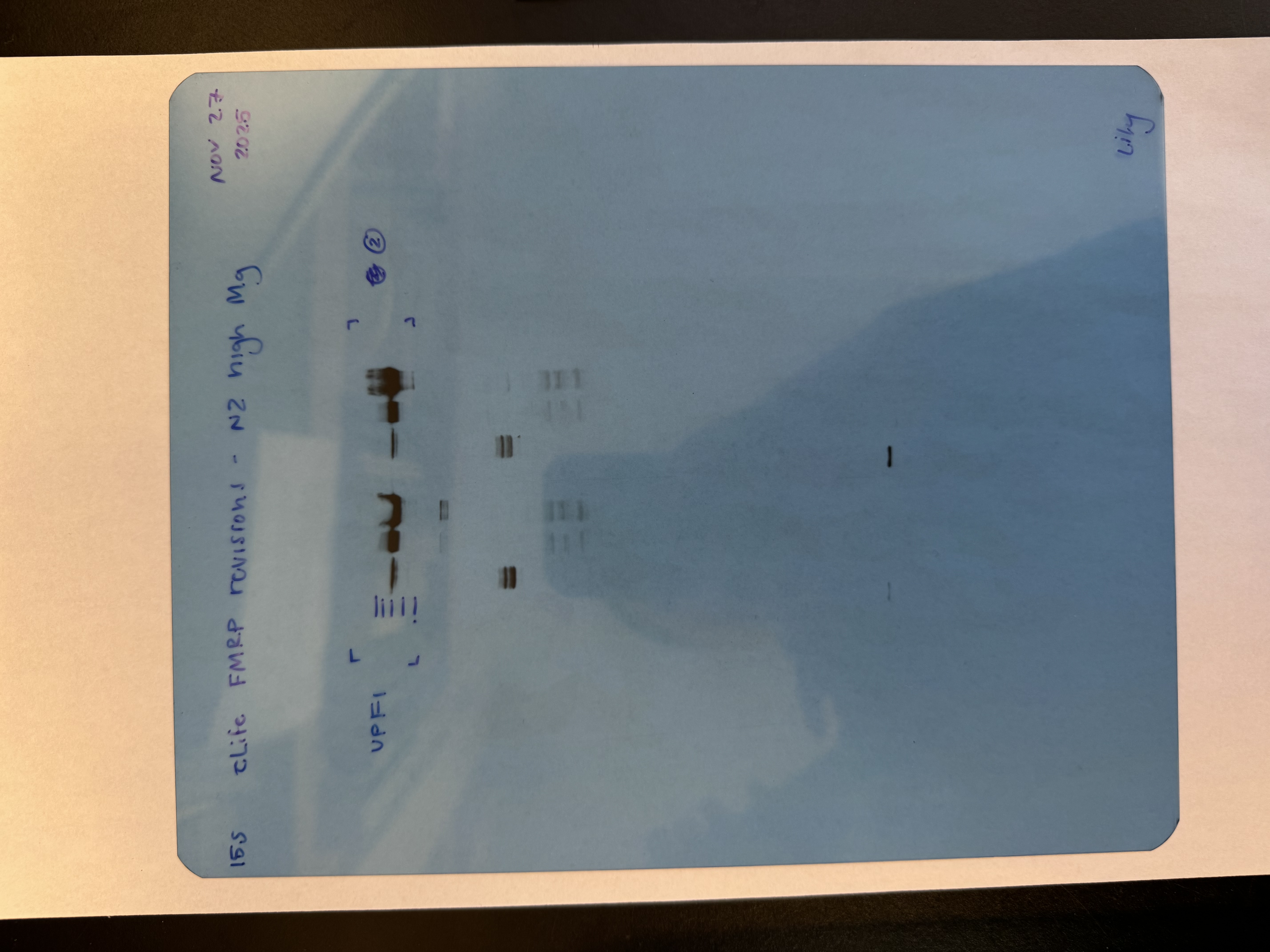

Supplement: Figure 1—source data 2. [file elife-106692-fig1-data2.zip › N2 nov 27 15s raw.jpeg]

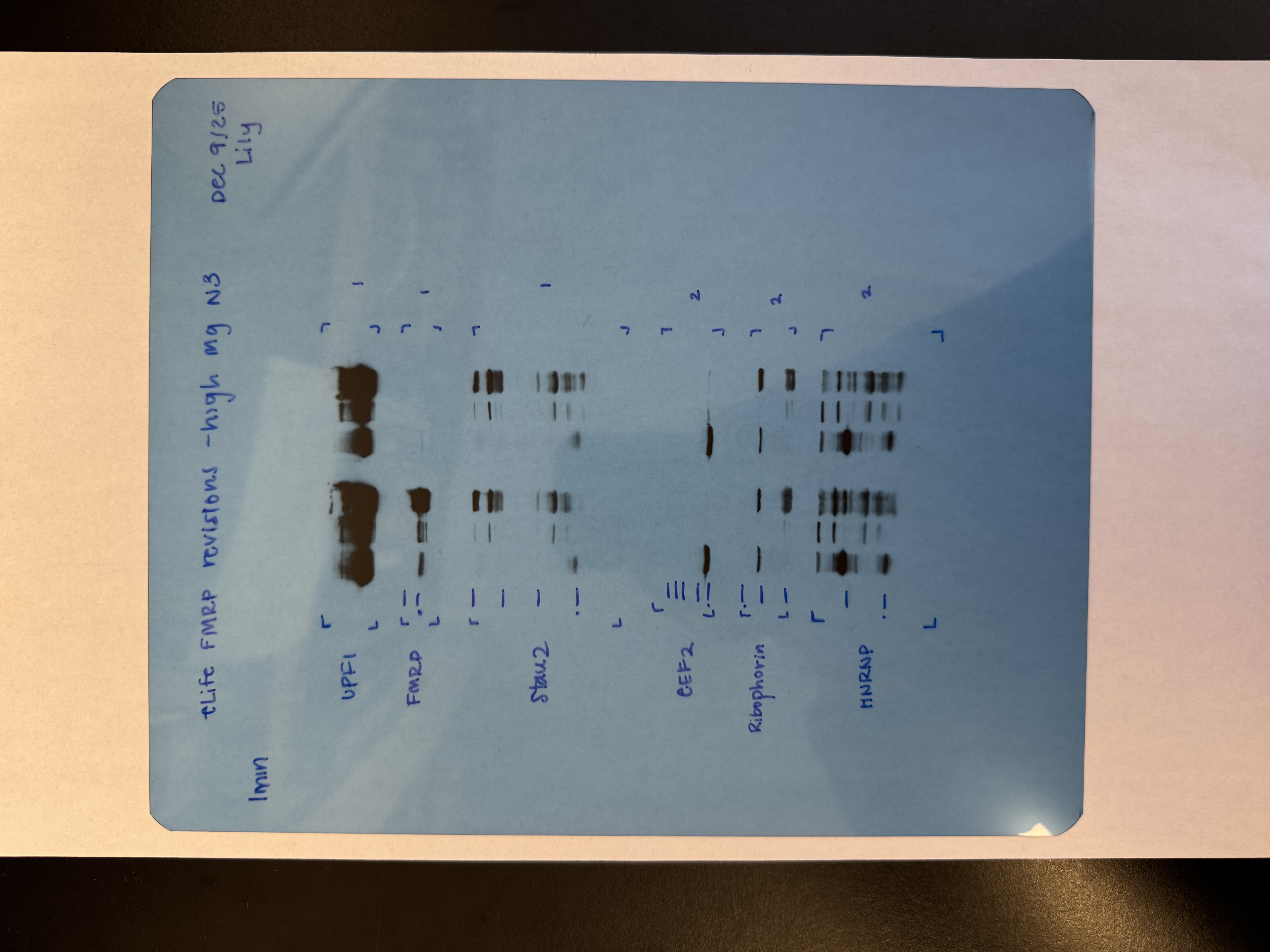

Supplement: Figure 1—source data 2. [file elife-106692-fig1-data2.zip › N3 Dec 9 1 min raw.jpeg]

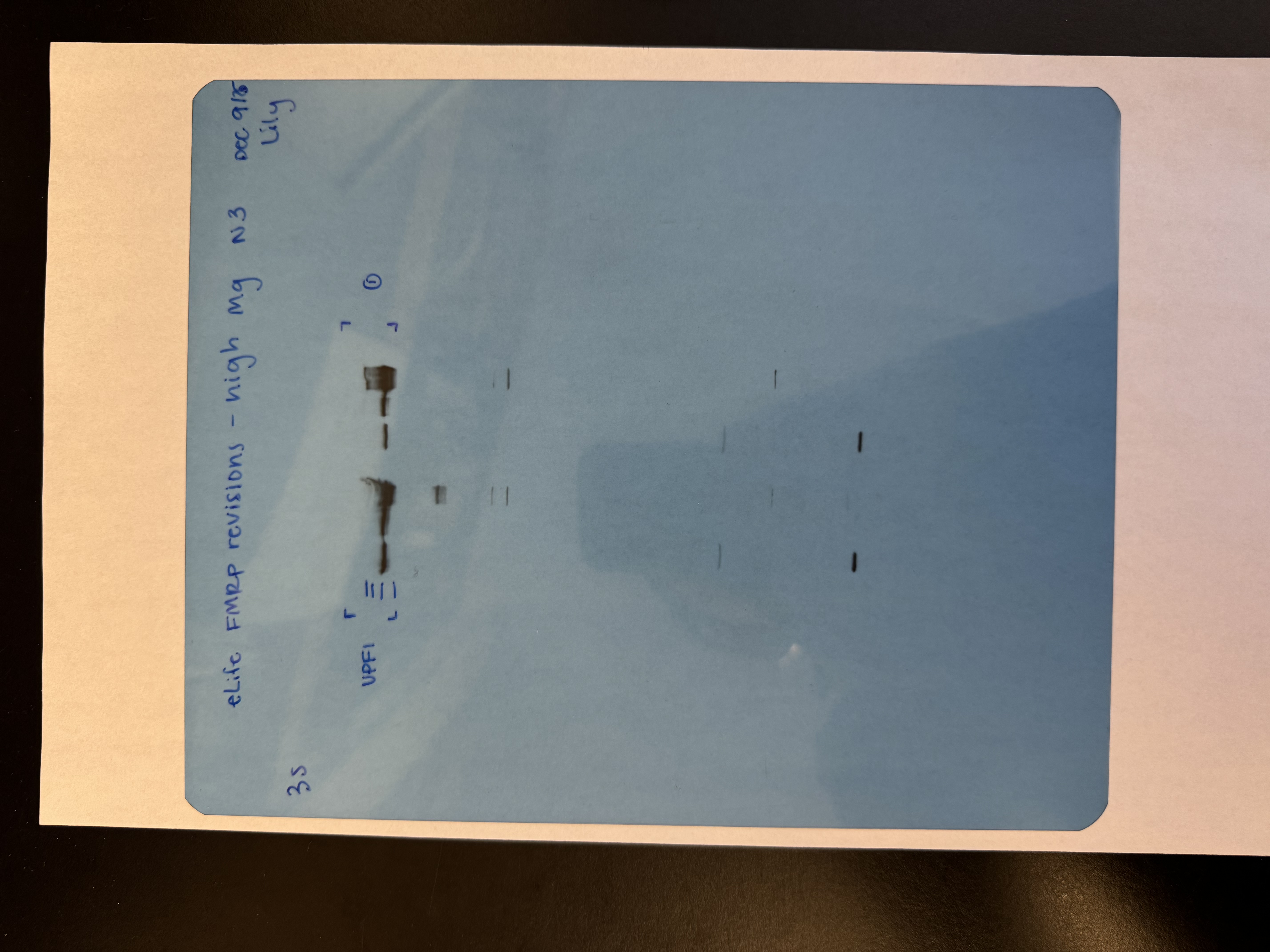

Supplement: Figure 1—source data 2. [file elife-106692-fig1-data2.zip › N3 dec 9 3s raw.jpeg]

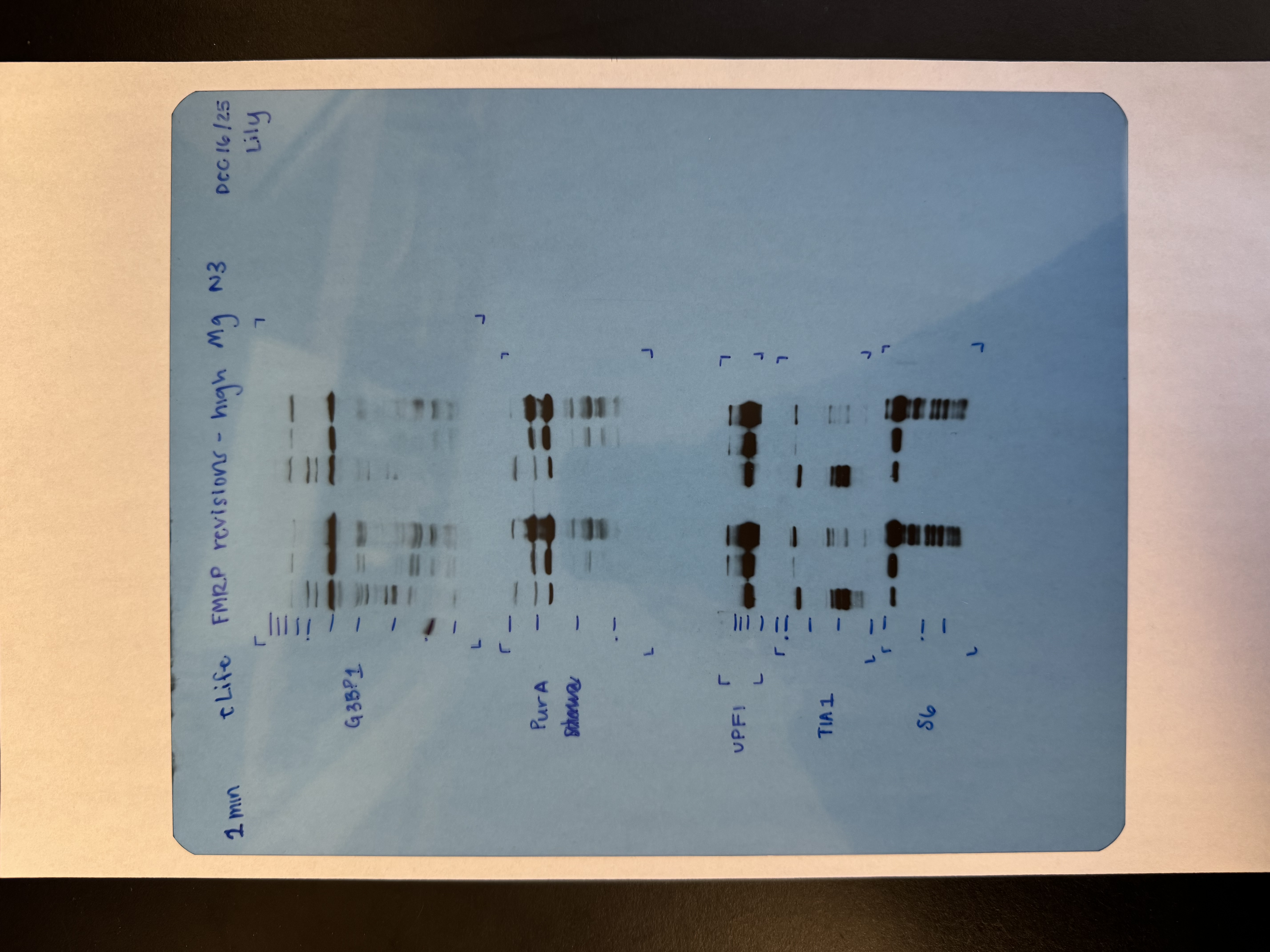

Supplement: Figure 1—source data 2. [file elife-106692-fig1-data2.zip › N3 dec 16 raw .jpeg]

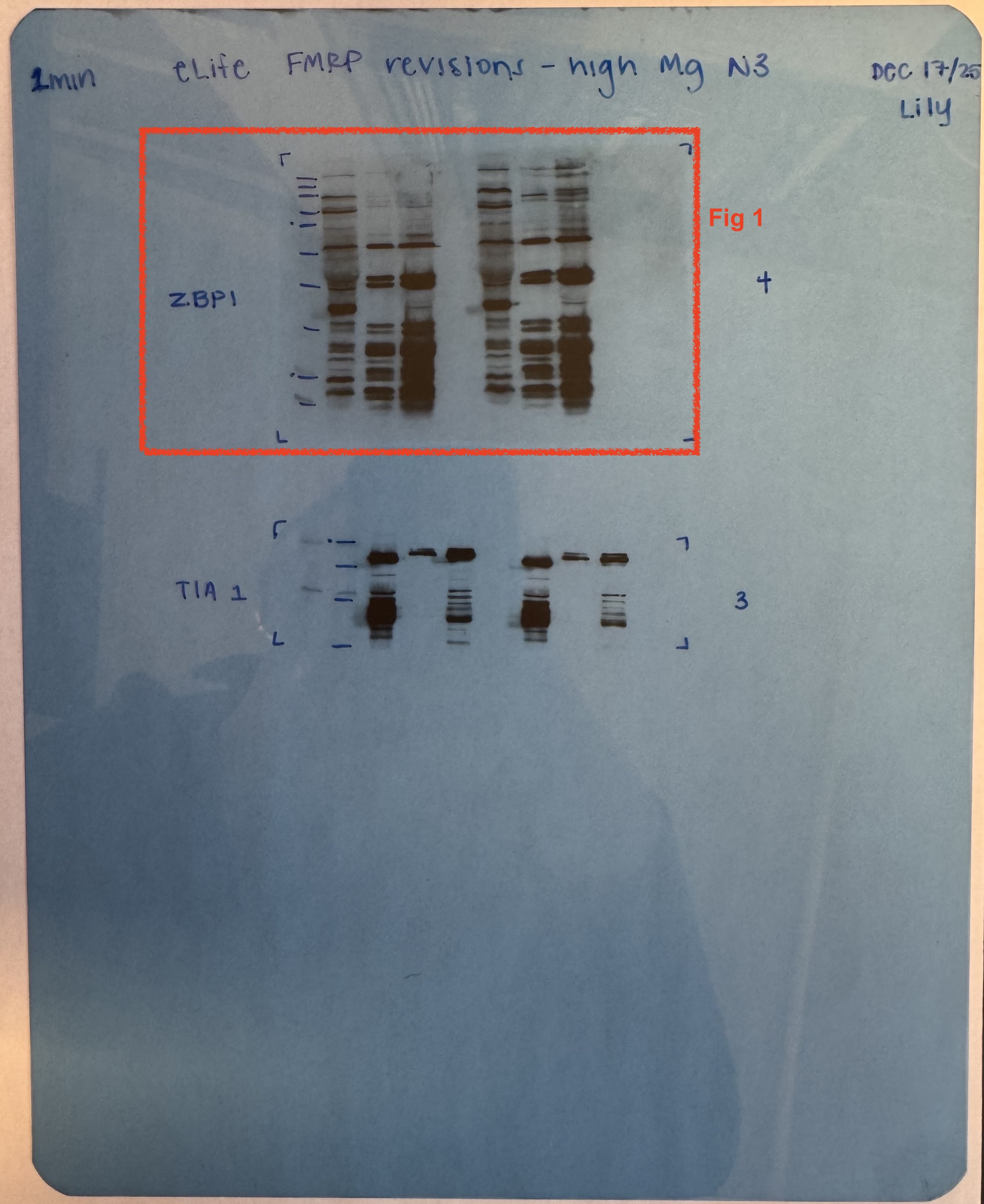

Supplement: Figure 1—source data 2. [file elife-106692-fig1-data2.zip › N3 Dec 17 2 min raw.jpeg]

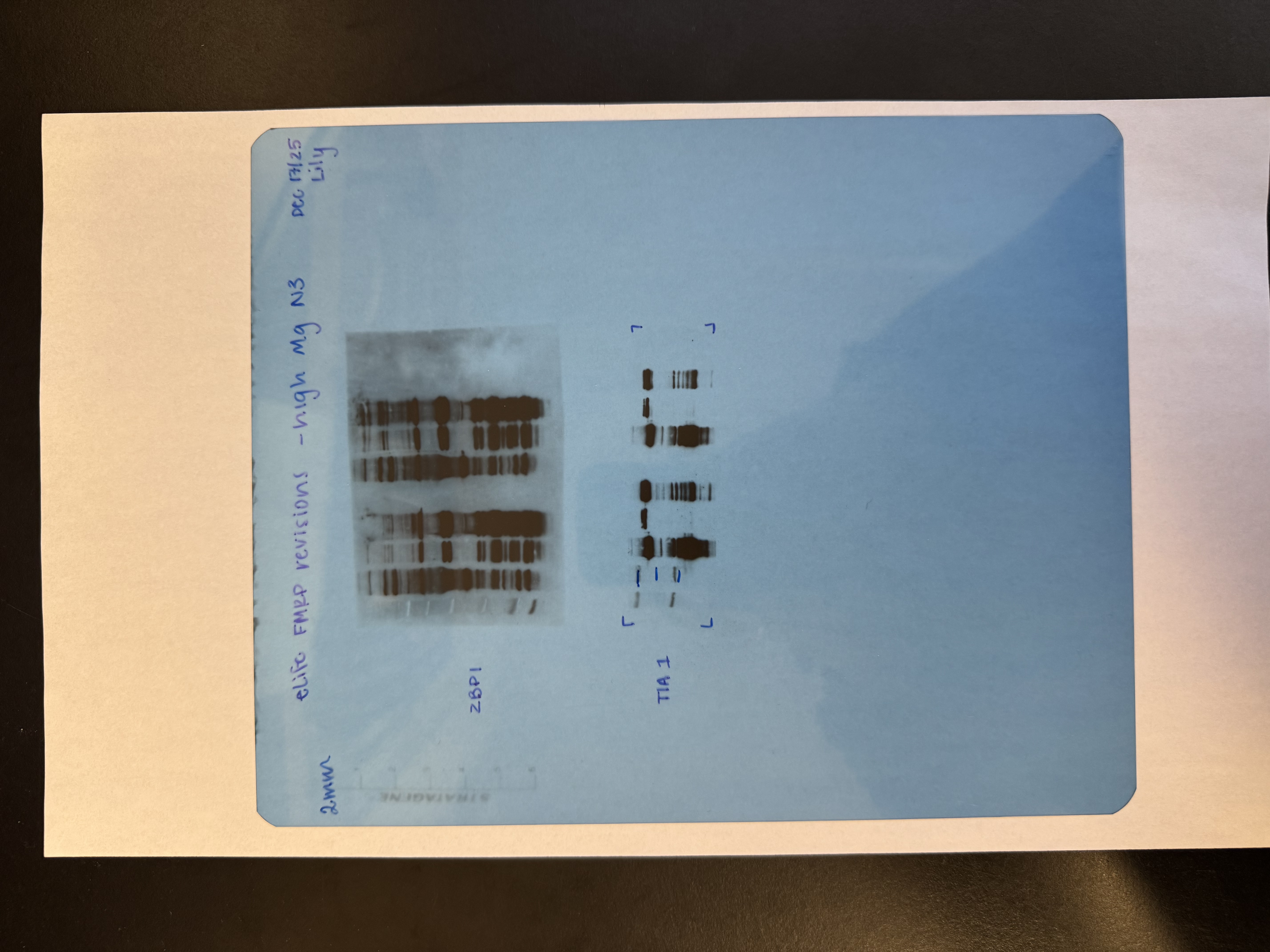

Supplement: Figure 1—source data 2. [file elife-106692-fig1-data2.zip › N3 dec 17 raw.jpeg]

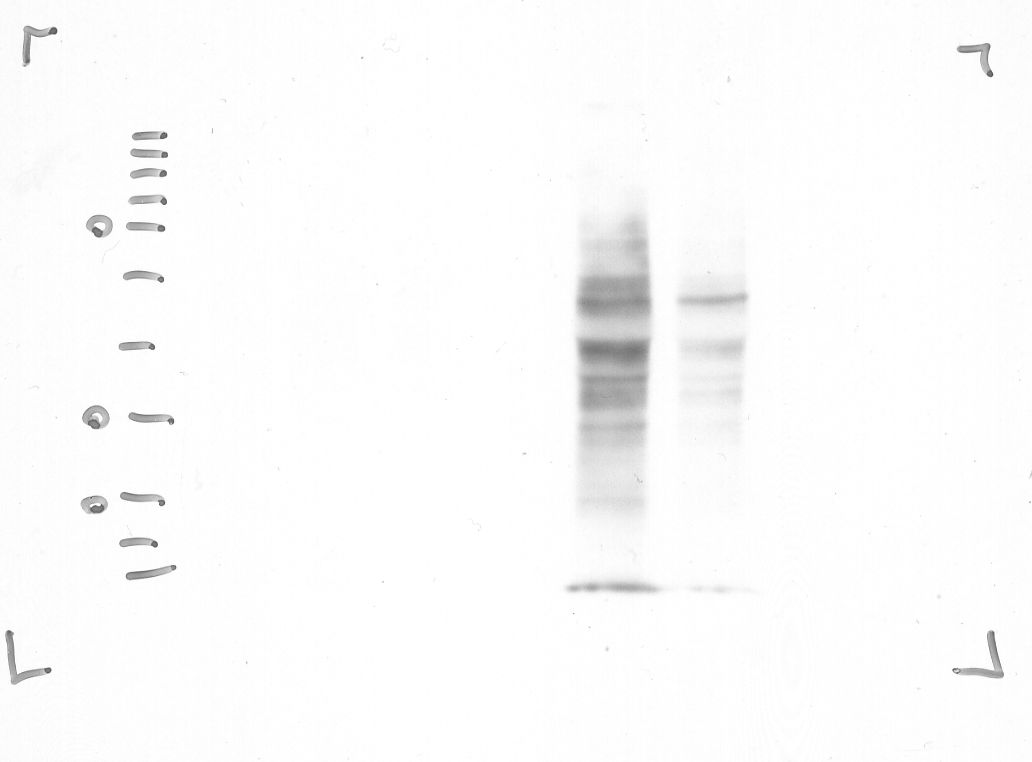

Supplement: Figure 2—source data 2. [file elife-106692-fig2-data2.zip › Figure 2 Source Data 2/Jan 23 23 anti puro V Pellet C57 FMR1 5sec069.tif]

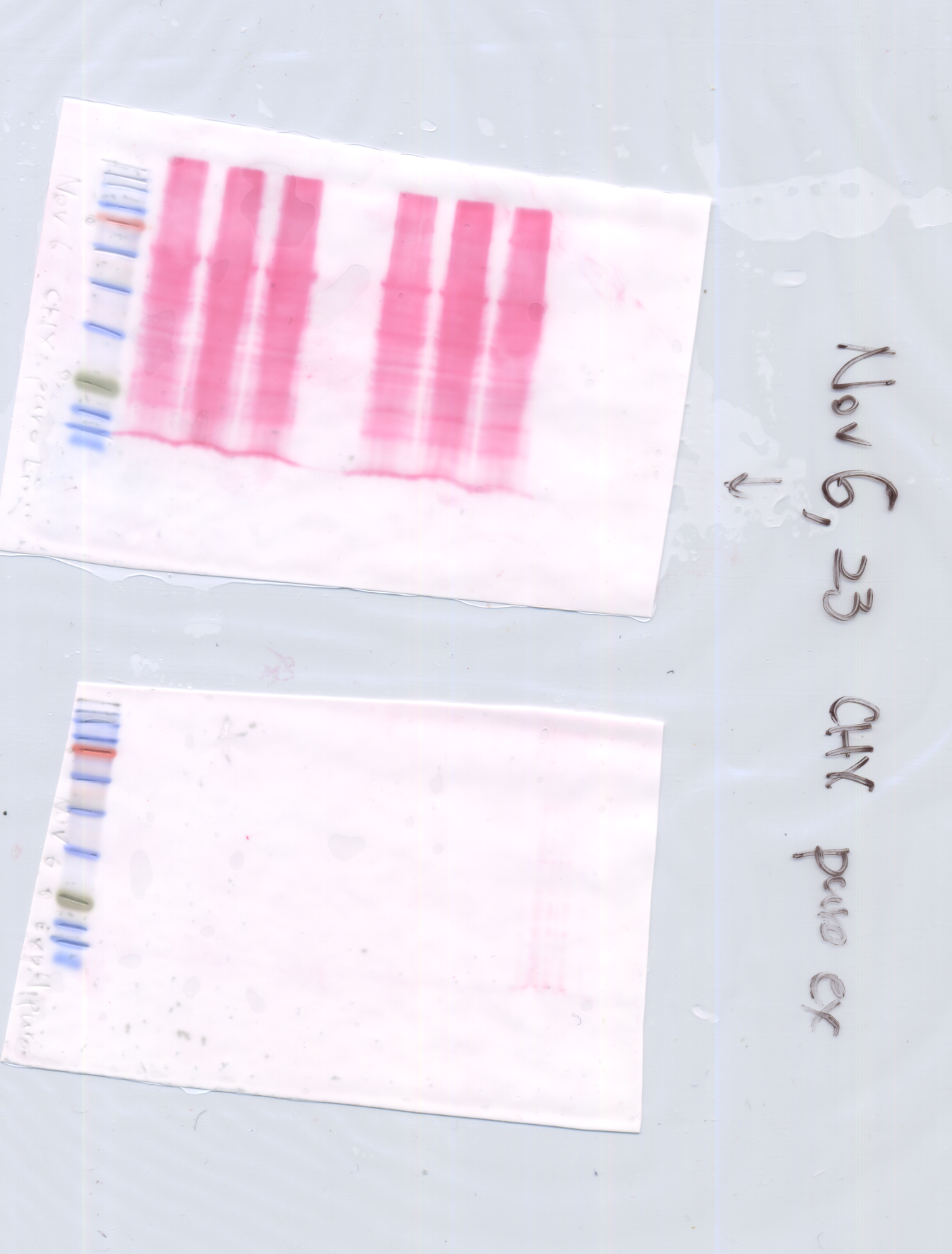

Supplement: Figure 2—source data 2. [file elife-106692-fig2-data2.zip › Figure 2 Source Data 2/Nov 7 CHX puromycylation liver exp 1 + Exp 4 puro- resedimentation redo.tiff]

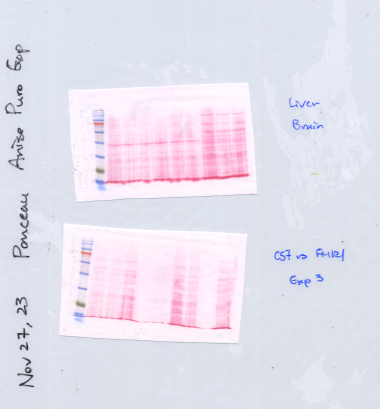

Supplement: Figure 2—source data 2. [file elife-106692-fig2-data2.zip › Figure 2 Source Data 2/Nov 27 23 Ponceau Liver Brain exp2 C57 vs FMR1 Exp 3.tiff]

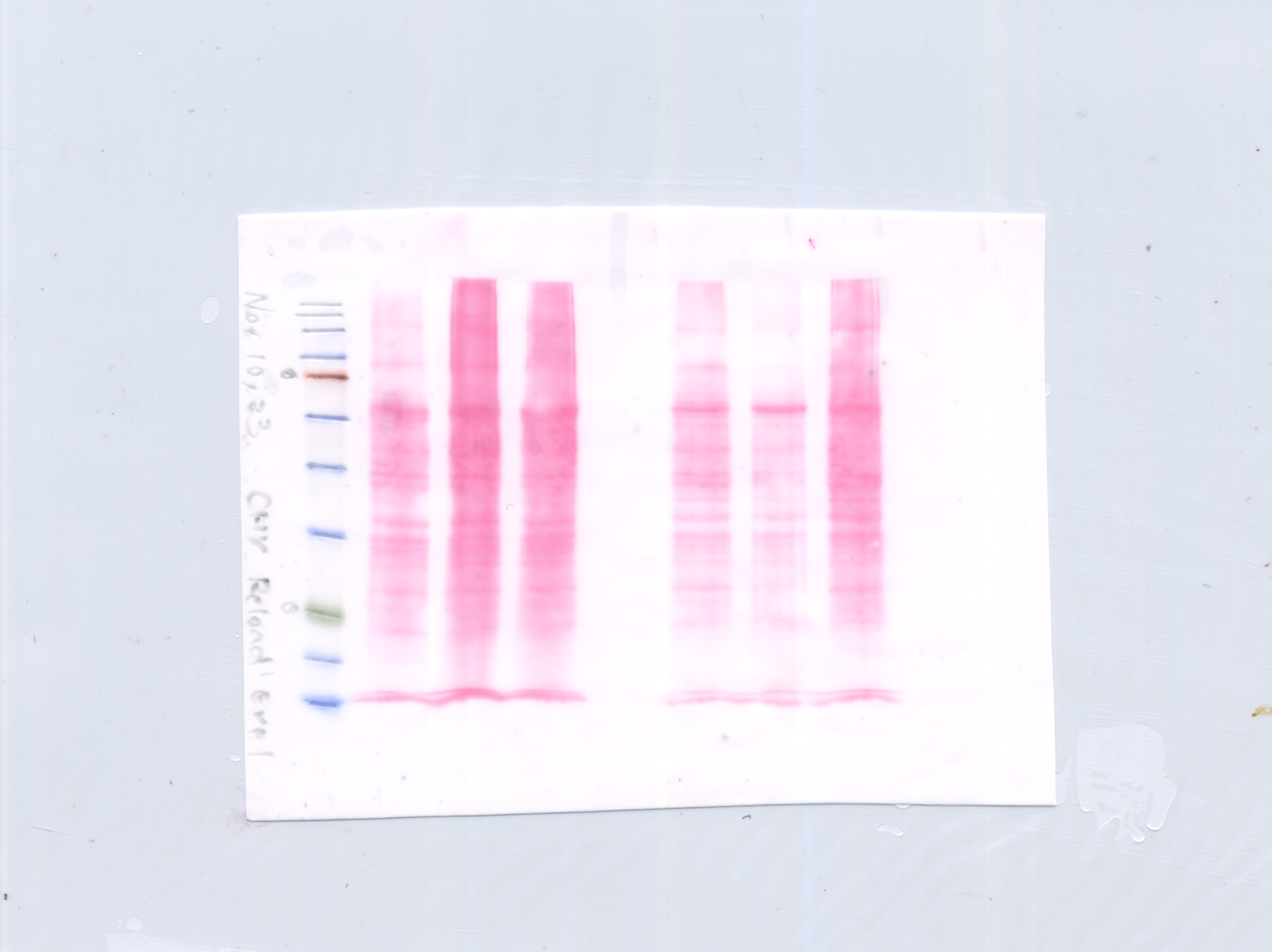

Supplement: Figure 2—source data 2. [file elife-106692-fig2-data2.zip › Figure 2 Source Data 2/Nov 10 23 Ponceau cycloheximide puro aniso comp Exp 1 15ul .tiff]

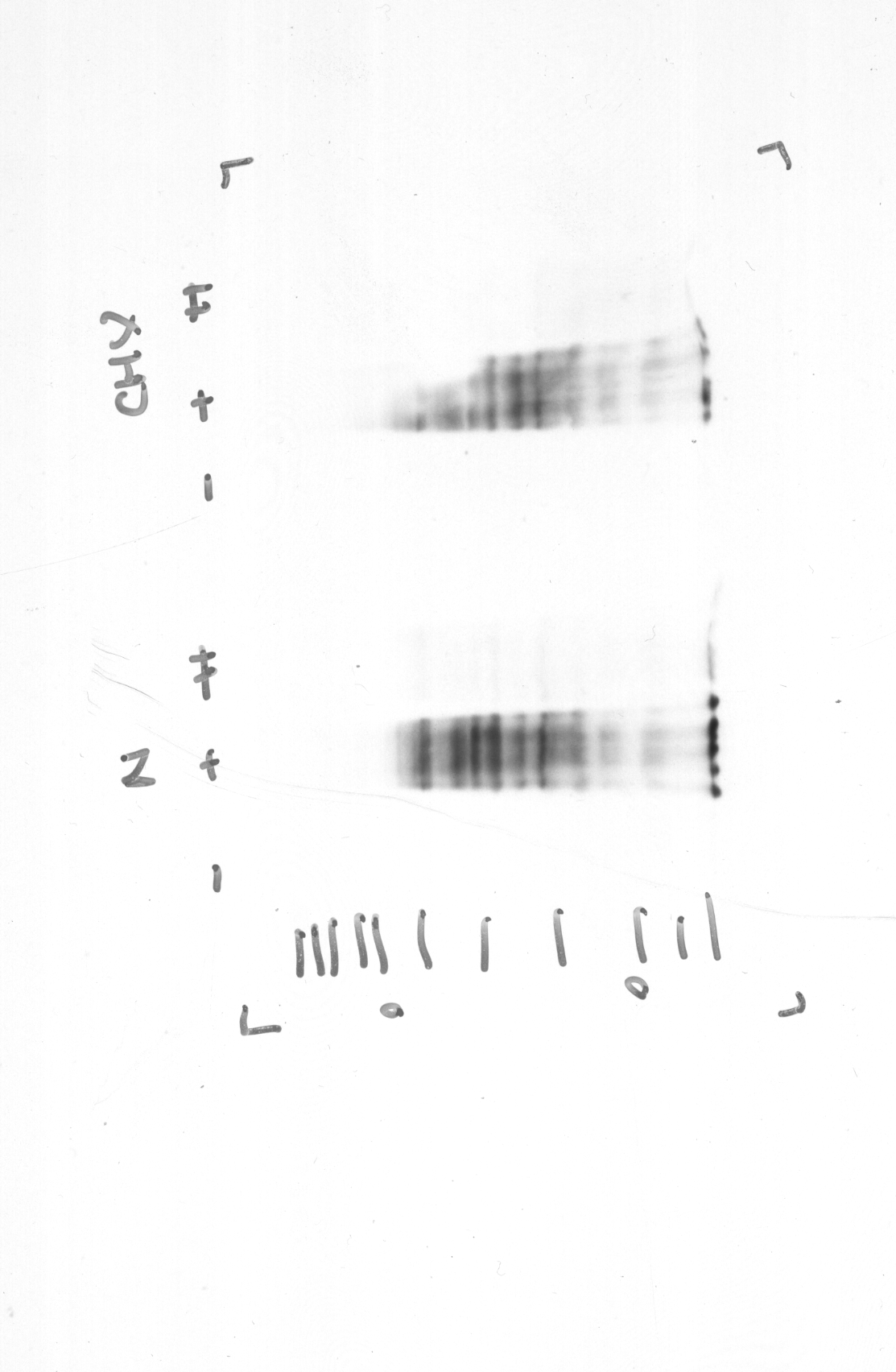

Supplement: Figure 2—source data 2. [file elife-106692-fig2-data2.zip › Figure 2 Source Data 2/Nov 14 anti puro Liver CHX Puro Aniso Comp Exp 2 45sec .tiff]

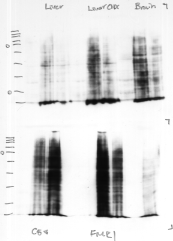

Supplement: Figure 2—source data 2. [file elife-106692-fig2-data2.zip › Figure 2 Source Data 2/Nov 30 anti puro Liver Brain exp2 & C57 FMR1 Exp3 5min.tiff]

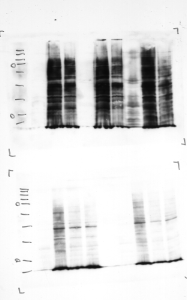

Supplement: Figure 2—source data 2. [file elife-106692-fig2-data2.zip › Figure 2 Source Data 2/Dec 07 Puro Aniso Comp C57 FMR1 Polyribosome Liver 45sec .tiff]

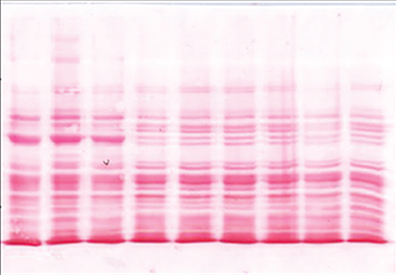

Supplement: Figure 2—source data 2. [file elife-106692-fig2-data2.zip › Figure 2 Source Data 2/Ponceau2.tif]

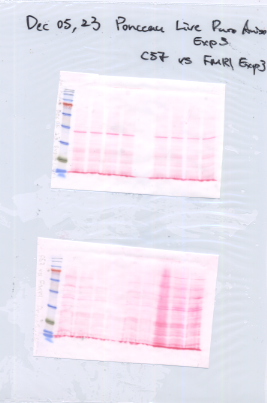

Supplement: Figure 2—source data 2. [file elife-106692-fig2-data2.zip › Figure 2 Source Data 2/Dec 5 23 Ponceau Liver Puro Aniso Comp Exp 3 C57 FMR1 Puro Aniso Comp exp 3 .tiff]

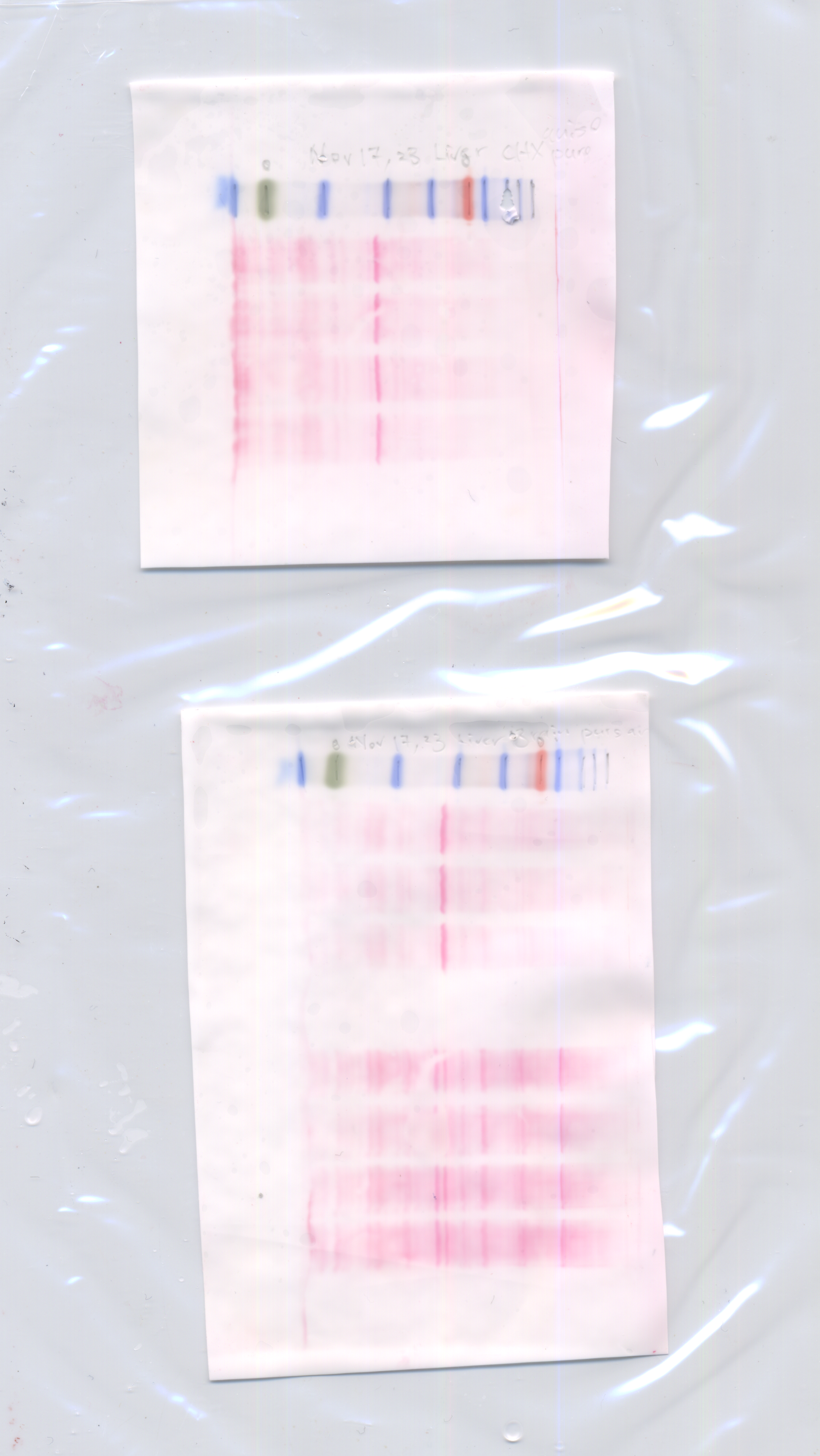

Supplement: Figure 2—source data 2. [file elife-106692-fig2-data2.zip › Figure 2 Source Data 2/Nov 17 Ponceau Liver Brain CHX Puro Aniso Comp Exp 3.tiff]

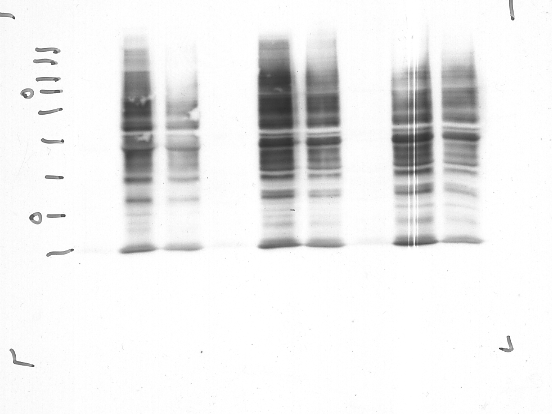

Supplement: Figure 2—source data 2. [file elife-106692-fig2-data2.zip › Figure 2 Source Data 2/Dec 14 22 anti puro I II Pellet C57 FMR1 15sec009 II.tif]

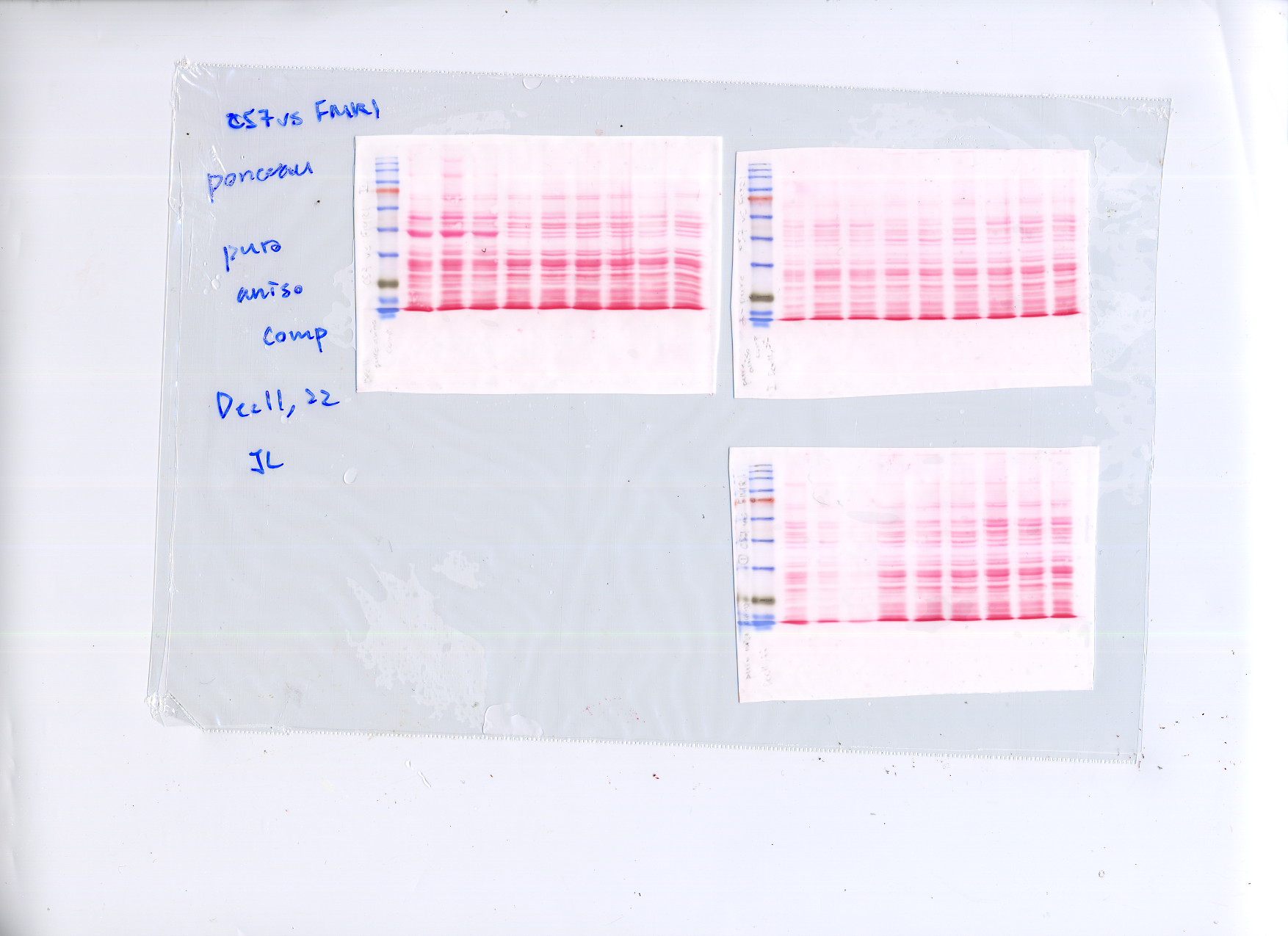

Supplement: Figure 2—source data 2. [file elife-106692-fig2-data2.zip › Figure 2 Source Data 2/dec12 c57 vs FMR1 ponceau puro aniso comp007.tif]

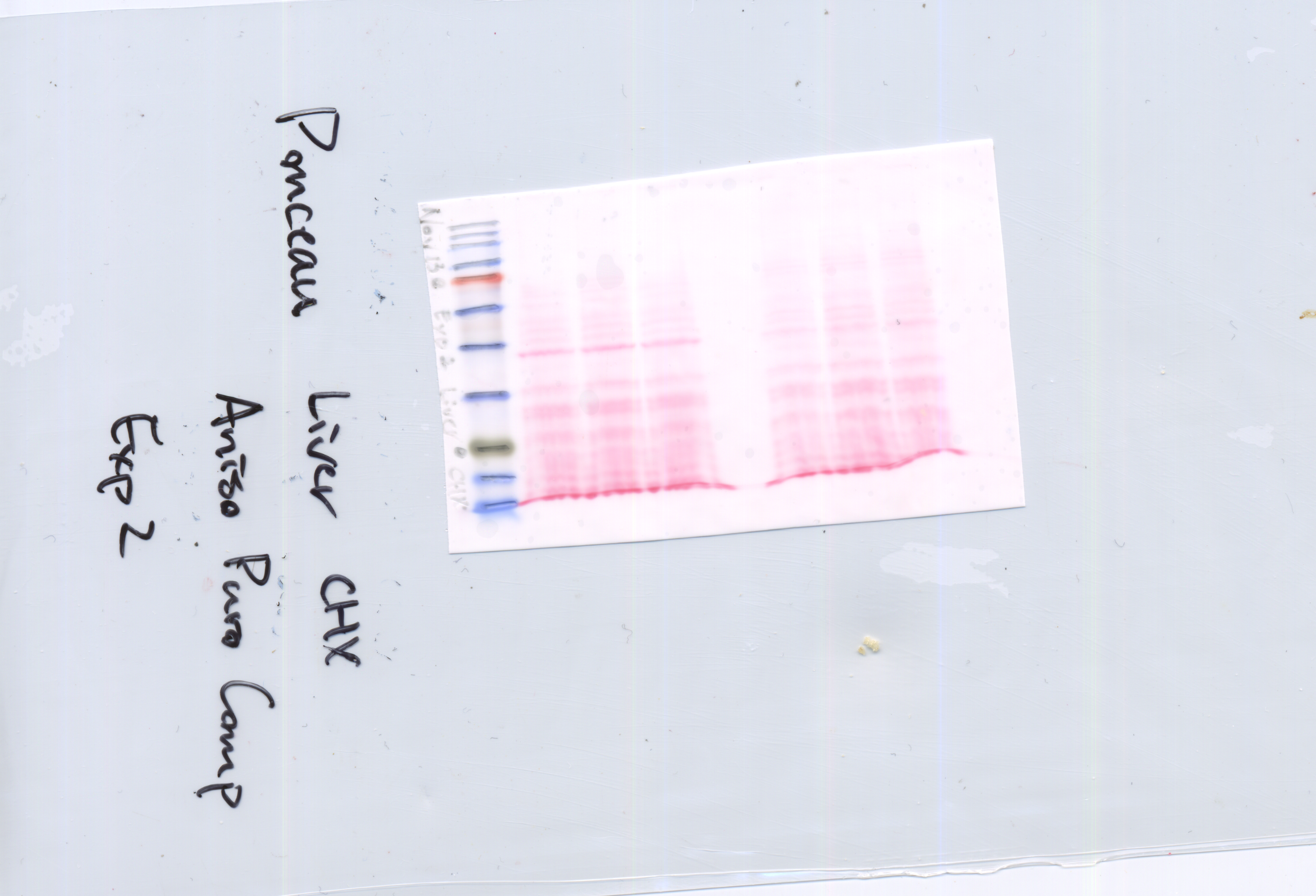

Supplement: Figure 2—source data 2. [file elife-106692-fig2-data2.zip › Figure 2 Source Data 2/Nov 14 Ponceau Liver CHX Puro Aniso Comp Exp 2 1.tiff]

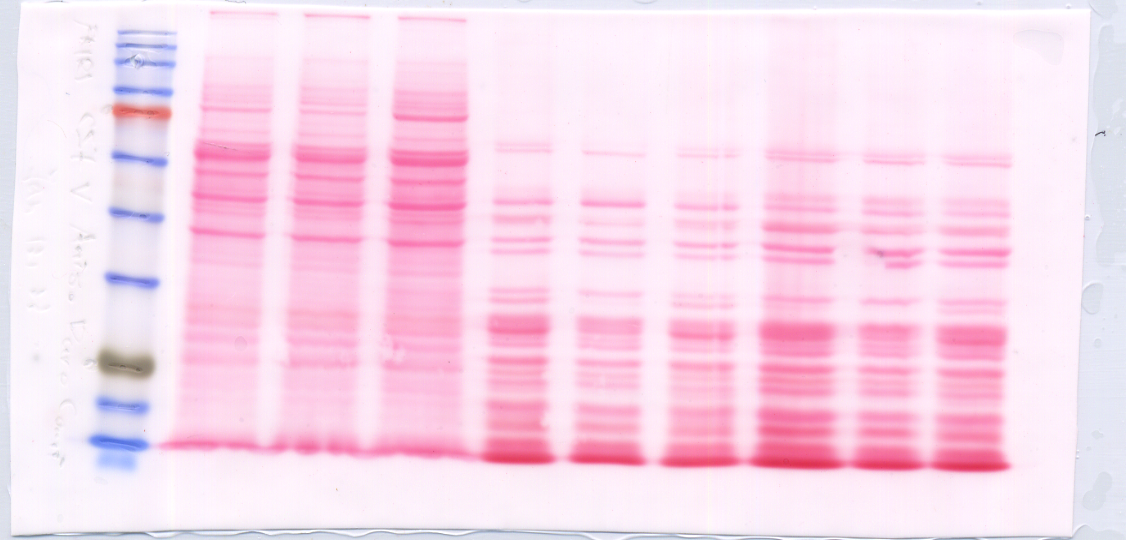

Supplement: Figure 2—source data 2. [file elife-106692-fig2-data2.zip › Figure 2 Source Data 2/Ponceau I.tif]
