# Supplementary material for: FMRP regulates neuronal RNA granules containing stalled ribosomes, not where ribosomes stall
Source: eLife. 2026 Jul 20;14:RP106692. doi: 10.7554/eLife.106692 (PMC13384498; doi:10.7554/eLife.106692)

LIVER

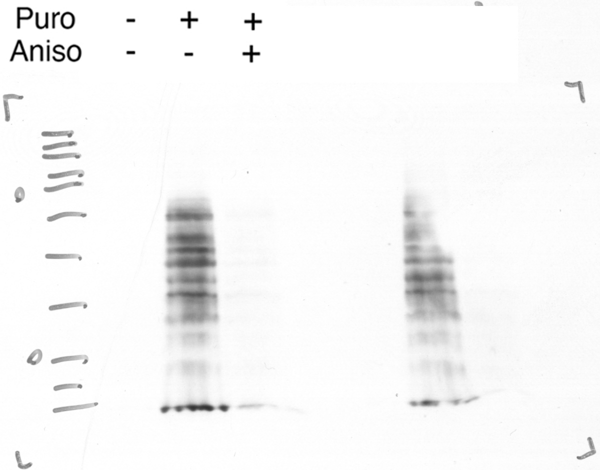

LIVER

Puro	-	+	+
Aniso	-	-	+

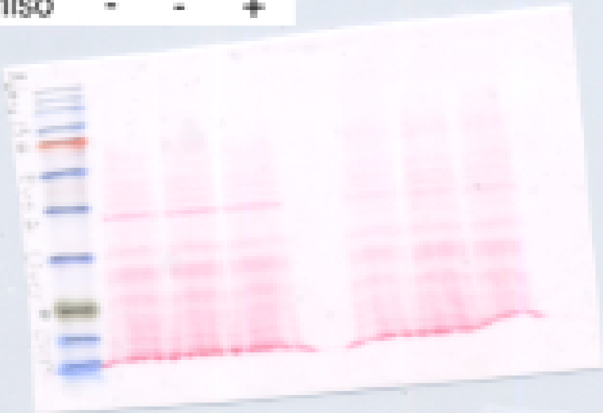

Ponceau

Liver CHX

Aniso Puro Comp

Exp 2

BRAIN

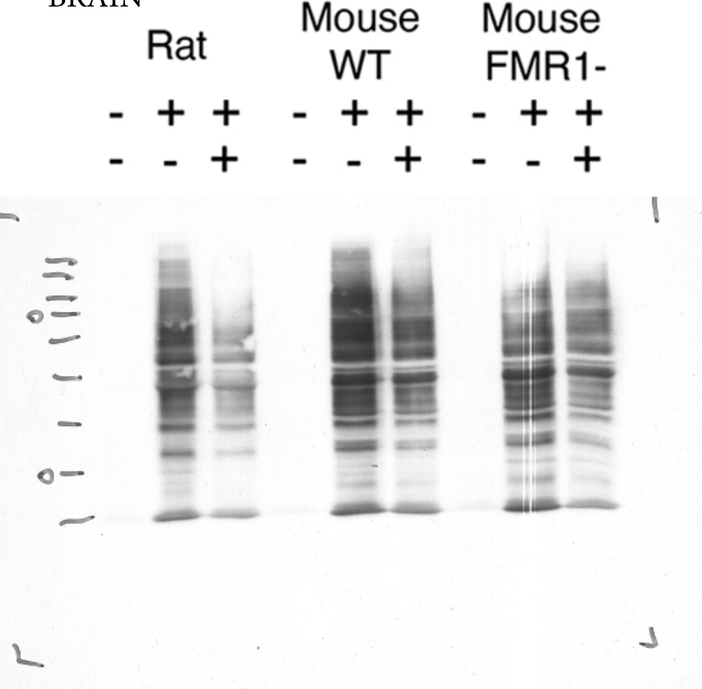

BRAIN

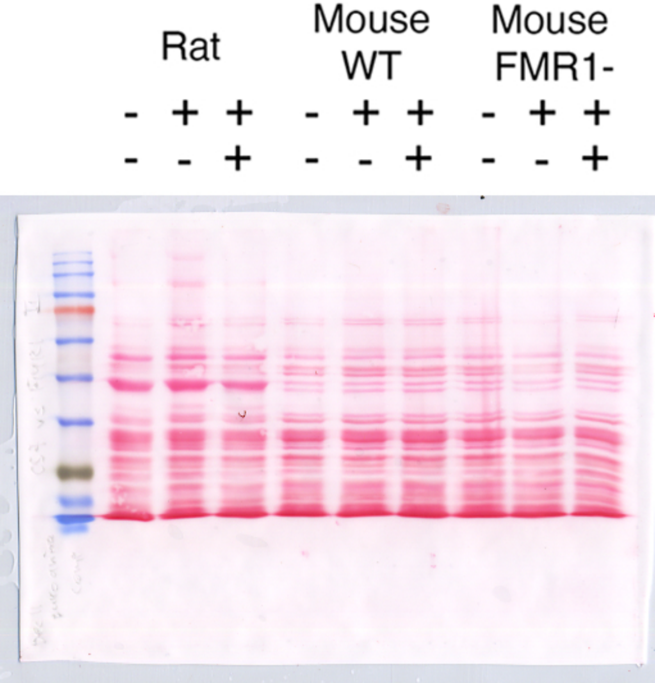

Supplement: Figure 2—source data 1. [file elife-106692-fig2-data1.pdf]
