# Supplementary material for: FMRP regulates neuronal RNA granules containing stalled ribosomes, not where ribosomes stall
Source: eLife. 2026 Jul 20;14:RP106692. doi: 10.7554/eLife.106692 (PMC13384498; doi:10.7554/eLife.106692)
Supplement: Figure 4—source data 1. [file elife-106692-fig4-data1.docx]

**Supplementary Table 4-1**. Data acquisition, reconstruction and refinement parameters and data deposition codes for the cryo-EM dataset.

|  | 80S Class 1 (A/P & P/E tRNA*) | 80S Class 2 (P/P tRNA*) | | |
| --- | --- | --- | --- | --- |
| Data Collection | | | |  |
| Microscope | Titan Krios | | |  |
| Detector | Gatan BioQuantum LS K3 | | |  |
| Nominal Magnification | 81,000x | | |  |
| Voltage (kV) | 300 | | |  |
| Total exposure (e^-^/Å^2^) | 40 | | |  |
| Number of frames | 30 | | |  |
| Defocus range (μm) | -1.25 to -2.75 | | |  |
| Calibrated physical pixel size (Å/px) | 1.09 | | |  |
| R Reconstruction and Refinement | | | |  |
| Particle number | 698,894 | | 141,803 |  |
| Resolution (Å) | Consensus map (CM): 80S (2.7Å)  Multi-body refinement (MBR): 40S (3.3Å) & 60S (2.7 Å) | | CM: 80S (3.2Å)  MBR: 40S (4.5Å) & 60S (3.2 Å) |  |
| Data Deposition |  | |  |  |
| EMDB code | EMDB-76370 | | EMDB-76379 |  |

* A/P tRNA: Aminoacyl / Peptidyl transfer RNA; P/P tRNA: Peptidyl / Peptidyl transfer RNA.
