# Supplementary material for: FMRP regulates neuronal RNA granules containing stalled ribosomes, not where ribosomes stall
Source: eLife. 2026 Jul 20;14:RP106692. doi: 10.7554/eLife.106692 (PMC13384498; doi:10.7554/eLife.106692)
Supplement: Peer review history

# Editor report: eLife Assessment

**DOI:** 10.7554/eLife.106692.3.sa0

**Authors:** Mani Ramaswami

**Affiliations:** Trinity College Dublin Ireland

**Keywords:** Solid, Valuable

Based on several lines of interesting data, the authors conclude that neuronal FMRP, which is associated with stalled ribosomes and mRNP granules, does not determine the position on the mRNAs at which ribosomes stall. They instead propose a role in subsequent translational activation of arrested mRNAs. Supported by generally **solid** experimental data, the paper represents a **valuable** contribution to the field. The generality of these conclusions, particularly for neurons of different developmental stages and for different subtypes of mRNP granules, should become clear with future studies that replicate and extend this work.

# Referee report: Reviewer #1 (Public review)

**DOI:** 10.7554/eLife.106692.3.sa1

Summary:

Authors have investigated the role of FMRP in the formation and function of RNA granules in mouse brain/cultured hippocampal neurons. Most of their results indicate that FMRP does not have a role in the formation or function of RNA granules with specific mRNAs but may have some role in distal RNA granules in neurons and their response to synaptic stimulation. This is an important work (though the results are mostly negative) in understanding the composition and function of neuronal RNA granules. the last part of the work in cultured neurons is disjointed from the rest of the manuscript and the results are neither convincing nor provide any mechanistic insight.

Strengths:

(1) The study is quite thorough, the methods and analysis used are robust and the conclusion and interpretation are diligent.

(2) The comparative study of Rat and Mouse RNA granules is very helpful for future studies

(3) The conclusion that the absence of FMRP does not affect the RNA granule composition and many of its properties in the system the authors have chosen to study is well supported by the results

(4) The difference in the response to DHPG stimulation concerning RNA granules described here is very interesting and could provide a basis for further studies though it has some serious technical issues (see below)

Weaknesses:

(1) The system used for the study (P5 mouse brain or DIV 8-10 cultured neuron) is surprising as the majority of defects in the absence of FMRP are reported in later stages (P30+ brain and DIV 14+ neurons). It is important to test if the conclusions drawn here hold good at different developmental stages.

(2) The term 'distal granules' is very vague. Since there is no structural or biochemical characterization of these granules it is difficult to understand how they are different from the proximal granules and why FMRP has an effect only on these granules.

(3) Since the manuscript does not find any effect of FMRP on neuronal RNA granules, it does not provide any new molecular insight with respect to the function of FMRP

Comments on revised version.

The authors have answered several questions raised by the reviewers. But for me, the critical issue of using only the brain from P5 animals and relatively early DIV neurons is still not convincingly addressed. FMRP may still play a role in determining the stalled ribosomes on its target mRNAs at a later stage of development, when there is more scope for activity-mediated protein synthesis.

I agree with the authors that this work helps the molecular understanding of FMRP functions by disproving one of the long-standing hypotheses.

# Referee report: Reviewer #2 (Public review)

**DOI:** 10.7554/eLife.106692.3.sa2

In the present manuscript, Li et al. use biochemical fractionation of "RNA granules" from P5 wildtype and FMR1 knock-out mouse brains to analyze their protein/RNA content, determine a single particle cryo-EM structure of contained ribosomes, and perform ribo-seq analysis of ribosome-protected RNA fragments (RPFs). The authors conclude from these that neither the composition of the ribosome granules, nor the state of their contained ribosomes, nor the mRNA positions with high ribosome occupancy change significantly. Besides minor changes in mRNA occupancy, the one change the authors identified is a decrease in puromycylated punctae in distal neurites of cultured primary neurons of the same mice, and their enhanced resistance to different pharmacological treatments. These results directly build on their earlier work (Anadolu et al., 2023) using analogous preparations of rat brains; the authors now perform a very similar study using WT and FMR1-KO mouse brains. This is an important topic, aiming to identify the molecular underpinnings of the FMRP protein, which is the basis of a major neurological disease. Unfortunately, several limitations of this study prevent it from being more convincing in its present form.

In order to improve this study, our main suggestions are as follows:

(1) The authors equate their biochemically purified "RG" fraction with their imaging-based detection of puromycin-positive punctae. They claim essentially no differences in RGs but detect differences in the latter (mostly their abundance and sensitivity to DHPG/HHT/Aniso). In the discussion the authors acknowledge the inconsistency between these two modalities: "An inconsistency in our findings is the loss of distal RPM puncta coupled with an increase in the immunoreactivity for S6 in the RG." and "Thus, it may be that the RG is not simply made up of ribosomes from the large liquid-liquid phase RNA granules."

How can the authors be sure that they are in fact analysing the same entities in both modalities? A more parsimonious explanation of their results would be that, while there might be some overlap, two different entities are analyzed. Much of the main message rests on this equivalence and I believe the authors should show its validity.

(2) The authors show that increased nuclease digestion (and magnesium concentration) led to a reduction of their RPF sizes down to levels also seen by other researchers. Analyzing these now properly digested RPFs, the authors state that the CDS coverage and periodicity drastically improved, and that spurious enrichments of secretory mRNAs, which made up one of the major fractions in their previous work, are now reduced. In my opinion this would be more appropriately communicated as a correction to their previous work, not as a main Figure in another manuscript.

(3) The fold changes reported in Figure 7 (ranging between log2(-0.2) and log2(+0.25)) are all extremely small and in my opinion should not be used to derive claims such as "The loss of FMRP significantly affected the abundance and occupancy of FMRP-Clipped mRNAs in WT and FMR1-KO RG (Fig 7A, 7B), but not their enrichment between RG and RCs".

(4) Fig 8 / S8-1 - The authors show that ~2/3 of their reads stem from PCR duplicates, but that even after removing those, the majority of peaks remains unaltered. At the same time, Fig S8-1 shows the total number of peaks to be 615 compared with 1392 before duplicate removal. Can the authors comment on this discrepancy? In addition, the dataset with properly removed artefacts should be used for their main display item instead of the current Fig 8.

(5) Fig 9 / S9-1, the density of punctae in both WT and FMR1-KO actually increases after treatment of HHT or Anisomycin (Fig S9-1 B-C). Even if a large fraction would now be "resistant to run-off", there should not be an increase. While this effect is deemed not significant, a much smaller effect in Fig 9C is deemed significant. Can the authors explain this? Given how vastly different the sample sizes are (ranging from 23 neurites in Fig S9-1 to 5,171 neurites in Fig 9), the authors should (randomly) sample to the same size and repeat their statistical analysis again to improve their credibility.

Comments on revised version.

We can see that the authors invested substantial effort to improve the manuscript and we believe it is improved.

# Referee report: Reviewer #3 (Public review)

**DOI:** 10.7554/eLife.106692.3.sa3

Summary:

Li et al describe a set of experiments to probe the role of FMRP in ribosome stalling and RNA granule composition. The authors are able to recapitulate findings from a previous study performed in rats (this one is in mice).

Strengths:

(1) The work addresses an important and challenging issue, investigating mechanisms that regulate stalled ribosomes that are part of stress granules, and focusing on the role of FMRP. This is a complicated problem, given the heterogeneity of the granules and the challenges related to their purification. This work is a solid attempt at addressing this issue, which is widely understudied.

(2) The interpretation of the results could be interesting, if supported by solid data. The idea that FMRP could control the formation and release of stress granules, rather than the elongation by stalled ribosomes is of high importance to the field, offering a fresh perspective into translational regulation by FMRP.

(3) The authors focused on recapitulating previous findings, published elsewhere (Anadolu et al., 2023) by the same group, but using rat tissue, rather than mouse tissue. Overall, they succeeded in doing so, demonstrating, among other findings, that stalled ribosomes are enriched in consensus mRNA motifs that are linked to FMRP. These interesting findings reinforce the role of FMRP in formation and stabilization of RNA granules. It would be nice to see extensive characterization of the mouse granules as performed in Figure 1 of Anadolu and colleagues, 2023.

(4) Some of the techniques incorporated aid in creating novel hypotheses, such as the ribopuromycilation assay and the cryo-EM of granule ribosomes.

Comments on revised version:

I am satisfied with the authors response to my comments.

# Author comment: Author response

**DOI:** 10.7554/eLife.106692.3.sa4

**Authors:** Jewel T-Y Li, Mehdi Amiri, Senthilkumar Kailasam, Lily Drever, Jingyu Sun, Laura Bohorquez, Nahum Sonenberg, Joaquin Ortega, Wayne S Sossin

**Affiliations:** McGill University Montreal Canada; McGill University Montreal Canada; McGill University Montreal Canada; McGill University Montreal Canada; McGill University Montreal Canada; McGill University Montreal Canada; McGill University Montreal Canada; McGill University Montreal Canada; McGill University Montreal Canada

The following is the authors’ response to the original reviews.

We have addressed all the reviewers’ comments through new experiments, additional analyses, or, in some cases, additional text. Below is a summary of the major changes in the manuscript.

(1) We have added a considerable amount of new characterization of the biochemical enrichment of the ribosome clusters, including EM of the ribosome clusters, UV absorbance profiles, immunoblots of additional targets, and additional replicates (new Figure 1). In summary, we provide better evidence that (i) the biochemical enrichment is working and (ii) that the loss of FMRP has no effect on this biological enrichment of ribosomal clusters.

(2) We have now reanalyzed all of the data in Figs. 5-8 using only the data after removing PCR duplicates from the RPFs. Other than the comparison between the nuclease treatments (Fig. 3), only this data is now used. Moreover, we have reanalyzed this data using suggestions from the reviewers, including providing PCA analysis (Fig S5-1), GSEA analysis (Fig 5), and normalizing for group size when comparing significance to total mRNAs, (Fig 6-7). We now also include a new analysis (Fig S7-1) to better explain how the loss of FMRP affects mainly FMRP targets defined by CLIP, but not all mRNAs resistant to run-off.

(3) We are now more conservative in our nomenclature; we use "pellet" instead of "RNA granule (RG)" and "fraction 5/6" instead of "ribosome clusters (RC)". We have added a section to the discussion about the relationship between the RNA granules measured using imaging of hippocampal neurites and the biochemical purification of ribosome clusters in the pellet, as requested by the reviewers.

(4) We have made many other minor changes to the text and analysis, which can be found in the specific response to the reviewers.

(5) One major additional requested change that was not implemented was to repeat our experiments at different time points. We have added a paragraph to the discussion outlining (i) why this was not done and (ii) the caveats of our conclusions without this data being present.

**Public Reviews:**

**Reviewer #1 (Public review):**

Summary:

The authors have investigated the role of FMRP in the formation and function of RNA granules in mouse brain/cultured hippocampal neurons. Most of their results indicate that FMRP does not have a role in the formation or function of RNA granules with specific mRNAs, but may have some role in distal RNA granules in neurons and their response to synaptic stimulation. This is an important work (though the results are mostly negative) in understanding the composition and function of neuronal RNA granules. The last part of the work in cultured neurons is disjointed from the rest of the manuscript, and the results are neither convincing nor provide any mechanistic insight.

Strengths:

(1) The study is quite thorough, the methods and analysis used are robust, and the conclusion and interpretation are diligent.

(2) The comparative study of Rat and Mouse RNA granules is very helpful for future studies.

(3) The conclusion that the absence of FMRP does not affect the RNA granule composition and many of its properties in the system the authors have chosen to study is well supported by the results.

(4) The difference in the response to DHPG stimulation concerning RNA granules described here is very interesting and could provide a basis for further studies, though it has some serious technical issues.

Thank you for these positive comments on the paper.

Weaknesses:

(1) The system used for the study (P5 mouse brain or DIV 8-10 cultured neuron) is surprising, as the majority of defects in the absence of FMRP are reported in later stages (P30+ brain and DIV 14+ neurons). It is important to test if the conclusions drawn here hold good at different developmental stages.

Unfortunately, myelin strongly interferes with the ability to use this protocol to purify ribosome clusters in older brains (See Khandjian et al., 2004). It is possible to redo the ribopuromycylation results at later times in culture, but since we cannot compare this to a comparable time in the brain, we have chosen not to do this experiment. We acknowledge this limitation in the discussion, noting that our results are only a snapshot of development and that different results may be observed at different times.

(2) The term 'distal granules' is very vague. Since there is no structural or biochemical characterization of these granules, it is difficult to understand how they are different from the proximal granules and why FMRP has an effect only on these granules.

We agree with the reviewer and have removed all references to distal granules. We clarified that we did not measure RPM puncta close to the neuron because the much stronger RPM signal made defining puncta more difficult, and thus, we cannot determine if there are differences between proximal and distal puncta.

(3) Since the manuscript does not find any effect of FMRP on neuronal RNA granules, it does not provide any new molecular insight with respect to the function of FMRP

We would respectfully disagree that the study does not provide molecular insight into the function of FMRP, as disproving that FMRP is important for stalling and determining the position of stalling would remove one of the major hypotheses about the function of FMRP, and showing that a major hypothesis in the literature is unlikely to be correct, is at least to me, providing insight. Moreover, we do show an effect of the loss of FMRP on the RPM puncta that represent neuronal RNA granules containing stalled ribosomes. This also provides insight.

**Reviewer #2 (Public review):**

In the present manuscript, Li et al. use biochemical fractionation of "RNA granules" from P5 wildtype and FMR1 knock-out mouse brains to analyze their protein/RNA content, determine a single particle cryo-EM structure of contained ribosomes, and perform ribo-seq analysis of ribosome-protected RNA fragments (RPFs). The authors conclude from these that neither the composition of the ribosome granules, nor the state of their contained ribosomes, nor the mRNA positions with high ribosome occupancy change significantly. Besides minor changes in mRNA occupancy, the one change the authors identified is a decrease in puromycylated punctae in distal neurites of cultured primary neurons of the same mice, and their enhanced resistance to different pharmacological treatments. These results directly build on their earlier work (Anadolu et al., 2023) using analogous preparations of rat brains; the authors now perform a very similar study using WT and FMR1-KO mouse brains. This is an important topic, aiming to identify the molecular underpinnings of the FMRP protein, which is the basis of a major neurological disease. Unfortunately, several limitations of this study prevent it from being more convincing in its present form.

In order to improve this study, our main suggestions are as follows:

(1) The authors equate their biochemically purified "RG" fraction with their imaging-based detection of puromycin-positive punctae. They claim essentially no differences in RGs, but detect differences in the latter (mostly their abundance and sensitivity to DHPG/HHT/Aniso). In the discussion the authors acknowledge the inconsistency between these two modalities: "An inconsistency in our findings is the loss of distal RPM puncta coupled with an increase in the immunoreactivity for S6 in the RG." and "Thus, it may be that the RG is not simply made up of ribosomes from the large liquid-liquid phase RNA granules."

How can the authors be sure that they are analysing the same entities in both modalities? A more parsimonious explanation of their results would be that, while there might be some overlap, two different entities are analyzed. Much of the main message rests on this equivalence, and I believe the authors should show its validity.

Thank you for your comments. We have been more conservative in the revised paper, referring to the pellet fraction as the pellet fraction rather than the RNA granule fraction to acknowledge the possibility that these two modalities differ. However, we would respectfully disagree that our main message requires RPM-labeled RNA granules in neurites and the ribosome clusters isolated by sedimentation to be “equivalent”. We do believe they are related and added a section in the discussion on this important point.

(2) The authors show that increased nuclease digestion (and magnesium concentration) led to a reduction of their RPF sizes down to levels also seen by other researchers. Analyzing these now properly digested RPFs, the authors state that the CDS coverage and periodicity drastically improved, and that spurious enrichments of secretory mRNAs, which made up one of the major fractions in their previous work, are now reduced. In my opinion, this would be more appropriately communicated as a correction to their previous work, not as a main Figure in another manuscript.

We have removed all discussion of the secretory mRNAs, as our attempts to obtain independent evidence for this finding by examining ribophorin enrichment in the pellet across different Mg^2+^ concentrations did not support this interpretation (data not shown in the paper). I understand that the change in nuclease is somewhat out of place narratively, but it is clearly relevant to this work. We would disagree with our previous work requiring a ‘correction’. We believe that the nuclease resistance of the mRNA at the entrance site is important. We reproduce our results from rats with similar nuclease treatment in mice as seen in our previous publication; thus, this work is not wrong. We have a paper in preparation that suggests the secondary structure of the mRNA at this location may be important for stalling and thus feel strongly that this result should remain in the manuscript.

(3) The fold changes reported in Figure 7 (ranging between log2(-0.2) and log2(+0.25)) are all extremely small and in my opinion should not be used to derive claims such as "The loss of FMRP significantly affected the abundance and occupancy of FMRP-Clipped mRNAs in WT and FMR1-KO RG (Fig 7A, 7B), but not their enrichment between RG and RCs".

We agree that the changes are small and indeed did not appear in the DEG analysis. However, because we are analyzing a large set of mRNAs in this analysis, the results are highly significant and remain significant when using the new statistical tests suggested by the reviewer below. We now emphasize that these are small changes and remind readers that none of the individual mRNA changes were significant in the DEG analysis.

(4) Figure 8 / S8-1 - The authors show that ~2/3 of their reads stem from PCR duplicates, but that even after removing those, the majority of peaks remain unaltered. At the same time, Figure S8-1 shows the total number of peaks to be 615 compared with 1392 before duplicate removal. Can the authors comment on this discrepancy? In addition, the dataset with properly removed artefacts should be used for their main display item instead of the current Figure 8.

We now use only the data after removing PCR duplicates for all the analyses except in Figure 3. The number of peaks observed is determined mainly by the threshold used, as stated in the methods “To be identified as a peak, the zenith of an abundance site for the reads must be 4x higher of the average of the total transcript.” Due the lower number of reads after the PCR duplicates fewer peaks reached this threshold.

(5) Figure 9 / S9-1, the density of punctae in both WT and FMR1-KO actually increases after treatment of HHT or Anisomycin (Figure S9-1 B-C). Even if a large fraction would now be "resistant to run-off", there should not be an increase. While this effect is deemed not significant, a much smaller effect in Figure 9C is deemed significant. Can the authors explain this? Given how vastly different the sample sizes are (ranging from 23 neurites in Figures S91 to 5,171 neurites in Figure 9), the authors should (randomly) sample to the same size and repeat their statistical analysis again, to improve their credibility.

The box and whisker plots emphasize the median and not the average. We now also show the averages in Figure S9-1, which indicate a slight decrease for both HHT and anisomycin.

We apologize for the typo in the figure legend in Figure 9, 171, not 5171. We now use random sampling in Figures 6 and 7, where the sample sizes differ substantially.

**Reviewer #3 (Public review):**

Summary:

Li et al describe a set of experiments to probe the role of FMRP in ribosome stalling and RNA granule composition. The authors are able to recapitulate findings from a previous study performed in rats (this one is in mice).

Strengths:

(1) The work addresses an important and challenging issue, investigating mechanisms that regulate stalled ribosomes that are part of stress granules, and focusing on the role of FMRP. This is a complicated problem, given the heterogeneity of the granules and the challenges related to their purification. This work is a solid attempt at addressing this issue, which is widely understudied.

(2) The interpretation of the results could be interesting if supported by solid data. The idea that FMRP could control the formation and release of stress granules, rather than the elongation by stalled ribosomes, is of high importance to the field, offering a fresh perspective into translational regulation by FMRP.

(3) The authors focused on recapitulating previous findings, published elsewhere (Anadolu et al., 2023) by the same group, but using rat tissue, rather than mouse tissue. Overall, they succeeded in doing so, demonstrating, among other findings, that stalled ribosomes are enriched in consensus mRNA motifs that are linked to FMRP. These interesting findings reinforce the role of FMRP in the formation and stabilization of RNA granules. It would be nice to see extensive characterization of the mouse granules as performed in Figure 1 of Anadolu et al., 2023.

(4) Some of the techniques incorporated aid in creating novel hypotheses, such as the ribopuromycilation assay and the cryo-EM of granule ribosomes.

Thank you for these positive comments. We have now added a more extensive characterization in Figure 1.

Weaknesses:

(1) The RNA granule characterization needs to be more rigorous. Coomassie is not proper for this type of characterization, simply because protein weight says little about its nature. The enrichment of key proteins is not robust and seems not to reach significance in multiple instances, including S6 and UPF1. Furthermore, S6 is the only proxy used for ribosome quantification. Could the authors include at least 3 other ribosomal proteins (2 from the small, 2 from the large subunit)?

We have increased N to improve the robustness of the enrichment analysis and added several additional RBPs. Along with Coomassie we now include analysis of UV absorbance and include EMs from these fractions showing the presence of 80S ribosomal clusters in the fractions we are using.

(2) Page 12-13 - The Gene Ontology analysis is performed incorrectly. First, one should not rank genes by their RPKM levels. It is well known that housekeeping genes, such as those related to actin dynamics, molecular transport, and translation, are highly enriched in sequencing datasets. It is usually more informative when significantly different genes are ranked by p-adjust or log2 Fold Change, then compared against a background to verify enrichment of specific processes. However, the authors found no DEGs. I would suggest the removal of this analysis and the incorporation of a gene set enrichment analysis (ranked by p-adjust). I further suggest that the authors incorporate a dimensionality reduction analysis to demonstrate that the lack of significance stems from biology and not experimental artifacts, such as poor reproducibility across biological replicates.

Thank you for the suggestion. We now use GSEA analysis to examine differences in gene sets between WT and FMR1- mice and find some significant changes (new Fig. 5). The old analysis is still included for comparison to our earlier paper as a supplemental figure. We have now included a PCA analysis (FigS5-1) to show reproducibility across biological replicates.

**Recommendations for the authors:**

**Reviewer #1 (Recommendations for the authors):**

(1) RNA sequencing comparison between WT and FMR1 KO mice should be carried out at a later developmental stage, which may provide a better difference between these two groups

There are a number of studies that have already done this analysis and in specific brain regions 10.1016/j.neuron.2017.07.013; 10.7554/eLife.46919; 10.3389/fnmol.2017.00340; https://doi.org/10.1016/j.neuron.2023.06.009. The main goal of our RNA-seq was to standardize for the RPF studies, not to identify differences in RNA-seq between WT and FMRP. In the response to public review point 1 we explain why we do not look at later developmental timepoints.

(2) The same is true in characterizing the effect of FMRP on the RNA granules.

See response to public review point 1, which addresses this point.

(3) No evidence is provided for the effectiveness of DHPG stimulation in DIV8-10 neurons; this is needed for justification using neurons at this stage.

We have previously shown that DHPG stimulation in these neurons at this developmental time from cultures made from rat brain is sufficient to decrease the number of RPM puncta and to induce an increase in the synthesis of proteins in an initiation resistant manner (Graber et al, 2013; Graber et al, 2017). This is now more clearly stated in the manuscript. Moreover, here we replicate the result of DHPG in WT mice at reducing the number of RPM puncta.

(4) In Figure 9 B, it is not clear whether the neurites indicated are axons or dendrites. Since neurons are still in the early stages of dendritogenesis/synaptogenesis, it is important to make that distinction.

We have previously characterized RNA granules in axons and dendrites in hippocampal cultures from rats at this time (Miller et al, 2009, MCN 40:485-495) and they are similar. While it is likely that the vast majority of the neurites at this time are dendrites, since we did not use markers, we conservatively just use the term neurites.

(5) In Figure 1 (and elsewhere), fraction 5/6 is used as a polysome or RNA cluster. The authors have not provided a UV absorption profile and only have s6 as evidence to say this polysome. In the Coomassie gel, this fraction is any different than fractions 7/7 or 9/10; what is the justification for using this fraction?

The main justification for these fractions is to be consistent with our previous paper (Anadolu et al, 2023) and the Khandian study comparing polysomes to pellet using the same fractionation protocol (El-Fatimy et al, 2016). We now provide a UV absorption profile (Fig. 1C) and EM pictures (Fig. 1D) to show the ribosome clusters in this fraction. We do not believe our results would be fundamentally different from those obtained if we had used other heavy fractions.

Minor comments

(1) The font size very small in the figures, please increase it.

We have worked hard to increase the font size in all the figures.

(2) In the result section for Figure 3B - it is written 'majority of these mRNA are non-coding mRNA' - this doesn't make sense.

Corrected

**Reviewer #2 (Recommendations for the authors):**

(1) There are lots of mistakes (e.g. word omissions or duplications, grammatical errors) throughout the text, too many to list here.

We have carefully edited the text to try to minimize these mistakes.

(2) In many positions related to their improved nuclease digestion protocol, samples are labelled "M ...", which apparently stands for "high magnesium and high nuclease treatment group". I would suggest switching to something more intuitive, such as "... (improved digestion)".

We have removed most of the comparisons between these samples. What remains (Figure 3), we just use Low Nuclease when we refer to the sample with low Magnesium and low nuclease.

(3) Figure 1,3 - It would be tremendously illuminating to see a polysome trace (UV260 absorbance) in addition to Coomassie-stained SDS-PAGE to underscore the interpretation of the different fractions by the authors. As it stands, there is no way of telling whether there are any polysomes present at all. This can also be done by hand using a UV absorption reader if no built-in device is available to the authors.

We have now done this (Fig. 1C) and also provided EM of this fraction to show the presence of ribosomes in this fraction.

(4) I don't understand why the authors switched from calling fraction 5/6 the "polysome fraction" in their previous work to calling it "ribosome cluster fraction" in this work. The argument given "[...] due to its structural similarity to ribosomes in RNA Granules (Anadolu et al., 2023), we conservatively call this the ribosome cluster fraction (RC)." does not instill confidence that these two fractions are indeed distinct.

We agree with the reviewer and regret this decision. We now call the pellet, the pellet and Fraction 5/6, fraction 5/6.

(5) Figure 1C - There are clear scanning or compression artefacts in the blot images (most prominently in the eEF2 lanes) that should be corrected.

We have replaced all images in Figure 1 and have increased the N of this experiment considerably.

(6) Figure 1C - The authors claim that WT mouse RG is enriched in FMRP compared to RC or starter fraction, but there is also a lot more protein loaded in the RG (especially when compared to RC). It is also hard to believe from the Coomassie staining that despite the much stronger presence of low MW bands (which is where ribosomal proteins migrate) in fraction 5/6, the s6 western blot signal is actually comparable between RC and RG. Can the authors please provide more detail on the loading of these fractions and supply quantification of FMRP in all three fractions, normalized by total protein? This might also be the source of their discrepancy, stating that contrary to their expectation, ribosomes (as measured by s6 signal / s6 signal in starter fraction) are actually increased in FMR1-KO brains.

We have repeated all of these experiments and changed our method of quantification (See methods). We no longer use the starting material in our quantification. Indeed, with the additional data and change in method, we no longer see an increase in S6 in the FMR1- pellet fraction.

(7) Figure 1 - I believe "D-F" should only read "D-E" based on the axis titles, and instead "FG" should be added before the next sentence. Instead of "Staufen" it should be specified in the Figure that "Stau2" was quantified. "Staufen (59kd)" should read "Stau2 (59 kDa)" and "anti-Staufen (52kb)" should read "anti-Stau2 (52 kDa)" and the same for all other similar instances. It is further hard to believe that e.g., "Staufen2 (59kd)" (see above) is not significantly enriched with N=5, a very low spread, and over 1.5x enrichment. The authors should double-check that the appropriate statistical test was employed.

Figure 1 has been completely redone, and the two Staufen bands are enriched in this new analysis.

(8) Figure S4-2 - Most of the detail in the corresponding figure legend should be moved to the Materials and Methods section.

Details relevant to the methods in this figure legend have been now moved to the Material and Methods section.

(9) Figure 4A - The displayed/segmented tRNA densities appear unusually distorted. I would recommend displaying segmented densities of the original homogeneous reconstructions, not of separated and later fused partial maps.

Figure 4 was modified according to the suggestions of this reviewer.’

(10) Figure 9 C-D, S9-1 B-E - Are not all conditions also including puromycin as in B above? If so, it should be added to both the figure and the figure legend.

The reviewer is correct and the figure and legend has been changed to reflect this.

**Reviewer #3 (Recommendations for the authors):**

(1) "Loss of FMRP causes Fragile X syndrome. In humans, the loss of FMRP occurs due to the expansion of a CGG repeat in the 5' untranslated region (UTR) of the gene, leading to excessive methylation and transcriptional inhibition."

Comment: Genes don't have 5'UTR, but exons encoding 5'UTR. I suggest rephrasing this statement.

This sentence has been rephrased.

(2) "Several of these functions have been implicated in Fragile X syndrome, including FMRP's regulation of miRNA repression, splicing, translation initiation, and translational elongation".

Comment: Is this a typo? miRNA instead of mRNA?

No, this is correct. FMRP has been implicated in the regulation of microRNAs (miRNAs) in a number of studies.

(3) "elongation rates are also increased in mouse models of FMRP".

Comment: Mouse models of Fragile X?

This has been corrected.

(4) "Parts of this work were included in the Master's thesis of the first author (Li, 2024)."

This has been removed.

(5) Comment: Graphs in Figure 1 need proper y-axis labeling. What is the normalization method? What are the values presented in the y-axis?

Figure 1 has been completely changed and the Y-axes are now clear in this new version.

(6) "Thus, by looking at the percentage of puromycylation present in the presence of anisomycin, we can estimate the number of ribosomes in this state. "

Comment: Are the authors really estimating the number of ribosomes in a resistant state? One could argue that they are collecting populational information regarding resistance to anisomycin.

We have rephrased this sentence to be more conservative about what we are measuring.

(7) Comment: Page 11 - Why did the authors assume magnesium would affect the conformation state of the ribosomes? What is the rationale behind increasing the [Mg2+]?

Most preparations using ribosomes use 10 mM MgCl_2_. However, most neuroscientists use physiological buffers that contain 2.5 mM MgCl_2_. In bacteria, this makes a large difference, but evidence from eukaryotes is not clear. Since this is a collaboration between these two schools of thought, we decided to switch to 10 mM MgCl_2_, since in the EM, there were some free 60S ribosomes (Anadolu et al, 2024).

(8) Page 11- "In other words, high Mg2+ decreased the abundance of mRNAs normally cotranslationally inserted into the ER which are unlikely to be components of transporting RNA granules containing stalled ribosomes and solidified our focus on the M protocol in the analyses below."

We have removed this from the paper, as additional experiments aimed to solidify this interpretation failed to detect an effect on secretory mRNAs.

(9) Comment: The whole "abundance", "enrichment", and "occupancy" nomenclature is hard to follow.

We have rewritten this section.

(10) Page 13 - "There were only 2 protein coding genes that were significantly different between the abundance of FMR1-KO and WT in protein coding genes - FMR1 and Wdfy1 (Extended Data Table 5-2). There were no significantly different genes between WT and FMR1-KO occupancy and enrichment. Thus, no difference rose to significance, given the large number of mRNAs used in this analysis."

Comment: It seems like this is repeating the same information three times.

This has been changed.

(11) Page 13 - "Similar to previous experiments with rats, the most abundant mRNAs resistant to run off were significantly abundant, occupied and enriched in both WT and FMRP RPFs (Fig 6)"

The Shah et al dataset we use was based on the most abundant mRNAs resistant to run-off. While we agree it is not surprising that they are also abundant in the pellet we observe, this would not necessarily be true unless the pellet is actually enriched in stalled mRNAs.

(12) Page 14 - "These mRNAs had been identified by cross-linking FMRP with mRNA, fragmenting the mRNA, immunoprecipitating the mRNA still associated with FMRP and sequencing this mRNA."

We shortened this description.

(13) Page 14 - "Interestingly, while still significant, there appeared to be a decrease in the relative abundance of these mRNAs in the FMR1-KO RG (Fig 6B)"

Comment: It is hard to observe this decrease in the boxplots. Second, the statistical tests for the bioinformatics analyses are not the most appropriate, given the large discrepancy in the number of mRNAs present in the experimental group ("All mRNAs") and the filtered groups.

We have redone the statistics using multiple random sampling of all the mRNAs such that the total number of mRNAs in the group was the same. This lowered the significance for some groups, but they are mostly still highly significant. This analysis has also been affected by switching to using the data from the PCR-subtracted RPFs. The changes we now observe are more evident in the whisker box plots due to this improvement in the data.

(14) Page 16 - "To rule out that peaks were due to amplification artifacts in the preparation of RPFs we repeated these analyses after removing PCR duplicates (Fig. S8-1; Extended Data Table S8-3) and found over 95% of the peaks identified without removing PCR duplicates were defined as a peak in at least one of the biological replicates after removing duplicates. More importantly, we found similar results with enrichment of FXS motif and enrichment of negatively charged amino acids in the FMR1-KO only, WT only and both peaks after removing PCR duplicates (Fig. S8-1; Extended Data Table S8-3)."

Comment: It is unclear why the authors needed to include the analysis without PCR duplicate removal. This is an essential step to guarantee the robustness of ribo-seq findings. I recommend removing the whole analysis from Figure 8 from the manuscript and including only the post-duplicate removal analysis.

As mentioned above, we completely agree with this statement and now show only this data and moreover have redone all the figures with only this data (except for Fig. 3).

(15) Figure 9 - I am unsure that the data is convincing enough to demonstrate reinitiation of mRNA granules induced by DHPG. I suggest a colocalization experiment with another protein well known to be localized to RNA granules, such as G3BP1. In addition, repeat the experiment with an additional group where elongation is blocked after the addition of DHPG, which presumably would prevent the reduction in the WT puncta density.

These are interesting additional experiments, but outside the scope of what we can manage. We have previously shown colocalization of Staufen, FMRP and UPF1 to these puncta (Graber et al, 2013; Graber et al, 2017) and shown that these puromycylated puncta also colocalize with nascent peptides detected using the Sun-Tag technique. While we think doing the experiment in the presence of an elongation inhibitor would be interesting, we disagree that it would prevent the reduction in WT puncta density, since we believe what is happening is the loss of the liquid-liquid phase separation of the ribosome clusters due to dephosphorylation of RBPs like FMRP and UPF1 (Graber et al, 2017), and this would reduce the puncta density whether or not the ribosomes were activated for translation.

Nevertheless, we have tried to temper the conclusions made from this result, emphasizing what we know (RPM puncta are decreased) as opposed to actual reactivation of stalled polysomes which we are not measuring.

Discussion - Page 18 - "Nevertheless, if FMRP binding was the critical determinant for presence in neuronal RNA granules, we would have expected to observe more differences." This is not true. If the data is poorly collected, you will not see differences.

This statement was removed.

(16) "A proportion of the stalled ribosomes that are not stored in large RNA granules may still be pelleted in the sucrose gradients. This fraction may be greater in the absence of FMRP."

Comment: The authors are right about this and touch on my original point that the characterization of the biochemical fractionation is not convincing enough. I'd suggest probing against more proteins that are contained in RNA granules.

We have added several proteins to the biochemical characterization shown in Figure 1. We have added a discussion about the relationship between neuronal RNA granules and the sedimented pellet fraction in the discussion section.
